# Discovery of novel dual-active 3-(4-(dimethylamino)phenyl)-7-aminoalcoxy-coumarin as potent and selective acetylcholinesterase inhibitor and antioxidant

**DOI:** 10.1080/14756366.2019.1571270

**Published:** 2019-02-06

**Authors:** Gabriela Alves de Souza, Soraia John da Silva, Catarina de Nigris Del Cistia, Paulo Pitasse-Santos, Lucas de Oliveira Pires, Yulli Moraes Passos, Yraima Cordeiro, Cristiane Martins Cardoso, Rosane Nora Castro, Carlos Mauricio R. Sant’Anna, Arthur Eugen Kümmerle

**Affiliations:** a Programa de Pós-Gradução em Química (PPGQ), Universidade Federal Rural do Rio de Janeiro, Rio de Janeiro, Brazil;; b Laboratório de Diversidade Molecular e Química Medicinal (LaDMol-QM, Molecular Diversity and Medicinal Chemistry Laboratory), Departament of Chemistry, Universidade Federal Rural do Rio de Janeiro, Rio de Janeiro, Brazil;; c Faculdade de Farmácia, Universidade Federal do Rio de Janeiro, Rio de Janeiro, Brazil

**Keywords:** Coumarins, cholinesterase, antioxidant, bioisosterism

## Abstract

A series of 3-substituted-7-aminoalcoxy-coumarin was designed and evaluated as cholinesterase inhibitors and antioxidants. All compounds were effective in inhibiting AChE with potencies in the nanomolar range. The 3-(4-(dimethylamino)phenyl)-7-aminoethoxy-coumarin (**6a**) was considered a hit, showing good AChE inhibition potency (IC_50_ = 20 nM) and selectivity (IC_50_ BuChE/AChE = 354), quite similar to the reference drug donepezil (IC_50_ = 6 nM; IC_50_ BuChE/AChE = 365), also presenting antioxidant properties, low citotoxicity and good-predicted ADMET properties. The mode of action (mixed-type) and SAR analysis for this series of compounds were described by means of kinetic and molecular modeling evaluations.

## Graphical Abstract

**Figure:**
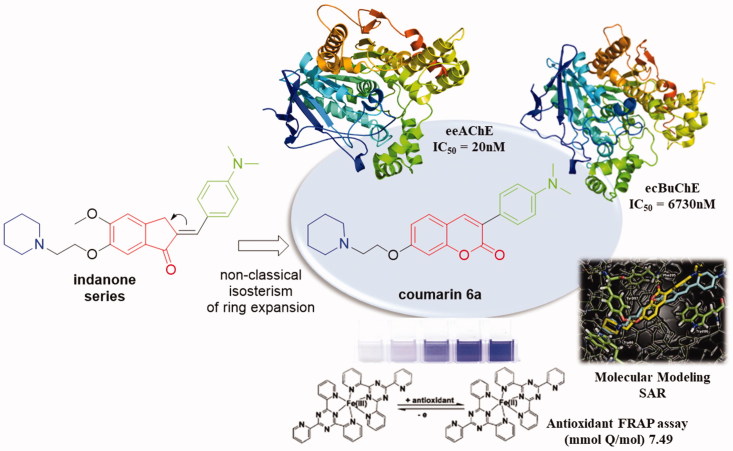

## Introduction

Alzheimer's disease (AD) is the most frequent case of age-related neurodegenerative dementia, characterized by progressive loss of memory and other cognitive functions^1^ ^,^ ^2^. AD is a heterogeneous disease, driven by the interaction between multiple deleterious factors. However, the exact mode of how these factors contribute to impair neuronal functions and neuronal survival still remains undetermined. One of the main markers of AD is the accumulation of β-amyloid plaques (Aβ) in nerve cells. In healthy brain, these aggregates of proteins are degraded and eliminated^3^. However, in AD the aggregates accumulate to form insoluble plaques^3^. Another characteristic is the presence of insoluble neurofibrillary filaments that is associated with tau protein (PTau)^4^. In AD, however, PTau becomes hyperphosphorylated, denaturing and resulting in its dissociation of microtubules, followed by formation of neurofibrillary filaments that aggregate, acting as physical barriers to microtubules^4^. In addition, the occurrence of glial cell neuroinflammation, synaptic loss, and specific neuronal death is common in AD^5^ and can be aggravated by oxidative stress^6^.

The knowledge of neurotransmitter disorders in AD has led to the approval of drugs with symptomatic effects^7^. The cholinergic hypothesis of AD states that the degeneration of cholinergic neurons in basal forebrain nuclei causes disorders in the presynaptic cholinergic terminals in the hippocampus and neocortex, which are regions of extreme importance for memory disorders and other cognitive symptoms^8^. Because of neurodegeneration, the activity of cholinergic neurons and levels of neurotransmitter ACh are reduced. One approach to improve cholinergic neurotransmission is to increase the availability of ACh by inhibition of acetylcholinesterase^9^.

Acetyl (AChE) and butyrylcholinesterase (BuChE) inhibitors are the main drugs for the clinical treatment of AD in the initial to moderate stage^10^. Galantamine and donepezil are selective inhibitors of AChE, whereas rivastigmine inhibits AChE and BuChE with similar affinities. Selective AChE inhibitors have demonstrated better therapeutic effects when compared to nonselective inhibitors^11^ since BuChE is also associated with drug metabolism and detoxification, lipoprotein metabolism and diseases^12^. Thus, our objectives herein were the design, synthesis and pharmacological evaluation of novel 3-substituted-7-aminoalcoxy-coumarins as selective inhibitors of AChE and antioxidant, based on a previously described indanone series^13^.

## Materials and methods

### General procedure for the synthesis of 2a–d

In a reactional borosilicate tube, 10–15 mmol of dibromoalkanes (4–6 eq.) and 5 mmol (2 eq.) of K_2_CO_3_ were solubilized in 2 ml of acetone (Scheme 2). To this stirred suspension a solution of 2.5 mmol of 7-hydroxycoumarin (**1**) in 8 ml of acetone was added dropwise. Thereafter, the reactional tube was sealed and the reaction was kept at 60 °C and stirred for 6–12 h. After reaction completion, acetone was evaporated and the crude reaction partitioned with distilled water and ethyl acetate. The final slurry was precipitated in hexanes under ultrasound irradiation and filtered off.

### General procedure for the synthesis of 3a–d

To a stirred solution of 1.7 mmol of the respective *O*-alkyl coumarin derivative (**2a–d**), 5 mmol (3 eq.) of sodium acetate in 8 ml of glacial acetic acid and 2.1 mmol (1.3 eq.) of Br_2_ were slowly added (Scheme 2). The reaction was stirred at room temperature for 2 h. After reagent consumption, the reaction mixture was poured to a beaker containing crushed ice. The formed precipitate was filtered off under vacuum and purified by silica gel column chromatography (hexanes: dichloromethane mixture, 50–90% gradient elution).

### General procedure for the synthesis of 4a–d

In a reactional vessel, 1.3 mmol of the respective 3-bromo-7-(bromoalkoxy)coumarin derivatives (**3a–d**) and 3.9 mmol (3 eq.) of piperidine were dissolved in 8 ml CH_3_CN (Scheme 2). The reaction was kept under stirring at 60 °C for 3–8 h. Acetonitrile was evaporated in a rotary evaporator and the respective products purified by silica gel column chromatography (dichloromethane: methanol, 0–25% gradient elution).

### General procedure for the synthesis of 5a–c and 6a–c

In a reaction borosilicate tube, 0.14 mmol of the corresponding derivative (**4a, 4b and 4d**), 0.20 mmol (1.4 eq.) of appropriate phenylboronic acid and 0.42 mmol (3 eq.) of K_2_CO_3_ were solubilized in 4 ml of a solvent mixture (water: ethanol: toluene (2:1:1)) (Scheme 2). The reaction was degassed with N_2_ then 0.01 mmol (7 mol%) of Pd(PPh_3_)_4_ catalyst added. The reaction tubes were sealed and the mixtures were subjected to magnetic stirring and heating at 65 °C for 3–5 h. At the end of the reaction, the solvent mixture was evaporated in a rotary evaporator and the respective products purified by silica gel column chromatography (dichloromethane: methanol, 0–25% mixture gradient elution).

### Cholinesterase inhibition and kinetics assays

Activity of enzymes and inhibition kinetics were determined using a Bio-Rad iMark microplate reader based on a modification of the Ellman method.^14^ ^,^ ^15^ Compounds were dissolved in DMSO. The assay solution which contained 60 µL 5,5′-Dithiobis(2-nitrobenzoic acid) (DTNB) at 1.1 mM, 30 µL AChE/BuChE at 0.20 U/mL (initial concentration) and 150 µL tested compound solution with different concentrations. Absorbance was then recorded at *λ* = 415 nm. After 10-min incubation at 30 °C, 24 µL acetylthiocholine iodide/*S*-butyrylthiocholine iodide (at 2.75 mM for activity inhibition assay and 2.75–0.44 mM for kinetic study assay) were added and the absorbance recorded after a 10-min incubation (for activity inhibition assay) or after 0–20 min incubation (for kinetic study assay) at 30 °C.

### Molecular modeling

For EeAChE (*Electrophorus electricus*), the PDB structure 1C2O was used; for EqBuChE (*Equus caballus*), a 3 D homology model was necessarily built from a sequence available in the UniProtKB/Swiss-Prot (entry Q9N1N9) with the automated mode of the protein structure homology-modeling server, Swiss-Model^16^, using as template the human BuChE (PDB 4TPK)^17^. Spartan’14 program [Wavefunction, Inc.] was utilized to construct and optimize the inhibitors with the PM6 method^18^. The program GOLD 5.6 (CCDC Software Ltd., Cambridge, UK) was used to for the docking study with the GoldScore scoring function^19^.

### Evaluation of the antioxidant activity by the ferric reducing ability of plasma (FRAP) method

A 0.5 ml solution of coumarin compounds in methanol (50 µM final concentration) was mixed with 4.5 ml of the FRAP reagent. After 10 min of incubation at 37 °C, absorbance at 593 nm was measured using methanol as blank.^20^ ^,^ ^21^ The calibration curve was prepared with quercetin and the results expressed as: antioxidant index based on quercetine (Q) (mmol Q/mol). The analyses were performed in triplicate.

### Murine neuroblastoma cell (N2a) culture and cell viability assay

N2a cells were cultured in Dulbecco’s modified Eagle’s medium supplemented with 10% fetal bovine serum and 0.1% gentamicin in a 5% CO_2_ atmosphere. N2a cells were transferred to a 96-well plate (∼10,000 cells/cm^2^) and incubated for 24 h, before treatment with the compounds at 10 or 50 µM. Cell viability was evaluated by MTT (3-[4,5-dimethylthiazol-2-yl]-2,5-diphenyl tetrazolium bromide) assay.

## Results and discussion

### Compounds design and synthesis

**Scheme 1.**
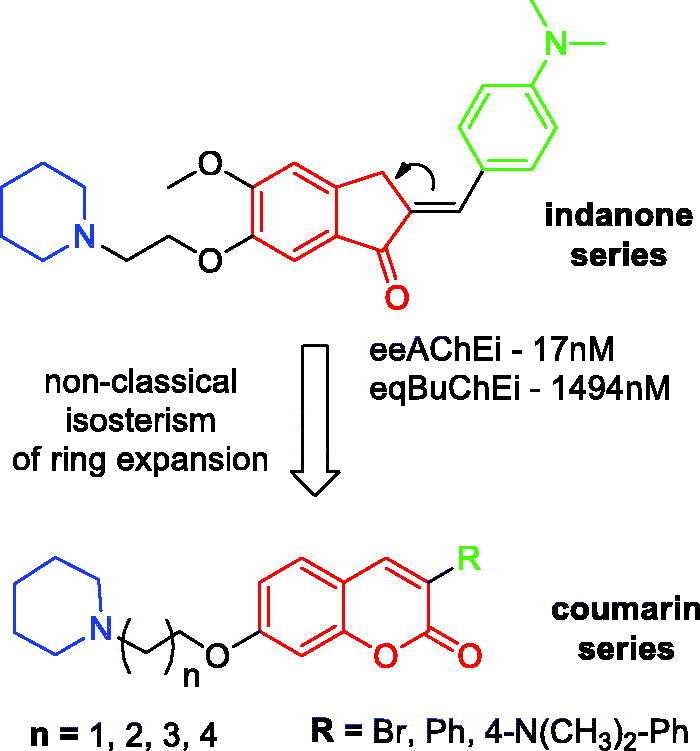
Design of alkylamino-coumarin cholinesterase inhibitors series.

The design of the novel alkylamino-coumarin derivatives (Scheme 1) was based on the structural requirements for mixed-type selective AChE inhibition present in alkylamino-indanone inhibitor recently described^13^, as well as on the widespread use of coumarins for this pharmacological activity^22^ ^,^ ^23^. The coumarin series was based on: 1- the maintenance of the cyclic alkylamino group, which is responsible for the interaction with the cationic catalytic site (CAS) of AChE, exploring different lengths of methylene linkers (2–6); 2-exchange of the indanone nucleus by the coumarin through non-classical isosterism of ring expansion^24^; 3- use of hydrophobic groups at position 3 of coumarin, targeting interactions with the peripheral anionic site (PAS) of AChE.

**Scheme 2.**
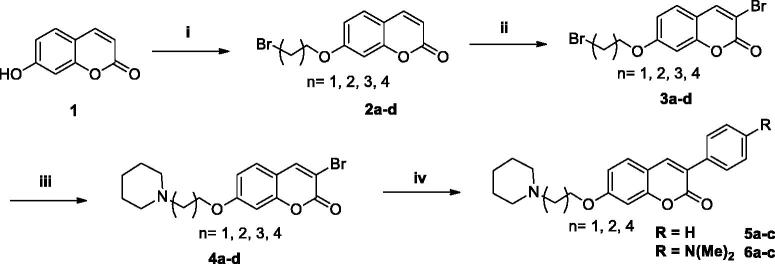
Reagents and conditions: (i) Br(CH_2_)_n_Br (*n* = 2–5), K_2_CO_3_, acetone, 60° C, 2-8h, 68–78%; (ii) Br_2_, AcOH, NaOAc, r.t., 2h, 79–84%; (iii) piperidine, acetonitrile, 60° C, 2-5h, 95–99%; (iv) Ph-B(OH)_2_ or 4-(Me)_2_N-Ph-B(OH)_2_, Na_2_CO_3_, Pd(PPh_3_)_4_, H_2_O, EtOH, PhMe, 80° C, 3h, 70–75%.

The synthesis of the desired compounds started with the 7-OH-coumarin (**1**). In the first step, through an *O*-alkylation reaction of **1** with diverse dibromo-alkanes, the 7-bromoalkoxy-coumarin products (**2a–d**) were obtained in yields of 68–78%. The second step consisted of a bromination reaction of the bromoalkoxy-coumarins (**2a–d**) using Br_2_ in buffered medium of sodium acetate/acetic acid at room temperature, furnishing the brominated derivatives (**3a–d**) in yields ranging between 79–84%. These intermediates (**3a–d**) were then subjected to amination reactions with piperidine in acetonitrile, leading to the formation of the desired 7-amino-alkoxy-3-bromo-coumarin derivatives (**4a–d**) as yellow solids in 95–99% yields after purification by flash chromatography. From the 7-amino-alkoxy-3-bromo-coumarin derivatives with 2, 3 and 5 methylene spacers (**4a, 4b, 4d**), Suzuki cross coupling reactions were then carried out using Pd(PPh_3_)_4_ catalyst and phenyl and 4-dimethylamino-phenyl boronic acids to obtain the final arylated 3-substituted coumarins (**5a–c** and **6a–c**) in yields ranging between 70–75% after purification by flash chromatography (Scheme 2).

### Cholinesterase inhibitory activity, biological profile, and SAR analyses

The inhibitory activities of the coumarin compounds (**4a–d**, **5a–c** and **6a–c**) on AChE and BuChE were determined by the Ellman’s method^14^ ^,^ ^15^ using donepezil as the reference compound. As depicted in Table 1, compounds presented potent inhibitory activities against AChE with IC_50_ values varying from 0.02 to 0.92 µM for compounds **6a** and **6c** respectively. On the other hand, the tested coumarins were not so efficient in inhibiting BuChE with IC_50_ ranging from 0.90 to 15.87 µM, demonstrating a good selectivity for AChE. The inhibition behavior of the simplest bromo-coumarins (**4a–d**) was quite similar of that related in the literature for AChE^13^, the bigger the methylene chain the lower the activity. However, we were surprised by compound **4d** with a five-methylene spacer link that was equipotent to **4a** (IC_50_=0.14 µM for **4d** and IC_50_=0.18 µM for **4a**). Conversely, the inhibitions of BuChE was in general inverse to those of AChE, and compounds with longest linker chains were more potent in inhibiting BuChE (IC_50_=8.37 µM for **4a** and IC_50_=5.00 µM for **4d**). By this way, we decided to evaluate the 3-aryl substituted coumarins with 2, 3 and 5 methylene spacers in the 7-amino-alkoxy group. In general, the substitution of bromine for phenyl (**5a–c**) or 4-dimethylamine-phenyl (**6a–c**) led to compounds with better potencies on the inhibition of both AChE and BuChE, and a reduction in the selectivity index (IC_50_ BuChE/AChE). However, one compound behavior itself differently and presented an interesting profile, the 4-dimethylamine-phenyl substituted coumarin (**6a**) with the best inhibition of AChE (IC_50_=0.02 µM) and selectivity (IC_50_ BuChE/AChE = 354), quite similar to the reference drug donepezil (IC_50_ AChE = 0.007 µM and selectivity = 365) (Table 1).

**Table 1.** AChE and BuChE inhibitory activities of coumarin compounds.


			IC_50_(μM)±SD^a^			
Compound	R	n	AChE^b^	BuChE^c^	SI^d^	FRAP value (mmol Q/mol)^e^
**4a**	Br	1	0.18 ± 0.009	8.37 ± 0.167	47	NA
**4b**	Br	2	0.37 ± 0.008	15.87 ± 0.007	42	NA
**4c**	Br	3	0.55 ± 0.010	4.92 ± 0.095	9	NA
**4d**	Br	4	0.15 ± 0.005	5.01 ± 0.253	33	NA
**5a**	Ph	1	0.14 ± 0.009	2.50 ± 0.177	18	NA
**5b**	Ph	2	0.24 ± 0.014	1.86 ± 0.024	8	NA
**5c**	Ph	4	0.45 ± 0.036	0.90 ± 0.001	2	NA
**6a**	4-(CH_3_)_2_N-Ph	1	0.02 ± 0.001	6.73 ± 0.040	354	7.49 ± 0.61
**6b**	4-(CH_3_)_2_N-Ph	2	0.33 ± 0.011	7.27 ± 0.273	22	2.42 ± 0.19
**6c**	4-(CH_3_)_2_N-Ph	4	0.96 ± 0.036	3.85 ± 0.190	4	2.77 ± 0.00
**Donepezil**	–	–	0.007 ± 0.0002	2.39 ± 0.105	365	–

^a^Concentration required for 50% inhibition of ChEs, data were shown in mean ± SD of triplicate independent experiments; ^b^AChE from electric eel; ^c^BuChE from horse serum; ^d^Selectivity index (SI) is defined as BuChE IC_50_/AChE IC_50_. ^e^Antioxidant index based on quercetine (Q); FRAP value (mmol Q/mol). 7,8-dimethoxy-coumarin (NA)^25^ and ethyl 2–(7,8-dimethoxy-2-oxo-2H-chromen-3-yl)acetate (1.2 ± 0.1)^25^.

The antioxidant evaluation of coumarin compounds showed that only **6a–c** presented activity in Ferric Ion Reduction Method (FRAP) with values from 2.42 to 7.49 mmol Q/mol (Table 1). Series **4a–d** and **5a–c** did not demonstrate any considerable result, similar to other 7-alkoxy coumarins described in the literature^25^. Probably, the antioxidant effect is coming from dimethylamino-phenyl moiety and this feature could be explored in a forthcoming series.

**Figure 1.**
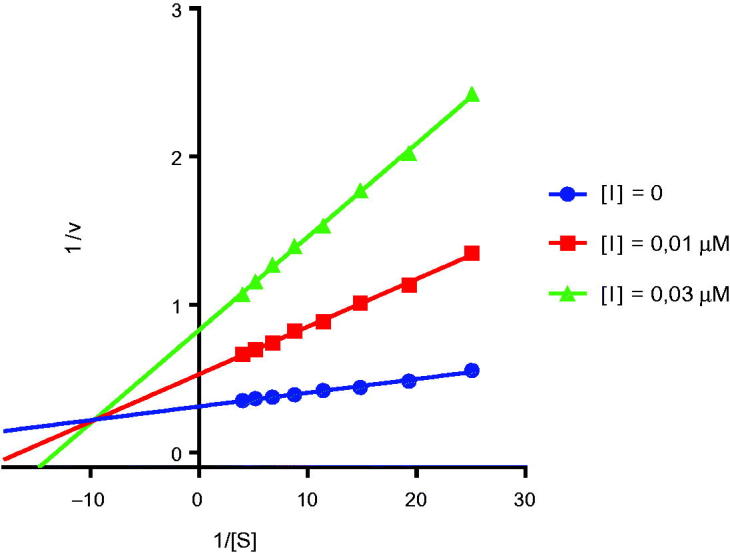
Lineweaver-Burk plots of EeAChE inhibition kinetics of compound **6a**. Inset: concentrations used for **6a** are depicted with [I] graphic symbol.

Aiming at discovery the mode of action of coumarins described herein, the most potent compounds from the bromo and aryl 3-substituted coumarins, i.e. **4d** and **6a**, were selected for kinetic studies. The linear Lineweaver–Burk equation of the Michaelis–Menten was applied to evaluate the inhibition profile. Increasing concentrations of both compounds were able to increase K_m_ and decrease V_max_, presenting a mixed-type inhibition in AChE as well as in BuChE, as exemplified in Figure 1 for compound **6a** (complete analysis in Supplementary material). The competitive inhibitory constant (Ki) and the noncompetitive constant (Ki’) for **6a** and **4d** are described in Table 3 at Supplementary material. As example, the best Ki values against AChE were obtained for compound **6a**: Ki = 0.001 µM (competitive) and Ki’=0.010 µM (noncompetitive).

**Figure 2.**
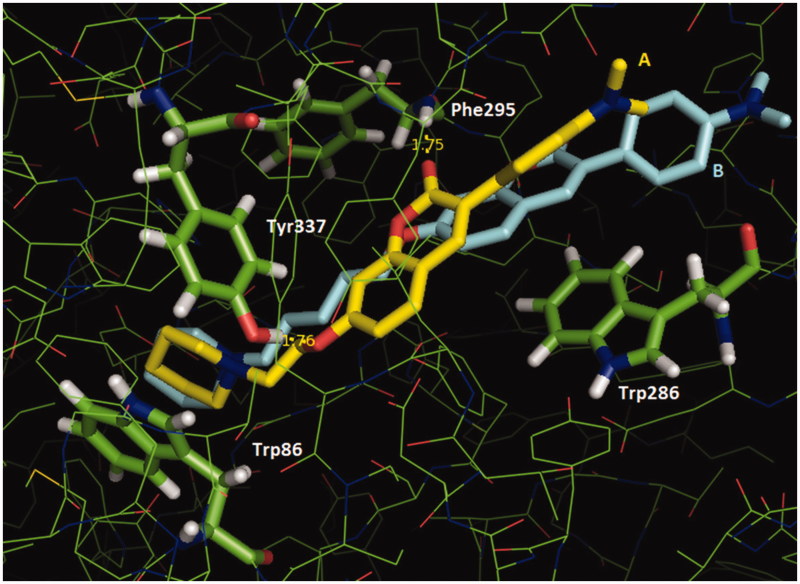
Superposition of the interaction poses of compounds **6a** (A, carbon atoms in yellow) and **6c** (B, carbon atoms cyan) with EeAChE obtained by molecular docking (Goldscore function). H-bond distances (Å) are shown in yellow. Figure generated with PyMol 0.99 (DeLano Scientific LLC).

With the complete inhibitory profile of the target compounds, we proceeded with a molecular modeling evaluation to understand the importance of changing the methylene size spacer and nature of substituents in position 3 of coumarins. Thus, we selected compounds **4a** and **6a**, the strongest 2-methylene spacer bromo and aryl substituted coumarins inhibitors of AChE; and **6c**, the weakest inhibitor. Docking results in EeAChE and EqBuChE are presented in Table 4 at Supplementary material. All inhibitors were generally predicted as better ligands of EeAChE, being **6a** (Goldscore = 78.1) better than **6c** (Goldscore = 71.4) and **4a** (Goldscore = 64.9), whereas **6c** (Goldscore = 67.9) was better than **6a** (Goldscore = 63.0) and **4a** (Goldscore = 57.6) as a ligand of EqBuChE, in qualitative accordance to our experimental results. The molecular docking results of compound **4a**, **6a** and **6c** showed that all were able to occupy the peripheral (PAS) and the catalytic (CAS) sites simultaneously in the EeAChE (Figure 2) (and Figure 5 at Supplementary material), as previewed by kinetic evaluations. In the CAS, they interact similarly by means of their protonated piperidinyl group with Trp86 (a cation-π interaction). In the PAS, both **6a** and **6c** molecules were involved in π-stacking interactions with Trp286, which was more effective for **6c**, involving its coumarin ring (Figure 2). On the other hand, **4a** was only capable of doing weak hydrophobic interactions with Trp286 (Figure 5 at Supplementary material). The presence of a narrower spacer in **6a** makes its coumarin ring to be best located in the gorge, where it is involved in H-bonds with Tyr337 and the peptide group of Phe295. These H-bonds, that had no counterparts in the **6c**/enzyme complex, were probably the reason for the most effective interaction between compounds with short spacers and EeAChE, which could be related to their greater inhibitory action over the enzyme.

### Cell cytotoxicity and in silico ADMET physico-chemical profile analysis

**Figure 3.**
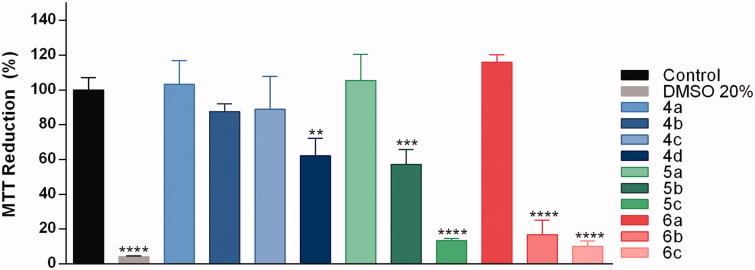
Neuroblastoma cell viability after compound treatment. Samples containing compounds were added to the culture 48 h before MTT addition. The compounds were tested at the final concentration of 50 µM. MTT reduction was evaluated as described in Experimental Procedures. Data are expressed as the percentage of MTT reduction relative to the value for control cells (cells without treatment). Error bars represent standard deviations. **p < .01; ***p < .001; ****p < .0001.

In order to accede the drugability of tested coumarins, we first proceeded with the cytotoxicity evaluation against N2a cells (neuroblastoma), after 48 h incubation at concentrations of 10 and 50 µM (Figure 3 and Supplementary material). The most potent compounds in inhibiting AChE, i.e. **4a**, **5a**, and **6a**, were not cytotoxic at the maximum tested concentration (50 µM) (Figure 3). As a rule, long methylene chains (three or five spacers) in phenyl-substituted coumarins (**5b**, **5c**, **6b** and **6c**) could not be useful for further developments due to increase in toxicity.

**Figure 4.**
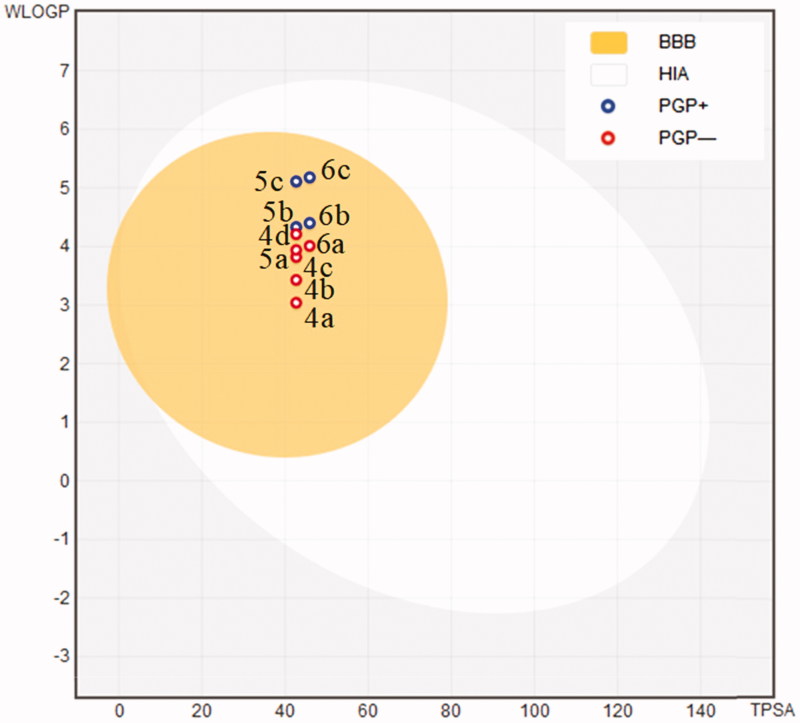
BOILED-Egg ADMET model^27^ for coumarin compounds **4a–d**, **5a–c,** and **6a–c**. (HIA) gastrointestinal absorption; (BBB) brain penetration; (PGP+) substrate for P‐glycoprotein; (PGP-) Not a substrate for P‐glycoprotein.

Finally, in silico evaluations showed a good ADMET profile for coumarin compounds. Parameters as topological polar surface area (TPSA), consensus Log P, Log S, human intestinal absorption (HIA), blood–brain barrier permeation (BBB), and P‐glycoprotein (P-gP) substrate and drug-likeness profile (Supplementary material)^26^. TPSA values and consensus Log P ranged from 42.68 to 45.92 and 3.35 to 5.01, respectively. The moderate polarity (PSA < 79 Å^2^) and relative lipophilicic characteristics put our compounds in the yellow compartment of BOILED‐Egg model (Figure 4), having a high probability to access the CNS^27^, which is fundamental for the distribution of central‐acting molecules. Additionally, the most potent compounds **4a**, **5a** and **6a** were not considered as P-gP substrate and having a good drug-likeness profile with no one violation on the Lipinski^28^, Ghose^29^, Veber^30^, Egan^31^ and Muegge^32^ rules.

## Conclusions

The designed and synthesized coumarin compounds were able to potently inhibit cholinesterases in the nanomolar range. In general, compounds with narrow methylene linkers were more potent and selective for AChE (with IC_50_ and selectivity of up to 20 nM and 354 times, respectively), and less toxic as well. The introduction of aromatic substituents in position 3 of coumarins led to compounds with better potencies on the inhibition of both AChE and BuChE. As highlighted, compound **6a** could be elected as a hit for *in vivo* studies, showing good AChE inhibition potency and selectivity (IC_50_=20 nM and 354 times), antioxidant properties, low cytotoxicity and good predict ADMET profile.

## Supplementary Material

Supplemental Material
