# Supplementary material for: Discovery of novel dual-active 3-(4-(dimethylamino)phenyl)-7-aminoalcoxy-coumarin as potent and selective acetylcholinesterase inhibitor and antioxidant
Source: J Enzyme Inhib Med Chem. 2019 Feb 6;34(1):631–7. doi: 10.1080/14756366.2019.1571270 (PMC6366430; doi:10.1080/14756366.2019.1571270)

Discovery of Novel Dual-active 3-(4-(dimethylamino)phenyl)-7-aminoalcoxy-coumarin as Potent and Selective Acetylcholinesterase Inhibitor and Antioxidant.

Gabriela Alves de Souza[a,b], Soraia John da Silva[a], Catarina de Nigris Del Cistia[a], Paulo Pitasse-Santos[a], Lucas de Oliveira Pires[a], Yulli Moraes Passos[c], Yraima Cordeiro[c], Cristiane Martins Cardoso[a], Rosane Nora Castro[a], Carlos Mauricio R. Sant'Anna[a], and Arthur Eugen Kümmerle[a]*

[a] *Programa de Pós-Gradução em Química (PPGQ), Universidade Federal Rural do Rio de Janeiro, Seropédica, Rio de Janeiro, 239897-000, Brazil.*

[b] *Laboratório de Diversidade Molecular e Química Medicinal (LaDMol-QM, Molecular Diversity and Medicinal Chemistry Laboratory), Departament of Chemistry, Universidade Federal Rural do Rio de Janeiro, Seropédica, Rio de Janeiro, 239897-000, Brazil.*

[c] *Faculdade de Farmácia, Universidade Federal do Rio de Janeiro, Rio de Janeiro, Rio de Janeiro, 21941-599, Brazil.*

** akummerle@hotmail.com*

Supporting Information

Contents

General Procedures ……………………….……………………………………2-12

Biological assessment results………………………………………………….13-17

References ……………………………………………………………………..…28

Copies of ^{1}H , ^{13}C NMR and Mass Spectra ………………..………………………29-51

Materials

All chemical reagents were purchased from Sigma-Aldrich (St. Louis, MO) unless otherwise stated. Reactions were monitored by thin-layer chromatography (TLC) on 0.25 mm Merck (Darmstadt, Germany) silica gel plates (60F-254) and visualized under UV lamp (254 and 365 nm). All melting points (mp) were measured using a Melting Point AAKER model PFM-II and were uncorrected. ^{1}H NMR and ^{13}C NMR spectra were measured on a BRUKER Ultrashield Plus spectrometer (Billerica, MA) at 25 °C and referenced to TMS. Chemical shifts are reported in ppm (δ) using the residual solvent line as internal standard. Splitting patterns are designed as s, singlet; d, doublet; t, triplet; m, multiplet; brs, broad singlet. The HRMS analysis was performed using a QExactive™ Hybrid Quadrupole Orbitrap Mass Spectrometer (Thermo Fisher Scientific, Waltham, USA) using electrospray ionization (ESI). Standard working solutions of compounds **4a-d**, **5a-c** and **6a-c** (1000 ng·mL-1) were prepared with water/methanol 7:3 and fortified with 0.1% formic acid and 5 mM NH_4COOH (ammonium formate), and the solutions were used by direct infusion.

General procedures of synthesis and spectral data

7-bromoalkoxy-coumarins (2a-d)

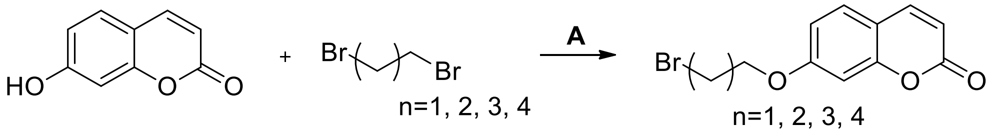

1 **7a-d** **2a-d**

A –In a 25 mL borosilicate reaction tube were added 2 mL of acetone, 10.0 – 15.0 mmol (4-6 eq.) of dibromoalkane **(7a–d)**, as shown in the **Table 1**, and 5.0 mmol (2 eq.) of potassium carbonate (K_2CO_3). Thereafter, a solution of 2.5 mmol of 7-hydroxycoumarin **(1)** in 8 mL of acetone (CH_3COCH_3), previously prepared in a 25 mL beaker, was added dropwise to the reaction tube under stirring. Following, the reaction tube was sealed and the reaction kept at 60°C for 6 – 12h, being monitored by TLC. At the end of the reaction (complete consumption of **1**) the solvent was removed in a rotary evaporator, the product diluted in 30 mL water and then extracted with 3 × 15 mL ethyl acetate. The combined organic phases were dried over sodium sulfate (Na_2SO_4), filtered and the solvent removed in a rotary evaporator. The products were collected as oils which were precipitated in presence of 50 mL hexanes and irradiation in an ultrasonic

bath. After filtration, the solids were dried at room temperature yielding the 7-bromoalkoxy-coumarin derivatives **2a-d**.

Table 1. Amount of dibromoalkanes **(7a-d)** used for the reactions

Compound	mmol
**7a**	15.0
**7b**	12.5
**7c**	10.0
**7d**	10.0

7-(2-bromoethoxy)-2H-chromen-2-one (2a)

Light yellow solid, 75% yield, mp: 132°C. ^{1}H NMR (500 MHz, $CDCl_3$): δ 7.65 (d, J = 9.3 Hz, 1H); 7.40 (d, J = 8.5 Hz, 1H); 6.88 (dd, J = 8.4, 2.1 Hz, 1H); 6.82 (d, J = 1.8 Hz, 1H); 6.28 (d, J = 9.5 Hz, 1H); 4.36 (t, J = 6.1 Hz, 2H); 3.68 (t, J = 6.0 Hz, 2H). ^{13}C NMR (500 MHz, $CDCl_3$): δ 161.14; 161.04; 155.73; 143.29; 128.93; 113.53; 113.02; 112.83; 101.63; 68.15; 28.41.

7-(3-bromopropoxy)-2H-chromen-2-one (2b)

Light yellow solid, 78% yield, mp: 101°C. ^{1}H NMR (500 MHz, $CDCl_3$): δ 7.64 (d, J = 9.5 Hz, 1H); 7.38 (d, J = 8.5 Hz, 1H); 6.82-6.86 (m, 2H); 6.26 (d, J = 9.3 Hz, 1H); 4.17 (t, J = 5.9 Hz, 2H); 3.61 (t, J = 6.3 Hz, 2H); 2.36 (quint, J = 6.1 Hz, 2H). ^{13}C NMR (500 MHz, $CDCl_3$): δ 161.79; 161.11; 155.77; 143.33; 128.79; 118.52; 113.19; 112.69; 101.47; 65.80; 31.89; 29.54.

7-(4-bromobutoxy)-2H-chromen-2-one (2c)

Light yellow solid, 70% yield, mp: 65°C. ^{1}H NMR (500 MHz, $CDCl_3$): δ 7.64 (d, J = 9.5 Hz, 1H); 7.37 (d, J = 8.8 Hz, 1H); 6.83 (dd, J = 8.5, 2.3 Hz, 1H); 6.80 (d, J = 2.3 Hz, 1H); 6.25 (d, J = 9.3 Hz, 1H); 4.06 (t, J = 5.9 Hz, 2H); 3.50 (t, J = 6.4 Hz, 2H); 2.05-2.12 (m, 2H); 1.96-2.02 (m, 2H). ^{13}C NMR (500 MHz, $CDCl_3$): δ 162.01; 161.17; 155.82; 143.38; 128.74; 113.07; 112.82; 112.52; 101.28; 67.48; 33.19; 29.25; 27.59.

7-(5-bromopentoxy)-2H-chromen-2-one (2d)

Light orange oil, yield 68%. ^{1}H NMR (500 MHz, $CDCl_3$): δ 7.64 (d, J = 9.5 Hz, 1H); 7.37 (d, J = 8.5 Hz, 1H); 6.83 (dd, J = 8.7, 2.1 Hz, 1H); 6.80 (brs, 1H); 6.25 (d, J = 9.5

Hz, 1H); 4.03 (t, J = 6.1 Hz, 2H); 3.45 (t, J = 6.7 Hz, 2H); 1.92-1.99 (m, 2H); 1.82-1.89 (m, 2H); 1.61-1.69 (m, 2H). ^{13}C NMR (500 MHz, $CDCl_3$): δ 162.16; 161.21; 155.84; 143.41; 128.71; 112.97; 112.88; 112.43; 101.26; 68.16; 33.45; 32.32; 28.12; 24.68.

3-bromo-7-bromoalkoxy-coumarins (3a-d)

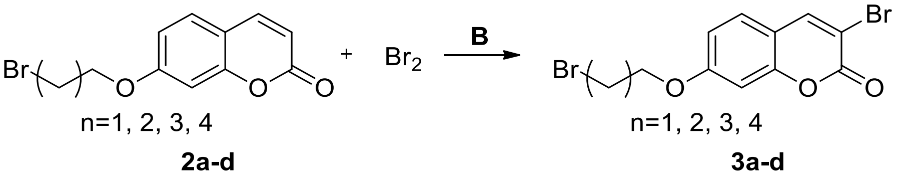

B – In a 25 mL borosilicate reaction tube were added 1.7 mmol of the appropriate 7-bromoalkoxy-coumarin derivative **(2a–d)**, 2.1 mmol (1.3 eq.) of bromine (Br_2), 5.0 mmol (3 eq.) of sodium acetate and 8 mL of glacial acetic acid. The reaction was kept under stirring at room temperature for 2h. After complete consumption of **2a**–**d**, monitored by TLC, the reaction was added to a 50 mL beaker containing crushed ice. The precipitated was then vacuum filtered and dried at room temperature. Products were purified using an Isolera equipment (Biotage, model ISO-4SV) with a 10 g silica gel cartridge eluted with a mixture of hexanes: dichloromethane in a concentration gradient ranging from 50 – 90% of the most polar solvent.

3-bromo-7-(2-bromoethoxy)-2H-chromen-2-one (3a)

White solid, 95% yield, mp: 183°C. ^{1}H NMR (500 MHz, DMSO-d_6): δ 8.55 (s, 1H); 7.63 (d, J = 8.8 Hz, 1H); 7.08 (d, J = 2.3 Hz, 1H); 7.02 (dd, J = 8.8, 2.5 Hz, 1H); 4.44 (t, J = 6.0 Hz, 2H); 3.84 (t, J = 6.1 Hz, 2H). ^{13}C NMR (500 MHz, DMSO-d_6): δ 161.24; 156.81; 154.59; 145.28; 129.11; 113.39; 113.36; 106.91; 101.34; 68.50; 31.07.

3-bromo-7-(3-bromopropoxy)-2H-chromen-2-one (3b)

White solid, 97% yield, mp: 120°C. ^{1}H NMR (500 MHz, $CDCl_3$): δ 8.02 (s, 1H); 7.36 (d, J = 8.8 Hz, 1H); 6.88 (dd, J = 8.7, 2.4 Hz, 1H); 6.83 (d, J = 2.3 Hz, 1H); 4.18 (t, J = 5.8 Hz, 2H); 3.61 (t, J = 6.3 Hz, 2H); 2.36 (quint, J = 6.1 Hz, 2H). ^{13}C NMR (500 MHz, $CDCl_3$): δ 162.01; 157.39; 154.95; 144.36; 128.13; 113.34; 113.19; 107.85; 101.38; 65.95; 31.84; 29.47.

3-bromo-7-(4-bromobutoxy)-2H-chromen-2-one (3c)

White solid, 98% yield, mp: 120°C. ^{1}H NMR (500 MHz, $CDCl_3$): *δ* 8.02 (s, 1H); 7.35 (d, *J* = 8.8 Hz, 1H); 6.86 (dd, *J* = 8.7, 2.4 Hz, 1H); 6.80 (d, *J* = 2.3 Hz, 1H); 4.06 (t, *J* = 5.9 Hz, 2H); 3.50 (t, *J* = 6.4 Hz, 2H); 2.05-2.12 (m, 2H); 1.96-2.03 (m, 2H). ^{13}C NMR (500 MHz, $CDCl_3$): *δ* 162.22; 157.45; 155.00; 144.41; 128.08; 113.47; 113.02; 107.70 101.17; 67.61; 33.15; 29.21; 27.56.

3-bromo-7-(5-bromopentoxy)-2H-chromen-2-one (3d)

White solid, 99% yield, mp: 123°C. ^{1}H NMR (500 MHz, $CDCl_3$): *δ* 8.02 (s, 1H); 7.35 (d, *J* = 8.8 Hz, 1H); 6.86 (dd, *J* = 8.7, 2.4 Hz, 1H); 6.80 (d, *J* = 2.3 Hz, 1H); 4.03 (t, *J* = 6.3 Hz, 2H); 3.45 (t, *J* = 6.8 Hz, 2H); 1.92-1.99 (m, 2H); 1.83-1.90 (m, 2H); 1.61-1.69 (m, 2H). ^{13}C NMR (500 MHz, $CDCl_3$): *δ* 162.38; 157.48; 155.03; 144.44; 128.06; 113.53; 112.95; 107.60 101.15; 68.32; 33.45; 32.30; 28.09; 24.68.

3-bromo-7-aminoalkoxy-coumarins (4a-d)

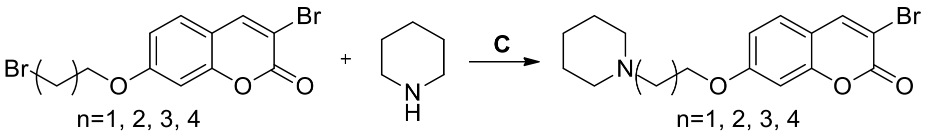

3a-d **8** **4a-d**

C – In a 25 mL borosilicate reaction tube were added 1.3 mmol of the appropriate 3-bromo-7-bromoalkoxycoumarin derivative **(3a–d)**, 3.9 mmol (3 eq.) of piperidine **(8)** and 8 mL of acetonitrile. The tube was then sealed and the reaction kept under stirring at 60°C for 3 – 8 h. After complete consumption of the **3a**–**d**, monitored by TLC, acetonitrile was removed in a rotary evaporator and the products were purified using an Isolera equipment (Biotage, model ISO-4SV) with a 10 g silica gel cartridge eluted with a mixture of dichloromethane: methanol in a concentration gradient ranging from 0 – 25% of the more polar solvent.

3-bromo-7-[2-(piperidin-1-yl)ethoxy]-2H-chromen-2-one (4a)

Light orange solid, 95% yield, mp: 215°C. ^{1}H NMR (500 MHz, DMSO-d_6): *δ* 8.57 (s, 1H); 7.64 (d, *J* = 8.5 Hz, 1H); 7.10 (brs, 1H); 7.02 (dd, *J* = 8.7, 1.7 Hz, 1H); 4.34 (brs, 2H); 2.87-3.16 (m, 6H); 1.64 (brs, 4H); 1.46 (brs, 2H). ^{13}C NMR (500 MHz, DMSO-d_6): *δ* 161.31; 156.78; 154.56; 145.27; 129.03; 113.42; 113.26; 106.81; 101.34; 53.45; 43.69; 22.18; 21.61. HRMS (ESI) *m/z* calculated for $C_{16}H_{19}BrNO_3$ $[M + H]^+$ 552.0548 was found to be 552.05390.

3-bromo-7-[3-(piperidin-1-yl)propoxy]-2H-chromen-2-one (4b)

Light orange solid, 97% yield, mp: 242°C. ^{1}H NMR (500 MHz, $CDCl_3$): δ 8.01 (s, 1H); 7.34 (d, J = 8.5 Hz, 1H); 6.88 (dd, J = 8.8, 2.2 Hz, 1H); 6.83 (d, J = 2.2 Hz, 1H); 4.21 (t, J = 5.7 Hz, 2H); 3.17 (t, J = 5.4 Hz, 2H); 2.87 (t, J = 5.7 Hz, 2H); 1.92-1.94 (m, 2H); 1.63-1.70 (m, 6H); 1.47-1.48 (m, 2H). ^{13}C NMR (500 MHz, DMSO-d_6): δ 161.56; 156.79; 154.59; 145.30; 129.06; 113.32; 113.17; 106.74; 101.16; 65.84; 53.22; 52.18; 23.20; 22.55; 21.27. HRMS (ESI) *m/z* calculated for $C_{17}H_{21}BrNO_3$ $[M+H]^+$ 366.0705 was found to be 366.07018.

3-bromo-7-[4-(piperidin-1-yl)butoxy]-2H-chromen-2-one (4c)

White solid, 98% yield, mp: 225°C. ^{1}H NMR (500 MHz, $CDCl_3$/ CD_3OD): δ 7.97 (s, 1H); 7.29 (m, 1H); 6.78 (d, J = 10.1 Hz, 1H); 6.70 (brs, 1H); 3.97 (t, J = 5.8 Hz, 2H); 3.47-3.48 (m, 2H); 2.99-3.02 (m, 2H); 2.70 (brs, 2H); 1.93-1.99 (m, 4H); 1.78-1.81 (m, 4H). ^{13}C NMR (500 MHz, $CDCl_3$/ CD_3OD): δ 161.95; 157.75; 154.64; 144.69; 128.18; 113.21; 113.03; 107.16; 101.12; 67.38; 56.64; 52.99; 25.94; 22.43; 21.51; 20.51. HRMS (ESI) *m/z* calculated for $C_{18}H_{23}BrNO_3$ $[M+H]^+$ 380.0861 was found to be 380.08518.

3-bromo-7-[5-(piperidin-1-yl)pentoxy]-2H-chromen-2-one (4d)

White solid, 99% yield, mp: 176°C. ^{1}H NMR (500 MHz, DMSO-d_6): δ 9.11 (brs, 1H); 8.56 (s, 1H); 7.62 (d, J = 8.8 Hz, 1H); 7.04 (d, J = 1.9 Hz, 1H); 6.98 (d, J = 8.5, 2.2 Hz, 1H); 4.10 (t, J = 6.3 Hz, 2H); 3.32 (brs, 2H); 3.02-3.05 (m, 2H); 2.85 (brs, 2H); 1.69-1.80 (m, 10H); 1.41-1.47 (m, 2H). ^{13}C NMR (500 MHz, DMSO-d_6): δ 162.02; 156.83; 154.66; 145.35; 129.02; 113.32; 112.93; 106.47; 101.02; 68.06; 55.64; 52.00; 27.83; 22.86; 22.62; 22.52; 21.35. HRMS (ESI) *m/z* calculated for $C_{19}H_{25}BrNO_3$ $[M+H]^+$ 394.1018 was found to be 394.10079.

3-phenyl-7-aminoalkoxy-coumarins (5a-5c and 6a-6c)

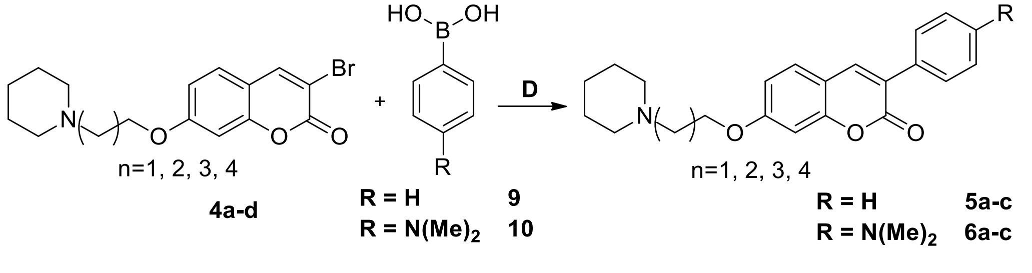

D – In a 10 mL borosilicate reaction tube were added 0.14 mmol of the appropriate 3-bromine-7-aminoalkoxy-coumarin derivative **(4a;b;d)**, 0.20 mmol (1.4 eq.) of properly

substituted phenylboronic acid **(9-10)** and 0.41 mmol (3 eq.) of potassium carbonate in 4 ml of a solvent mixture (water: ethanol: toluene (2: 1: 1)). The reaction was then degassed with nitrogen gas for 10 minutes. Following, 0.01 mmol (7 mol%) of $Pd(PPh_3)_4$ (catalyst) was added under nitrogen flux, the reaction tube sealed and the reaction kept under stirring and heating at 65°C for 3 – 5 h. After complete consumption of **4a;b;d**, monitored by TLC, the solvent was removed in a rotary evaporator. The products were purified using an Isolera equipment (Biotage, model ISO-4SV) with a 10 g silica gel cartridge eluted with a mixture of dichloromethane: methanol in a concentration gradient ranging from 0 – 25% of the more polar solvent.

3-phenyl-7-[2-(piperidin-1-yl)ethoxy]-2H-chromen-2-one (5a)

Ligth yellow solid, 75% yield, mp: 90°C. ^{1}H NMR (500 MHz, DMSO-d_6): δ 8.20 (s, 1H); 7.67-7.71 (m, 3H); 7.45 (t, J = 7.4 Hz, 2H); 7.39 (t, J = 7.4 Hz, 1H); 7.04 (brs, 1H); 6.98 (dd, J = 8.5 Hz, 1.9 Hz, 1H); 4.18 (t, J = 5.7 Hz, 2H); 2.68 (t, J = 5.7 Hz, 2H); 2.43 (brs, 4H); 1.49-1.51 (m, 4H); 1.37-1.38 (m, 2H). ^{13}C NMR (500 MHz, DMSO-d_6): δ 161.65; 159.99; 154.81; 140.87; 134.95; 129.69; 128.32; 128.20; 128.18; 123.14; 113.08; 113.02; 100.76; 66.38; 57.15; 54.37; 25.55; 23.91. HRMS (ESI) *m/z* calculated for $C_{22}H_{24}NO_3$ $[M + H]^+$ 350.1756 was found to be 350.17459.

3-phenyl-7-[3-(piperidin-1-yl)propoxy]-2H-chromen-2-one (5b)

Ligth yellow solid, 75% yield, mp: 114°C. ^{1}H NMR (500 MHz, $CDCl_3$): δ 7.76 (s, 1H); 7.69-7.70 (m, 2H); 7.39-7.46 (m, 4H); 6.86-6.87 (m, 2H); 4.09 (brs, 2H); 2.47-2.55 (m, 6H); 2.05-2.06 (m, 2H); 1.64 (brs, 4H); 1.47 (brs, 2H). ^{13}C NMR (500 MHz, $CDCl_3$): δ 162.04; 160.92; 155.27; 140.05; 135.03; 128.78; 128.39; 128.37; 124.67; 113.23; 113.07; 100.99; 67.04; 55.63; 54.53; 26.37; 25.73; 24.25. HRMS (ESI) *m/z* calculated for $C_{23}H_{26}NO_3$ $[M + H]^+$ 364.1913 was found to be 364.19085.

3-phenyl-7-[5-(piperidin-1-yl)pentoxy]-2H-chromen-2-one (5c)

Ligth yellow solid, 80% yield, mp: 198°C. ^{1}H NMR (500 MHz, $CDCl_3$/ CD_3OD): δ 7.44 (s, 1H); 7.28 (m, 1H); 7.00-7.10 (m, 5H); 6.47-6.51 (m, 2H); 3.68 (t, J = 5.5 Hz, 2H); 2.41-2.52 (m, 6H); 1.43-1.48 (m, 8H); 1.15-1.22 (m, 4H). ^{13}C NMR (500 MHz, $CDCl_3$/ CD_3OD): δ 161.86; 161.36; 154.89; 140.44; 134.63; 128.81; 128.13; 128.10; 124.27; 113.10; 112.96; 100.63; 67.87; 57.59; 53.35; 28.27; 24.03; 23.30; 22.31. HRMS (ESI) *m/z* calculated for $C_{25}H_{30}NO_3$ $[M + H]^+$ 392.2226 was found to be 392.22138.

3-[4-(dimethylamino)phenyl]-7-[2-(piperidin-1-yl)ethoxy]-2H-chromen-2-one (6a)

Green solid, 70% yield, mp: 140°C. ^{1}H NMR (500 MHz, $CDCl_3$): δ 7.67 (s, 1H); 7.63 (d, J = 8.8 Hz, 2H); 7.40 (d, J = 8.5 Hz, 1H); 6.84-6.87 (m, 2H); 6.77 (d, J = 8.8 Hz, 2H); 4.20 (t, J = 5.8 Hz, 2H); 3.01 (s, 6H); 2.86 (t, J = 5.7 Hz, 2H); 2.59 (brs, 4H); 1.66 (quin, J = 5.5 Hz, 4H); 1.48 (brs, 2H). ^{13}C NMR (500 MHz, $CDCl_3$): δ 161.58; 161.17; 154.86; 150.78; 137.33; 129.41; 128.57; 125.15; 122.90; 114.17; 113.07; 112.27; 101.32; 66.47; 57.75; 55.22; 40.63; 25.84; 24.17. HRMS (ESI) *m/z* calculated for $C_{24}H_{29}N_2O_3$ $[M+H]^+$ 393.2178 was found to be 393.21741.

3-[4-(dimethylamino)phenyl]-7-[3-(piperidin-1-yl)propoxy]-2H-chromen-2-one (6b)

Yellow solid, 75% yield, mp: 153°C. ^{1}H NMR (500 MHz, $CDCl_3$): δ 7.67 (s, 1H); 7.63 (d, J = 8.8 Hz, 2H); 7.39 (d, J = 9.1 Hz, 1H); 6.83-6.85 (m, 2H); 6.78 (d, J = 8.8 Hz, 2H); 4.08 (t, J = 6.1 Hz, 2H); 3.01 (s, 6H); 2.49-2.58 (m, 6H); 2.04-2.1 (m, 2H); 1.65-1.67 (m, 4H); 1.48 (brs, 2H). ^{13}C NMR (500 MHz, $CDCl_3$): δ 161.35; 161.26; 154.58; 150.52; 137.13; 129.15; 128.29; 124.78; 122.70; 113.76; 112.76; 112.03; 100.95; 66.90; 55.72; 54.55; 40.39 26.31; 25.61; 24.17. HRMS (ESI) *m/z* calculated for $C_{25}H_{31}N_2O_3$ $[M+H]^+$ 407.2335 was found to be 407.23246.

3-[4-(dimethylamino)phenyl]-7-[5-(piperidin-1-yl)pentoxy]-2H-chromen-2-one (6c)

Yellow solid, 77% yield, mp: 206°C. ^{1}H NMR (500 MHz, $CDCl_3$): δ 7.67 (s, 1H); 7.63 (d, J = 8.5 Hz, 2H); 7.40 (d, J = 8.5 Hz, 1H); 6.81-6.84 (m, 2H); 6.77 (d, J = 8.8 Hz, 2H); 4.02 (t, J = 6.3 Hz, 2H); 3.00 (s, 6H); 2.61-2.72 (m, 6H); 1.79-1.89 (m, 8H); 1.50-1.56 (m, 4H). ^{13}C NMR (500 MHz, $CDCl_3$): δ 161.34; 161.23; 154.65; 150.51; 137.14; 129.14; 128.34; 124.75; 122.66; 113.74; 112.71; 112.01; 100.88; 68.09; 58.38; 53.96; 40.38; 28.68; 25.07; 24.30; 23.85; 23.32. HRMS (ESI) *m/z* calculated for $C_{27}H_{35}N_2O_3$ $[M+H]^+$ 435.2648 was found to be 435.26382.

Biological assessment

Anticholinesterase activity assays

The anticholinesterase activity was determined according to adapted Ellman's method[1]. All of the solution were prepared in 0.02 M tris-HCl buffer (pH = 7.5), stock solutions of test compounds prepared in DMSO (50 mM), and the experiment conducted in triplicate. To a flat-bottom 96-well transparent plate were added 150µL of treatment solutions with inhibitors **4a-d**, **5a-c**, **6a-c** and donepezil at eight different concentrations

serially diluted. Negative (without treatment) and vehicle (DMSO, final concentration 0.2% v/v for AChE and 0.8% v/v for BuChE) controls were kept for reference. Following, there were added 60 μL of 5,5′-Dithiobis(2-nitrobenzoic acid) (DTNB, Ellman's reagent) at 1.1 mM and 30 μL of electric eel acetylcholinesterase (EeAChE) or equine serum butyrylcholinesterase (EqBuChE) at 0.20 U/mL in the presence of 1 mg/mL bovine serum albumin (BSA). Absorbance was then recorded using an iMark plate reader (Bio-Rad) equipped with a λ = 415 nm light filter and this measure used as a blank reference. After a 10-minute incubation at 30°C, 24 μL of 2.75 mM acetylthiocholine iodide (ACTI) or S-butyrylthiocholine iodide (BCTI) were added and the absorbance recorded after a 10-minute incubation at 30°C at λ = 415 nm for 3 times within 30 seconds. Enzyme activity was calculated as percentage of untreated control discounting the blank reference. The final inhibitor concentration ranged from 100 – 0.00001 μM for AChE (dillution factor = 10) and from 400 – 0.1 μM for BuChE (dillution factor = 4). IC_{50} values were calculated on Graphpad Prism 7.0 using the non-linear regression model for dose-response inhibition.

Enzymatic kinetic study

The enzymatic kinetic of cholinesterase inhibition was determined according to adapted Ellman's method[1]. All of the solution were prepared in 0.02 M tris-HCl buffer (pH = 7.5), stock solutions of test compounds prepared in DMSO (50 mM), and the experiment conducted in triplicate. To a flat-bottom 96-well transparent plate were added 150μL of treatment solutions with inhibitors **4d** and **6a** at two different concentrations **(Table 2)** distributed in eight sets of triplicates each. Eight sets of untreated triplicates were used as negative control. Following, there were added 60 μL of 5,5′-Dithiobis(2-nitrobenzoic acid) (DTNB, Ellman's reagent) at 1.1 mM and 30 μL of electric eel acetylcholinesterase (EeAChE) or equine serum butyrylcholinesterase (EqBuChE) at 0.20 U/mL in presence of 1 mg/mL bovine serum albumin (BSA). Absorbance was then recorded using an iMark plate reader (Bio-Rad) equipped with a λ = 415 nm light filter and this measure used as a blank reference. After 10-minute incubation at room temperature, 24 μL of acetylthiocholine iodide (ACTI) or S-butyrylthiocholine iodide (BCTI) at eight concentrations serially dilluted (factor = 1.3) from 2.75 – 0.44 mM (final concentration: 0.25 – 0.04 mM) were added to all wells and the absorbance recorded after incubation for 0, 5, 10, 15 and 20 minutes at room temperature at λ = 415 nm. The Lineweaver–Burk reciprocal plots were obtained by

plotting a 1/velocity versus 1/[substrate] graph for two different inhibitor concentrations and untreated control. The linear regression of each data-set shows a convergent behavior, in ways the region to where the curves converge determine the type of inhibition observed. Ki, Ki' (competitive and noncompetitive inhibition constants respectively), Km (Michaelis-Menten constant) and Vmax values were calculated with Graphpad Prism 7.0 using the non-linear regression models for enzyme kinetics - inhibiton and enzyme kinects - substrate vs. velocity.

Table 2. Inhibitor concentrations for the kinetic study on AChE and BuChE

**Compound**	**Inhibitor concentrations (µM)**	
	**4d**	**6a**
**AChE**	0.1 and 0.2	0.01 and 0.03
**BuChE**	2 and 5	3 and 6

Molecular modeling

In order to get insight at the molecular level of possible reasons for the observed enzyme inhibition data, we implemented a molecular docking study with an electric eel (*Electrophorus electricus*) acetylcholinesterase (EeAChE) and a horse (*Equus caballus*) butyrylcholinesterase (EqBuChE). Cholinesterases have two important binding sites: the *active site*, where the enzymes hydrolyze their substrates; and the *peripheral site*, both located at the extremities of a gorge in the enzyme structure[2]. Both sites were explored in the docking study, to determine which one is the most suitable for the connection of these compounds.

There are three EeAChE structures available in the Protein Data Bank (PDB): 1EEA[3], 1C2B[4] and 1C2O[4], all of them without any co-crystallized ligand, and we chose 1C2O for the docking studies because it has the best crystallographic resolution (4.2 Å). For EqBuChE, there is no available crystallographic structure, so it was necessary the construction of a 3D model from a sequence available in the UniProtKB/Swiss-Prot protein sequence database (entry Q9N1N9). It was used for construction of a homology model with the automated mode of the protein structure homology-modeling server, Swiss-Model[5], using as template the human BuChE in complex with *N*-((1-(2,3-dihydro-1H-inden-2-yl)piperidin-3-yl)methyl)-*N*-(2-methoxyethyl)-2-naphth-amide (PDB code 4TPK)[6]. The resolution of the template was 2.52 Å, the identity between

sequences was 90.40%, with a coverage 0.95. The global and per-residue model quality has been assessed using the QMEAN scoring function[7].

As a previous test for the ability of the docking program GOLD 5.6 (CCDC Software Ltd., Cambridge, UK) to find reliable solutions for the docking to cholinesterases, we implemented redocking experiments. Since all the three acetylcholinesterases to be tested do not have ligands in their crystallographic structures, all of the scoring functions available in the program were tested through redocking procedures with the PDB structure 2CMF (*Torpedo californica* AChE co-crystallized with alkylene-linked bis-tacrine dimer)[8]. Hydrogen atoms were added to proteins structures based on ionization and tautomeric states defined by GOLD. To allow the best orientation of hydrogen bonds involving the serine, threonine, tyrosine and lysine side chains, they were set free to rotate during the docking procedure. In the course of the searching procedure, 100,000 genetic operations (crossover, migration, mutation) were used for each docking run. Radius of binding sites for the enzymes was tested at 15 Å and 20 Å around atoms from adequate amino acids selected based on literature information for each binding site. The same score function, corresponding amino acids and radius were adopted for the docking studies with the EqBuChE model.

Spartan'14 program [Wavefunction, Inc.] was utilized to construct and optimize the molecules in study, with the PM6 method[9]. Several poses were obtained for each compound in all proteins, and the best-ranked pose for each one was chosen for analysis of the interactions with the amino acid residues. In the GOLD docking program, the docking functions yield the "fitness scores", which are dimensionless values. In each case, the score values are a guide of how good the docking pose is, with a higher score indicating a better docking result.

Ferric reducing ability of plasma (FRAP) antioxidant assay

FRAP solution was freshly prepared by mixing 25 mL acetate buffer (0.30 M) at pH 3.6, 2.5 mL ferric chloride (20 mM) and 2.5 mL 2,4,6-tris(2-pyridyl)-s-triazine (10 mM). In a test tube containing 4.5 mL of FRAP reagent were added 0.5 mL of 500 µM solutions of **4a-d**, **5a-c** and **6a-c** (in methanol). After a 10-minute incubation at 37°C the absorbance was recorded in a spectrophotometer at $\lambda = 593$ nm using methanol as blank reference[11]. A standard curve was obtained by plotting the absorbance read after treatment with 10, 25, 30, 50, 75 and 100 µM quercetin and results, expressed as mmol of quercetin (Q) equivalent to mol of inhibitor (mmol Q/mol), obtained in triplicates.

Murine neuroblastoma cell (N2a) culture and cell viability assay.

N2a cells were cultured in Dulbecco's modified Eagle's medium supplemented with 10% fetal bovine serum and 0.1% gentamicin in a 5% CO_2 atmosphere. N2a cells were transferred to a 96-well plate (~10,000 cells/cm^2) and incubated for 24 h, before treatment with the compounds at 10 or 50 µM. After being treated, cells were grown for 48 hours. Cell viability was evaluated by MTT (3-[4,5-dimethylthiazol-2-yl]-2,5-diphenyl tetrazolium bromide) assay. MTT was added to a final concentration of 0.5 mg/mL into the wells (samples and controls), and the plates were incubated for 4 h at 37 °C. After, all the media was removed and produced formazan crystals were solubilized with DMSO and quantified by reading the OD at 570 nm in a 96-well plate reader (SpectraMax® Paradigm®, Molecular Devices). Means and standard deviations were calculated from triplicates.

Biological assessment results

Enzymatic kinetic study

Table 3. Kinetics parameters of **4d** and **6a** on AChE and BuChE

**Concentration (µM)**	**V_{max} ± SD[a] (µM/min)**	**K_m (µM) ± SD[a]**	**Ki (nM)[b] ± SD[a]**	**Ki' (nM)[c] ± SD[a]**
		**4d on AChE**		
0	3.064 ± 0.063	23.06 ± 2.744	21.273 ± 0.816	157.350 ± 4.674
0.1	2.209 ± 0.060	48.083 ± 2.189		
0.2	1.752 ± 0.198	81.645 ± 7.743		
		**4d on BuChE**		
0	6.306 ± 0.138	78.160 ± 0.141	2129 ± 148	6621 ± 322
0.1	5.791 ± 0.185	97.903 ± 0.309		
0.2	4.669 ± 0.229	114.500 ± 1.061		
		**6a on AChE**		
0	3.168 ± 0.188	25.290 ± 0.651	1.610 ± 0.066	10.499 ± 0.438
0.01	1.762 ± 0.128	56.185 ± 3.910		
0.03	1.254 ± 0.043	76.095 ± 1.025		
		**6a on BuChE**		
0	5.811 ± 0.201	80.190 ± 0.625	2585 ± 236	7376 ± 558
0.01	4.837 ± 0.399	109.483 ± 5.504		
0.03	3.224 ± 0.284	112.827 ± 1.961		

[a] Standard deviation (SD) of triplicate independent experiments

[b] Ki is the competitive inhibition constant

[c] Ki' is the non-competitive inhibition constant

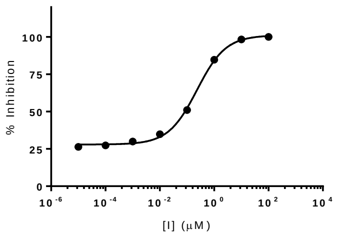

4a

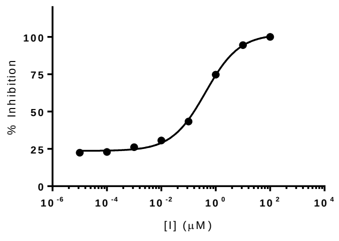

4b

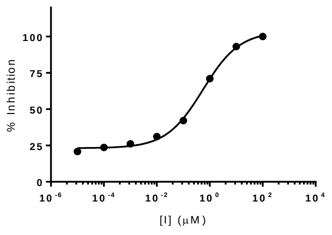

4c

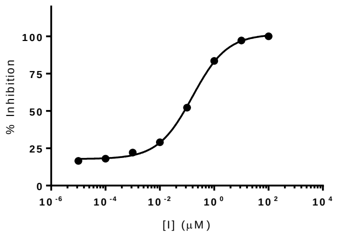

4d

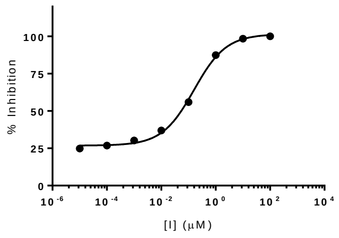

5a

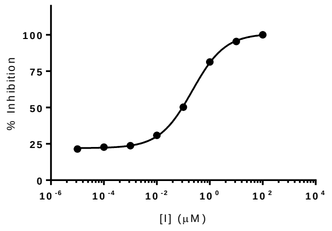

5b

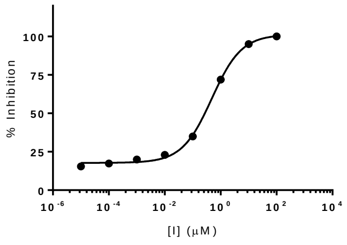

5c

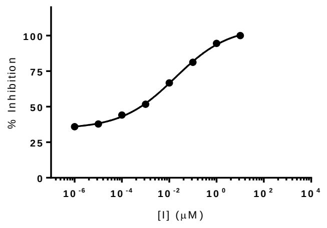

6a

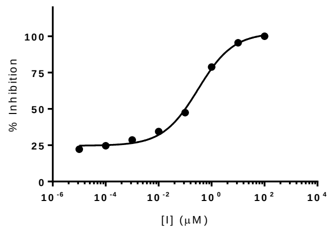

6b

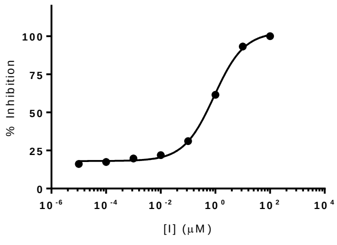

6c

Figure 1. Graphs of AChE inhibition percentage vs. inhibitor concentration

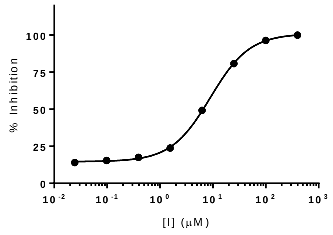

4a

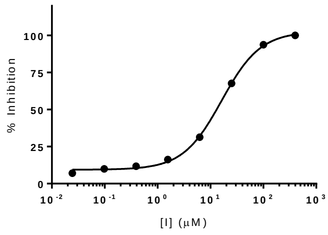

4b

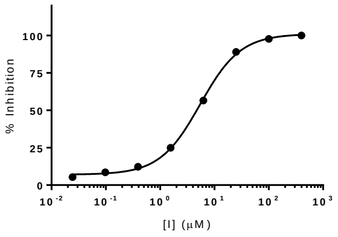

4c

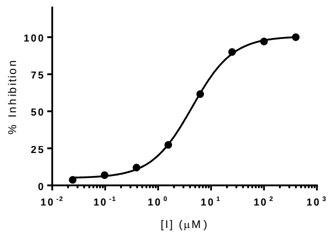

4d

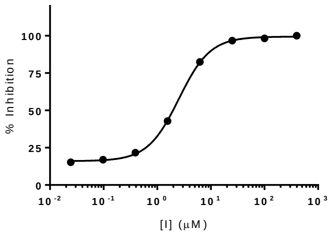

5a

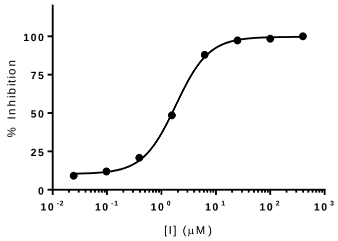

5b

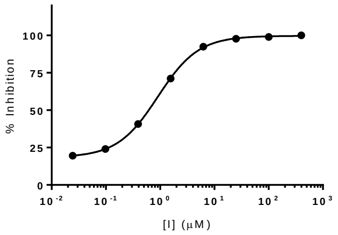

5c

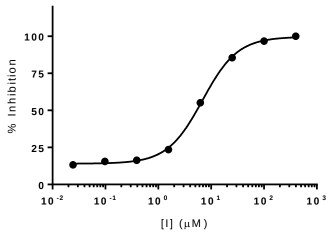

6a

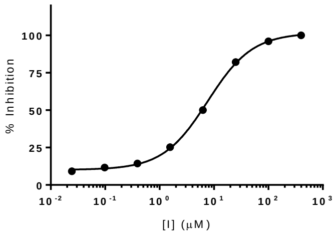

6b

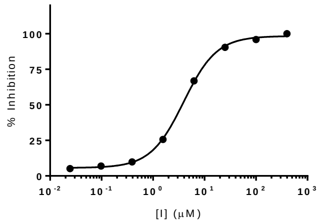

6c

Figure 2. Graphs of BuChE inhibition percentage vs. inhibitor concentration

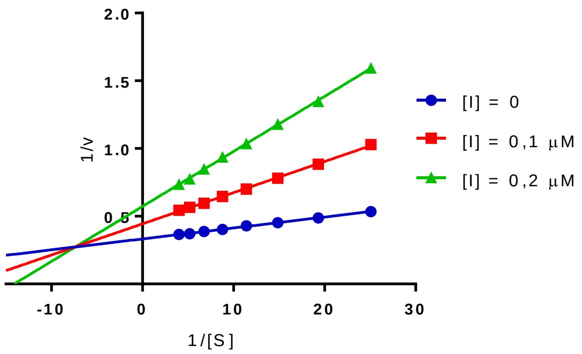

4d

Figure 3. Lineweaver-Burk plots of EeAChE inhibition kinetics of compound **4d**. Inset: concentrations used for **4d** are depicted with [I] graphic symbol.

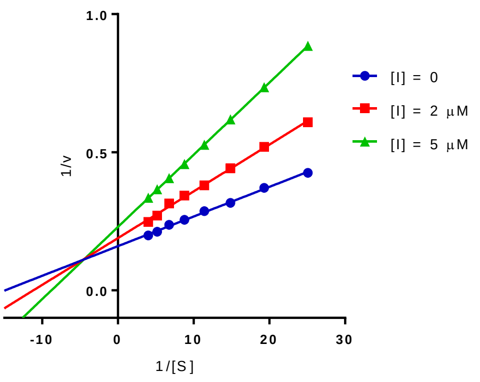

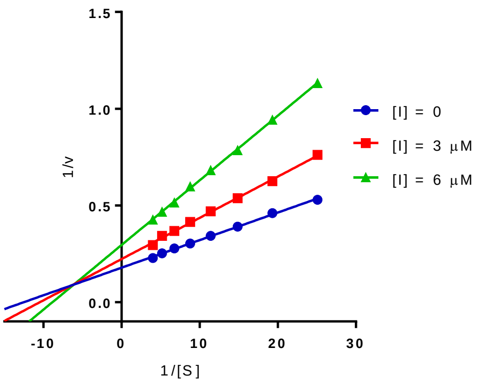

4d **6a**

Figure 4. Lineweaver-Burk plots of EqBuChE inhibition kinetics of compounds **4d** and **6a**. Inset: concentrations used for **4d** and **6a** are depicted with [I] graphic symbol.

Molecular modeling

Table 4. Fitness scores (Goldscore)* obtained by molecular docking in cholinesterases

Ligand	EeAChE	EqBuChE
**4a**	64.9	57.6
**6a**	78.1	63.0
**6c**	71.4	67.9

*GOLD 5.6 program

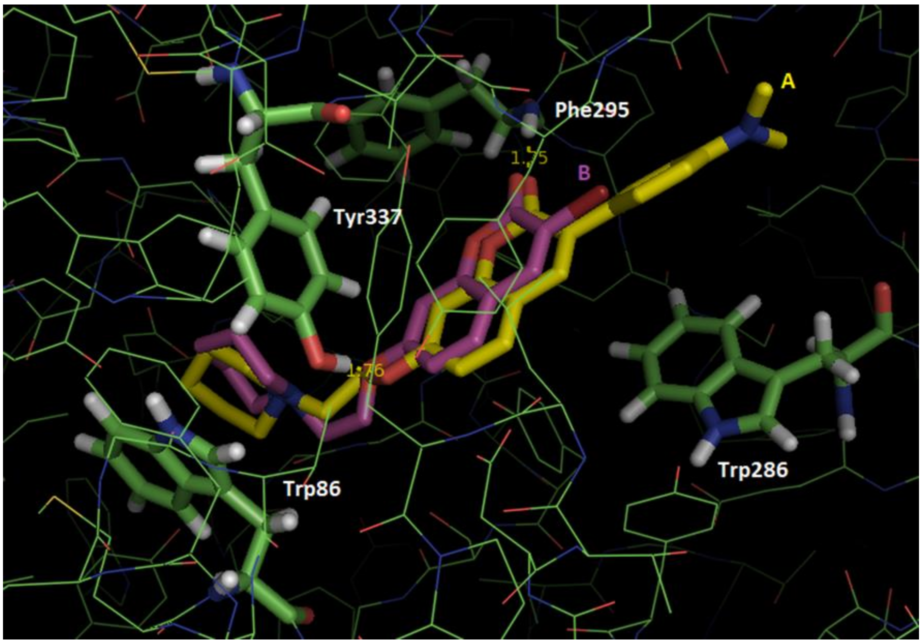

Figure 5. Superposition of the interaction poses of compounds **6a** (A, carbon atoms in yellow) and **4a** (B, carbon atoms in purple) with EeAChE obtained by molecular docking (Goldscore function). Color code: oxygen atoms, red; nitrogen atoms, blue;

hydrogen atoms, white; carbon atoms, green. Figure generated with PyMol 0.99 (DeLano Scientific LLC).

Cytotoxicity Evaluation

Table 5: Neuroblastoma cell viability after compound treatment at 10 and 50 µM.

**Compound**	**Concentration (µM)**	**Mean ± SD**
**4a**	10	89.16 ± 11.28
	50	103.2 ± 13.68
**4b**	10	93.34 ± 8.19
	50	87.60 ± 4.39
**4c**	10	94.43 ± 4.28
	50	88.88 ± 19.01
**4d**	10	80.10 ± 7.27
	50	62.11 ± 10.09
**5a**	10	98.37 ± 8.92
	50	105.5 ± 14.93
**5b**	10	83.55 ± 6.58
	50	57.06 ± 8.72**
**5c**	10	117.4 ± 4.86
	50	13.47 ± 1.19***
**6a**	10	92.15 ± 43.03
	50	115.9 ± 4.28
**6b**	10	84.11 ± 11.1
	50	16.79 ± 8.41***
**6c**	10	59.04 ± 8.21**
	50	10.09 ± 3.09***

MTT reduction was evaluated as described in the Experimental Procedures. Data are expressed as the mean ± standard deviation of the percentage of MTT reduction relative to the value for control cells (cells without treatment, 100% viability). **P < 0.01; ****P < 0.0001.

ADMET Evaluation

Compound 4a

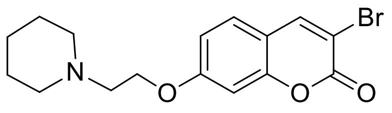

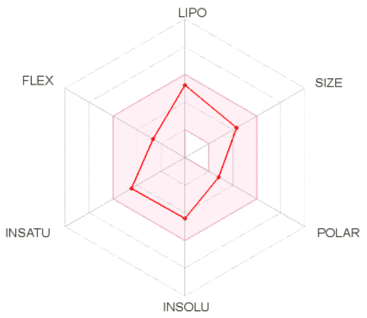

Physicochemical Properties	
Formula	C16H18BrNO3
Molecular weight	352.22 g/mol
Fraction Csp3	0.44
Num. rotatable bonds	4
Num. H-bond acceptors	4
Num. H-bond donors	0
Molar Refractivity	90.21
TPSA	42.68 Å²
**Lipophilicity**	
Log $P_{o/w}$ (iLOGP)	3.40
Log $P_{o/w}$ (XLOGP3)	3.66
Log $P_{o/w}$ (WLOGP)	3.04
Log $P_{o/w}$ (MLOGP)	2.73
Log $P_{o/w}$ (SILICOS-IT)	3.91
Consensus Log $P_{o/w}$	3.35
**Water Solubility**	
Log S (ESOL)	-4.42
Solubility	1.35e-02 mg/ml ; 3.82e-05 mol/l
Class	Moderately soluble
**Pharmacokinetics**	
GI absorption	High
BBB permeant	Yes
P-gp substrate	No
CYP1A2 inhibitor	Yes
CYP2C19 inhibitor	Yes
CYP2C9 inhibitor	Yes
CYP2D6 inhibitor	Yes
CYP3A4 inhibitor	No
**Druglikeness**	
Lipinski	Yes; 0 violation
Ghose	Yes
Veber	Yes
Egan	Yes
Muegge	Yes
Bioavailability Score	0.55

Compound 4b

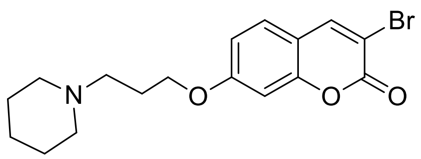

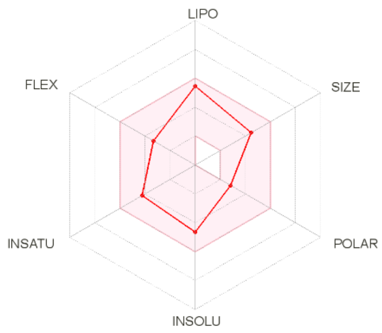

Physicochemical Properties	
Formula	C17H20BrNO3
Molecular weight	366.25 g/mol
Fraction Csp3	0.47
Num. rotatable bonds	5
Num. H-bond acceptors	4
Num. H-bond donors	0
Molar Refractivity	95.02
TPSA	42.68 Å²
**Lipophilicity**	
Log $P_{o/w}$ (iLOGP)	3.62
Log $P_{o/w}$ (XLOGP3)	4.02
Log $P_{o/w}$ (WLOGP)	3.43
Log $P_{o/w}$ (MLOGP)	2.97
Log $P_{o/w}$ (SILICOS-IT)	4.29
Consensus Log $P_{o/w}$	3.67
**Water Solubility**	
Log S (ESOL)	-4.65
Solubility	8.20e-03 mg/ml ; 2.24e-05 mol/l
Class	Moderately soluble
**Pharmacokinetics**	
GI absorption	High
BBB permeant	Yes
P-gp substrate	No
CYP1A2 inhibitor	Yes
CYP2C19 inhibitor	Yes
CYP2C9 inhibitor	Yes
CYP2D6 inhibitor	Yes
CYP3A4 inhibitor	No
**Druglikeness**	
Lipinski	Yes; 0 violation
Ghose	Yes
Veber	Yes
Egan	Yes
Muegge	Yes
Bioavailability Score	0.55

Compound 4c

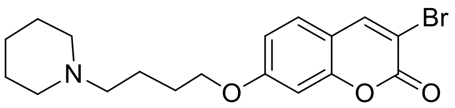

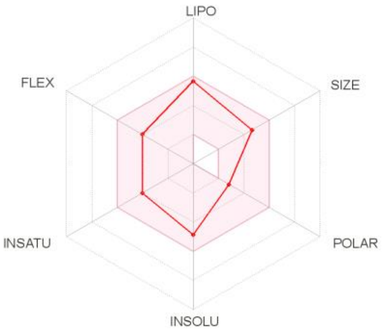

Physicochemical Properties	
Formula	C18H22BrNO3
Molecular weight	380.28 g/mol
Fraction Csp3	0.50
Num. rotatable bonds	6
Num. H-bond acceptors	4
Num. H-bond donors	0
Molar Refractivity	99.83
TPSA	42.68 Å^2
**Lipophilicity**	
Log $P_{o/w}$ (iLOGP)	3.90
Log $P_{o/w}$ (XLOGP3)	4.38
Log $P_{o/w}$ (WLOGP)	3.82
Log $P_{o/w}$ (MLOGP)	3.20
Log $P_{o/w}$ (SILICOS-IT)	4.66
Consensus Log $P_{o/w}$	3.99
**Water Solubility**	
Log S (ESOL)	-4.88
Solubility	4.98e-03 mg/ml ; 1.31e-05 mol/l
Class	Moderately soluble
**Pharmacokinetics**	
GI absorption	High
BBB permeant	Yes
P-gp substrate	No
CYP1A2 inhibitor	Yes
CYP2C19 inhibitor	Yes
CYP2C9 inhibitor	Yes
CYP2D6 inhibitor	Yes
CYP3A4 inhibitor	No
**Druglikeness**	
Lipinski	Yes; 0 violation
Ghose	Yes
Veber	Yes
Egan	Yes
Muegge	Yes
Bioavailability Score	0.55

Compound 4d

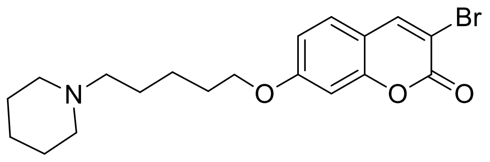

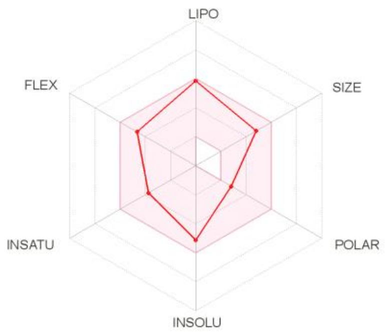

Physicochemical Properties	
Formula	C19H24BrNO3
Molecular weight	394.30 g/mol
Fraction Csp3	0.53
Num. rotatable bonds	7
Num. H-bond acceptors	4
Num. H-bond donors	0
Molar Refractivity	104.64
TPSA	42.68 Å²
**Lipophilicity**	
Log $P_{o/w}$ (iLOGP)	4.15
Log $P_{o/w}$ (XLOGP3)	4.74
Log $P_{o/w}$ (WLOGP)	4.21
Log $P_{o/w}$ (MLOGP)	3.42
Log $P_{o/w}$ (SILICOS-IT)	5.05
Consensus Log $P_{o/w}$	4.31
**Water Solubility**	
Log *S* (ESOL)	-5.12
Solubility	3.01e-03 mg/ml ; 7.63e-06 mol/l
Class	Moderately soluble
**Pharmacokinetics**	
GI absorption	High
BBB permeant	Yes
P-gp substrate	No
CYP1A2 inhibitor	Yes
CYP2C19 inhibitor	Yes
CYP2C9 inhibitor	Yes
CYP2D6 inhibitor	Yes
CYP3A4 inhibitor	Yes
**Druglikeness**	
Lipinski	Yes; 0 violation
Ghose	Yes
Veber	Yes
Egan	Yes
Muegge	Yes
Bioavailability Score	0.55

Compound 5a

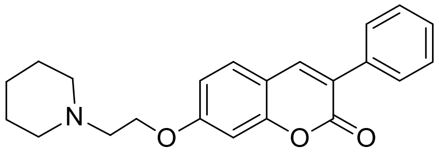

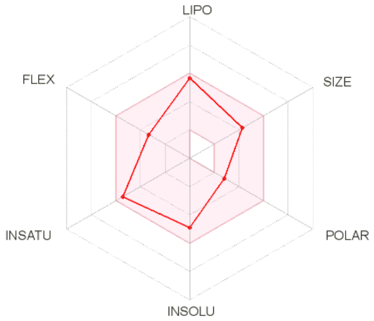

Physicochemical Properties	
Formula	C22H23NO3
Molecular weight	349.42 g/mol
Fraction Csp3	0.32
Num. rotatable bonds	5
Num. H-bond acceptors	4
Num. H-bond donors	0
Molar Refractivity	107.95
TPSA	42.68 Å²
**Lipophilicity**	
Log $P_{o/w}$ (iLOGP)	3.87
Log $P_{o/w}$ (XLOGP3)	4.40
Log $P_{o/w}$ (WLOGP)	3.94
Log $P_{o/w}$ (MLOGP)	3.26
Log $P_{o/w}$ (SILICOS-IT)	4.81
Consensus Log $P_{o/w}$	4.06
**Water Solubility**	
Log *S* (ESOL)	-4.90
Solubility	4.36e-03 mg/ml ; 1.25e-05 mol/l
Class	Moderately soluble
**Pharmacokinetics**	
GI absorption	High
BBB permeant	Yes
P-gp substrate	No
CYP1A2 inhibitor	Yes
CYP2C19 inhibitor	Yes
CYP2C9 inhibitor	Yes
CYP2D6 inhibitor	Yes
CYP3A4 inhibitor	Yes
**Druglikeness**	
Lipinski	Yes; 0 violation
Ghose	Yes
Veber	Yes
Egan	Yes
Muegge	Yes
Bioavailability Score	0.55

Compound 5b

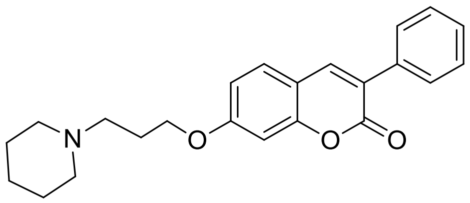

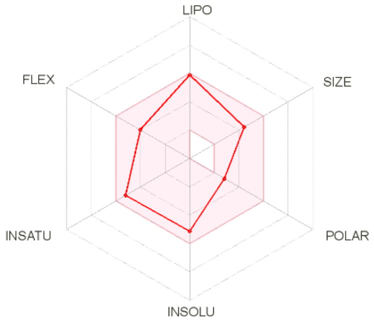

Physicochemical Properties	
Formula	C23H25NO3
Molecular weight	363.45 g/mol
Fraction Csp3	0.35
Num. rotatable bonds	6
Num. H-bond acceptors	4
Num. H-bond donors	0
Molar Refractivity	112.76
TPSA	42.68 Å²
**Lipophilicity**	
Log $P_{o/w}$ (iLOGP)	4.13
Log $P_{o/w}$ (XLOGP3)	4.76
Log $P_{o/w}$ (WLOGP)	4.33
Log $P_{o/w}$ (MLOGP)	3.47
Log $P_{o/w}$ (SILICOS-IT)	5.20
Consensus Log $P_{o/w}$	4.38
**Water Solubility**	
Log *S* (ESOL)	-5.13
Solubility	2.67e-03 mg/ml ; 7.33e-06 mol/l
Class	Moderately soluble
**Pharmacokinetics**	
GI absorption	High
BBB permeant	Yes
P-gp substrate	Yes
CYP1A2 inhibitor	Yes
CYP2C19 inhibitor	Yes
CYP2C9 inhibitor	Yes
CYP2D6 inhibitor	Yes
CYP3A4 inhibitor	Yes
**Druglikeness**	
Lipinski	Yes; 0 violation
Ghose	Yes
Veber	Yes
Egan	Yes
Muegge	Yes
Bioavailability Score	0.55

Compound 5c

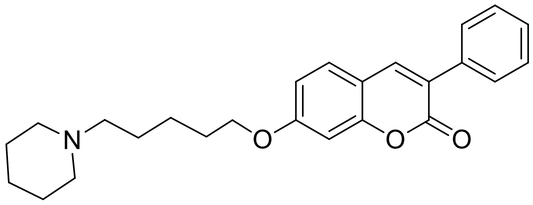

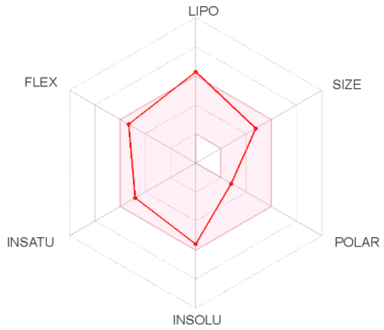

Physicochemical Properties	
Formula	C25H29NO3
Molecular weight	391.50 g/mol
Fraction Csp3	0.40
Num. rotatable bonds	8
Num. H-bond acceptors	4
Num. H-bond donors	0
Molar Refractivity	122.37
TPSA	42.68 Å²
**Lipophilicity**	
Log $P_{o/w}$ (iLOGP)	4.53
Log $P_{o/w}$ (XLOGP3)	5.47
Log $P_{o/w}$ (WLOGP)	5.11
Log $P_{o/w}$ (MLOGP)	3.89
Log $P_{o/w}$ (SILICOS-IT)	5.98
Consensus Log $P_{o/w}$	5.00
**Water Solubility**	
Log *S* (ESOL)	-5.59
Solubility	9.98e-04 mg/ml ; 2.55e-06 mol/l
Class	Moderately soluble
**Pharmacokinetics**	
GI absorption	High
BBB permeant	Yes
P-gp substrate	Yes
CYP1A2 inhibitor	Yes
CYP2C19 inhibitor	Yes
CYP2C9 inhibitor	Yes
CYP2D6 inhibitor	Yes
CYP3A4 inhibitor	Yes
**Druglikeness**	
Lipinski	Yes; 0 violation
Ghose	Yes
Veber	Yes
Egan	Yes
Muegge	No; 1 violation: XLOGP3>5
Bioavailability Score	0.55

Compound 6a

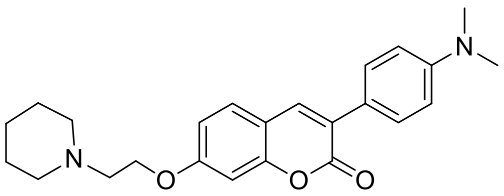

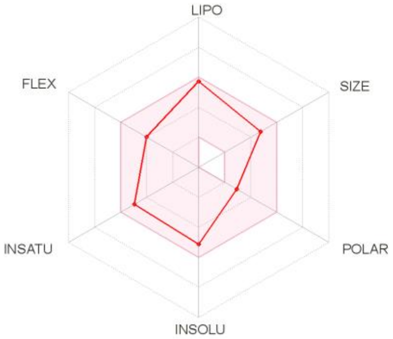

Physicochemical Properties	
Formula	C24H28N2O3
Molecular weight	392.49 g/mol
Fraction Csp3	0.38
Num. rotatable bonds	6
Num. H-bond acceptors	4
Num. H-bond donors	0
Molar Refractivity	122.16
TPSA	45.92 Å²
**Lipophilicity**	
Log $P_{o/w}$ (iLOGP)	4.12
Log $P_{o/w}$ (XLOGP3)	4.52
Log $P_{o/w}$ (WLOGP)	4.01
Log $P_{o/w}$ (MLOGP)	3.12
Log $P_{o/w}$ (SILICOS-IT)	4.49
Consensus Log $P_{o/w}$	4.05
**Water Solubility**	
Log *S* (ESOL)	-5.13
Solubility	2.89e-03 mg/ml ; 7.36e-06 mol/l
Class	Moderately soluble
**Pharmacokinetics**	
GI absorption	High
BBB permeant	Yes
P-gp substrate	No
CYP1A2 inhibitor	Yes
CYP2C19 inhibitor	Yes
CYP2C9 inhibitor	Yes
CYP2D6 inhibitor	Yes
CYP3A4 inhibitor	Yes
**Druglikeness**	
Lipinski	Yes; 0 violation
Ghose	Yes
Veber	Yes
Egan	Yes
Muegge	Yes
Bioavailability Score	0.55

Compound 6b

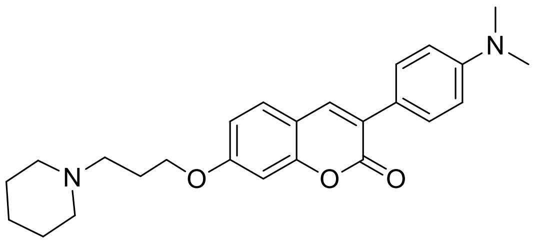

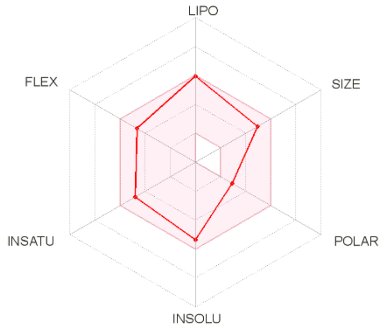

**Physicochemical Properties**	
Formula	C25H30N2O3
Molecular weight	406.52 g/mol
Fraction Csp3	0.40
Num. rotatable bonds	7
Num. H-bond acceptors	4
Num. H-bond donors	0
Molar Refractivity	126.97
TPSA	45.92 Å²
**Lipophilicity**	
Log $P_{o/w}$ (iLOGP)	4.40
Log $P_{o/w}$ (XLOGP3)	4.88
Log $P_{o/w}$ (WLOGP)	4.40
Log $P_{o/w}$ (MLOGP)	3.33
Log $P_{o/w}$ (SILICOS-IT)	4.88
Consensus Log $P_{o/w}$	4.38
**Water Solubility**	
Log *S* (ESOL)	-5.37
Solubility	1.74e-03 mg/ml ; 4.29e-06 mol/l
Class	Moderately soluble
**Pharmacokinetics**	
GI absorption	High
BBB permeant	Yes
P-gp substrate	Yes
CYP1A2 inhibitor	No
CYP2C19 inhibitor	Yes
CYP2C9 inhibitor	Yes
CYP2D6 inhibitor	Yes
CYP3A4 inhibitor	Yes
**Druglikeness**	
Lipinski	Yes; 0 violation
Ghose	Yes
Veber	Yes
Egan	Yes
Muegge	Yes
Bioavailability Score	0.55

Compound 6c

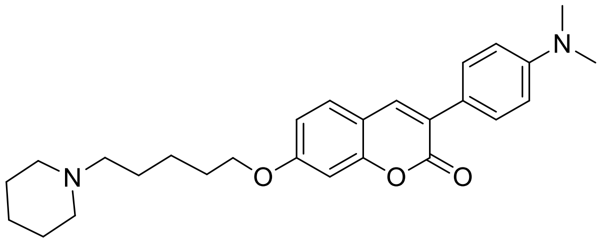

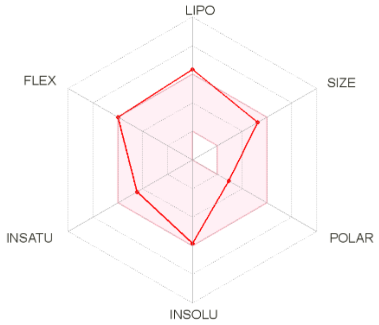

Physicochemical Properties	
Formula	$C_{27}H_{34}N_2O_3$
Molecular weight	434.57 g/mol
Fraction Csp3	0.44
Num. rotatable bonds	9
Num. H-bond acceptors	4
Num. H-bond donors	0
Molar Refractivity	136.58
TPSA	45.92 Å^2
**Lipophilicity**	
Log $P_{o/w}$ (iLOGP)	4.84
Log $P_{o/w}$ (XLOGP3)	5.59
Log $P_{o/w}$ (WLOGP)	5.18
Log $P_{o/w}$ (MLOGP)	3.73
Log $P_{o/w}$ (SILICOS-IT)	5.68
Consensus Log $P_{o/w}$	5.01
**Water Solubility**	
Log S (ESOL)	-5.83
Solubility	6.40e-04 mg/ml ; 1.47e-06 mol/l
Class	Moderately soluble
**Pharmacokinetics**	
GI absorption	High
BBB permeant	Yes
P-gp substrate	Yes
CYP1A2 inhibitor	No
CYP2C19 inhibitor	No
CYP2C9 inhibitor	No
CYP2D6 inhibitor	Yes
CYP3A4 inhibitor	Yes
**Druglikeness**	
Lipinski	Yes; 0 violation
Ghose	No; 1 violation: MR>130
Veber	Yes
Egan	Yes
Muegge	No; 1 violation: XLOGP3>5
Bioavailability Score	0.55

References

1. Ellman GL, Courtney KD, Andres V, et al. A new and rapid colorimetric determination of acetylcholinesterase activity. Biochem Pharmacol 1961;7:88–95.
2. Pezzementi L, Nachon F, Chatonnet A. Evolution of acetylcholinesterase and butyrylcholinesterase in the vertebrates: an atypical butyrylcholinesterase from the medaka *Oryziaslatipes*. Plos One 2011;6:e17396.
3. Raves ML, et al. Quaternary Structure of Tetrameric Acetylcholinesterase. In: Doctor B.P., Taylor P., Quinn D.M., Rotundo R.L., Gentry M.K. (eds) Structure and Function of Cholinesterases and Related Proteins. Springer, Boston, MA [DOI:https://doi.org/10.1007/978-1-4899-1540-5_97]
4. Bourne Y, Grassi J, Bougis PE, et al. Conformational Flexibility of the Acetylcholinesterase Tetramer Suggested by X-ray Crystallography. Journal of Biol Chem 1999;274:30370-30376.
5. Biasini M, Bienert S, Waterhouse A, et al. SWISS-MODEL: modelling protein tertiary and quaternary structure using evolutionary information. Nuc Acids Res 2014;42:W252-W258
6. Brus B, Kosak U, Turk S, et al. Discovery, biological evaluation, and crystal structure of a novel nanomolar selective butyrylcholinesterase inhibitor. J Med Chem 2014;57:8167-79.
7. Benkert P, Biasini M, Schwede T. Toward the estimation of the absolute quality of individual protein structure models. Bioinformatics 2011;27:343-50.
8. Rydberg EH, Brumshtein B, Greenblatt HM, et al. Complexes of Alkylene-Linked Tacrine Dimers with *Torpedo californica* Acetylcholinesterase: Binding of Bis5-Tacrine Produces a Dramatic Rearrangement in the Active-Site Gorge. *J Med Chem* **2006**;49:5491-500.
9. Stewart JJP. Optimization of Parameters for Semiempirical Methods V: Modification of NDDO approximations and application to 70 elements. J Mol Model 2007;13:1173-213.
10. Lesjak M, Beara I, Simin N, Pintać D, et al. Antioxidant and anti-inflammatory activities of quercetin and its derivatives. J Func Foods 2018;40:68-75.
11. Jones G, Willett P, Glen RC, et al. Development and Validation of a Genetic Algorithm for Flexible Docking. J Mol Biol 1997;267:727-48.

^{1}H NMR spectra of **2a**

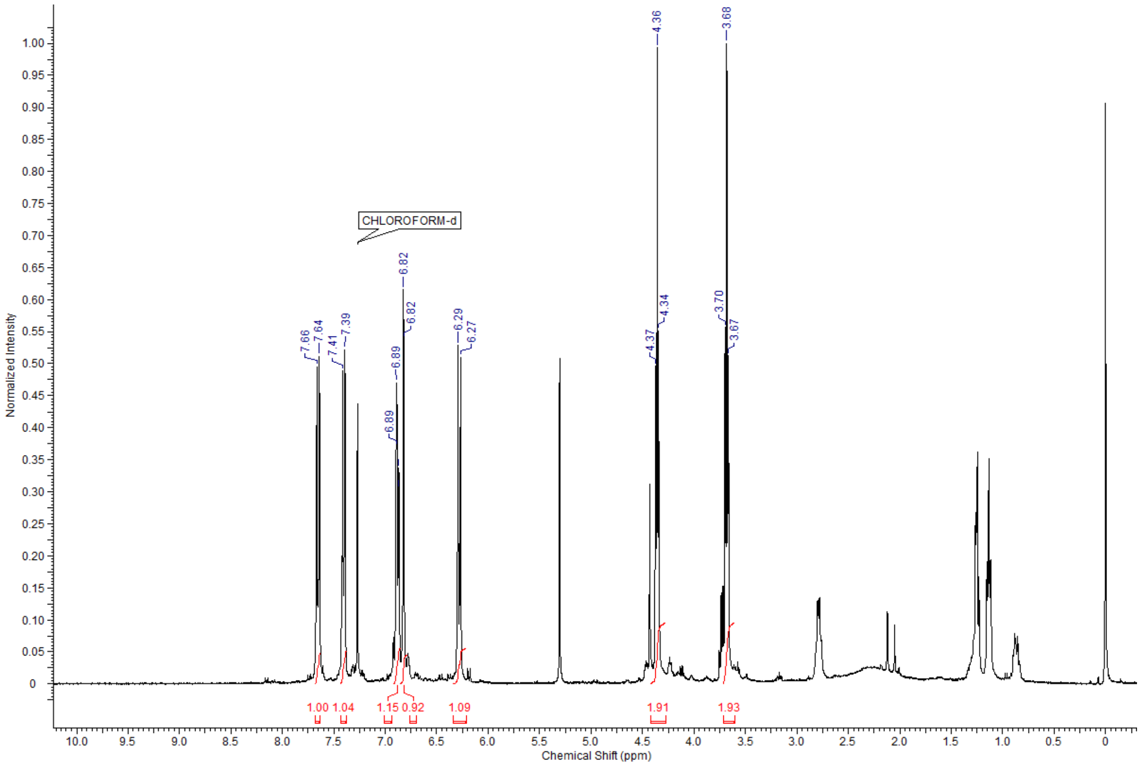

^{13}C NMR espectra of **2a**

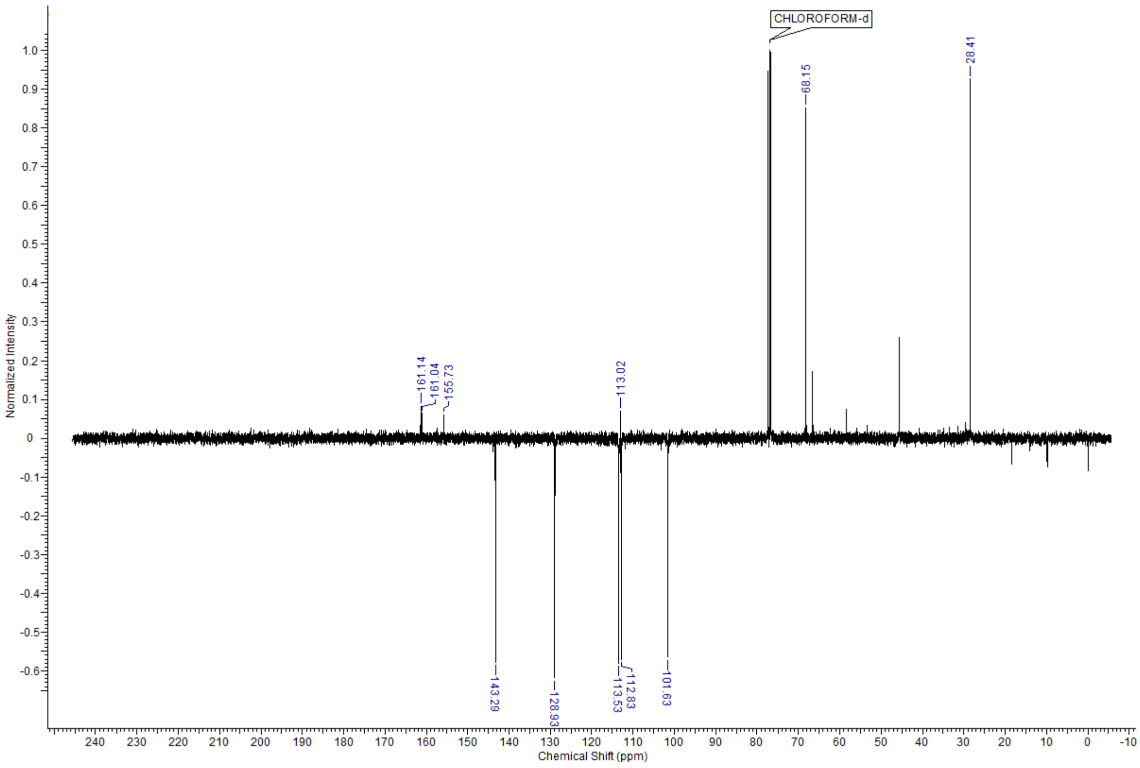

^{1}H NMR spectra of **2b**

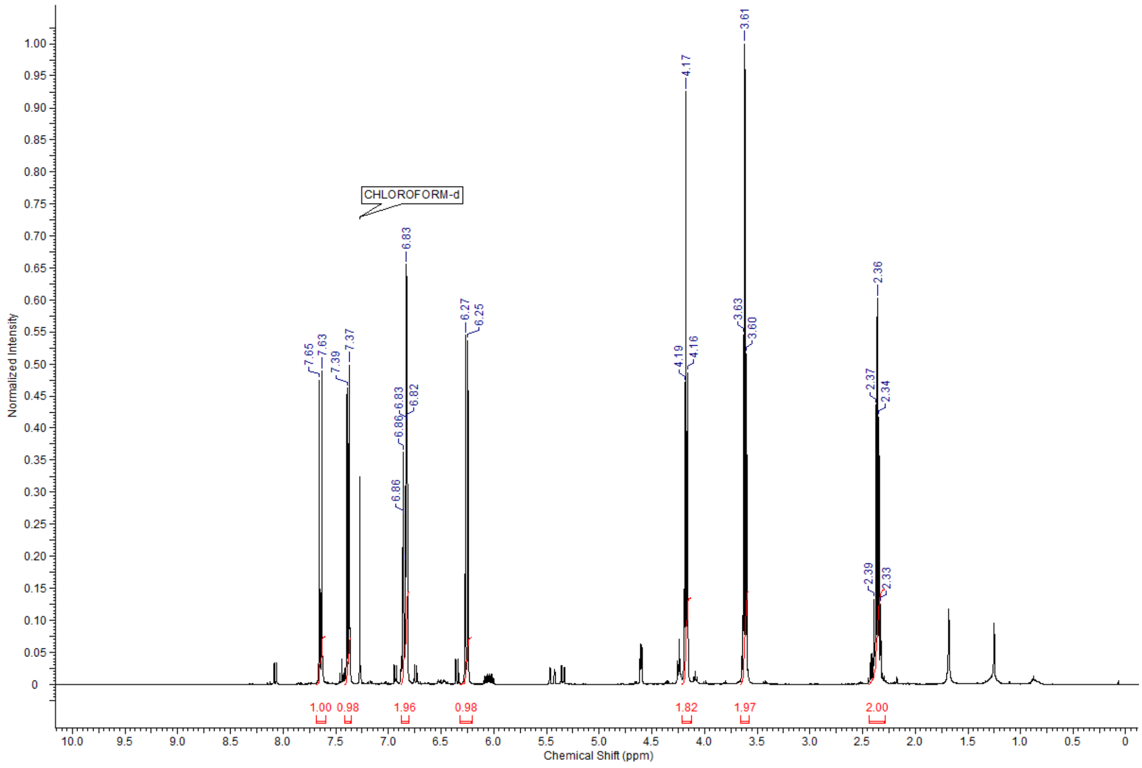

^{13}C NMR espectra of **2b**

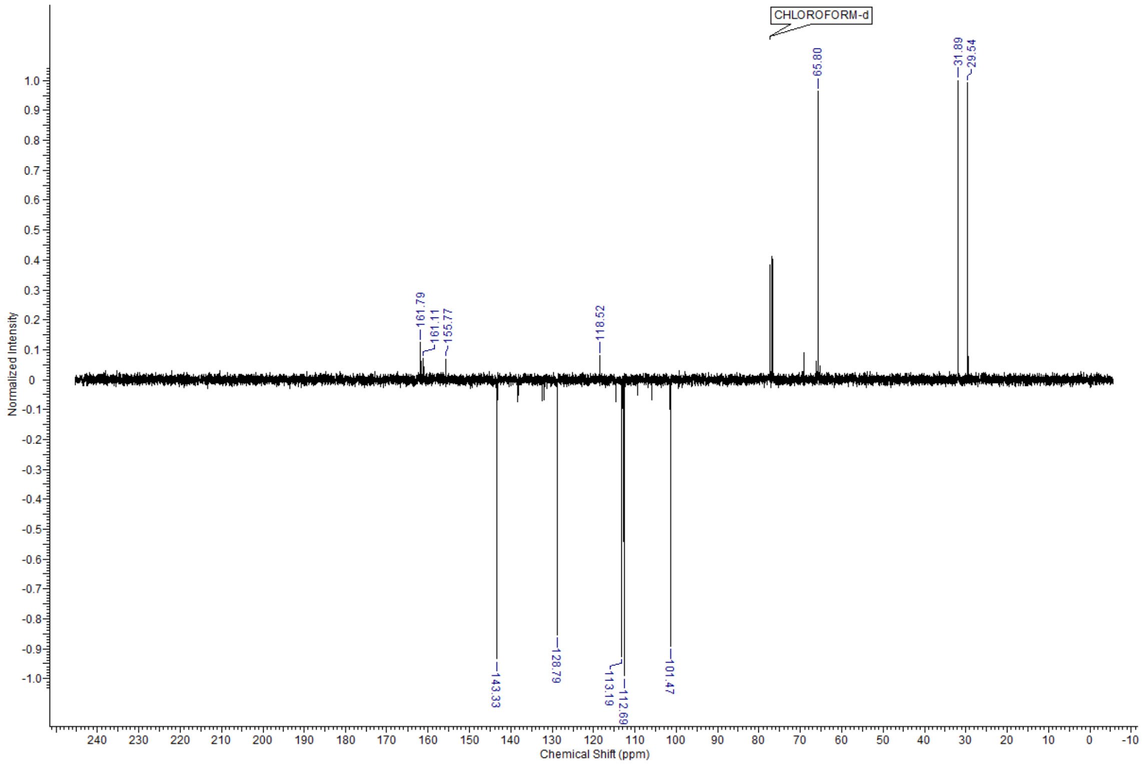

^{1}H NMR spectra of **2c**

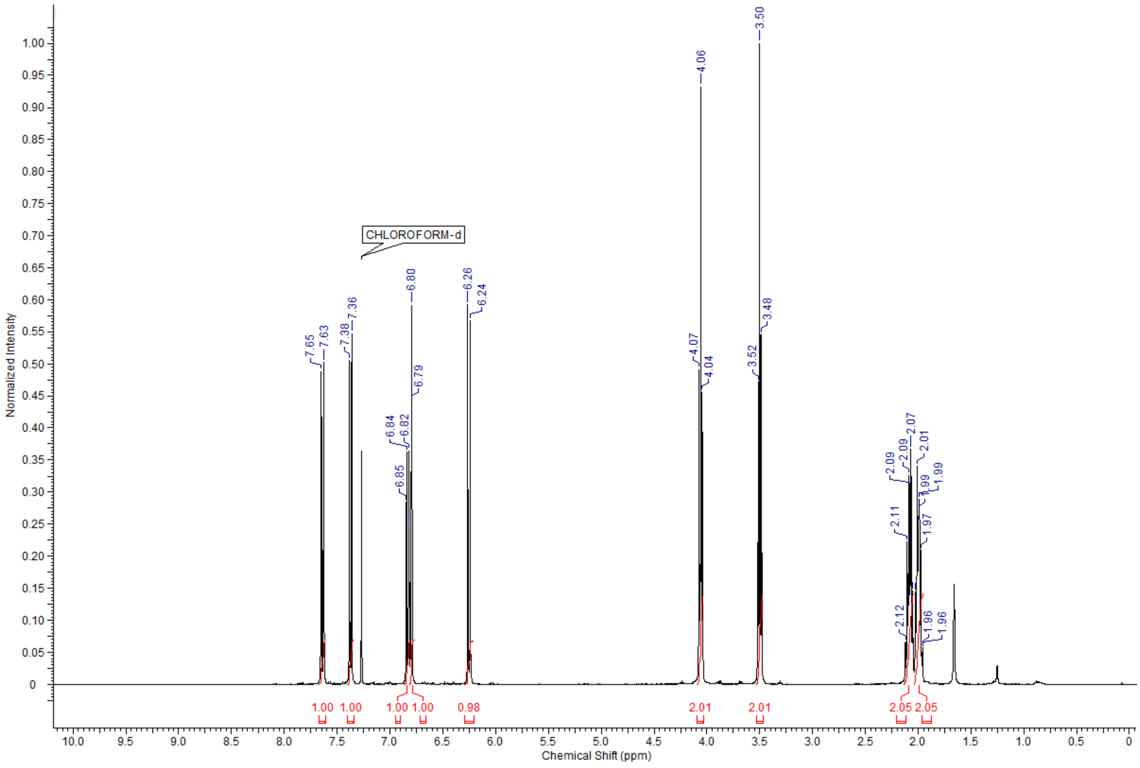

^{13}C NMR espectra of **2c**

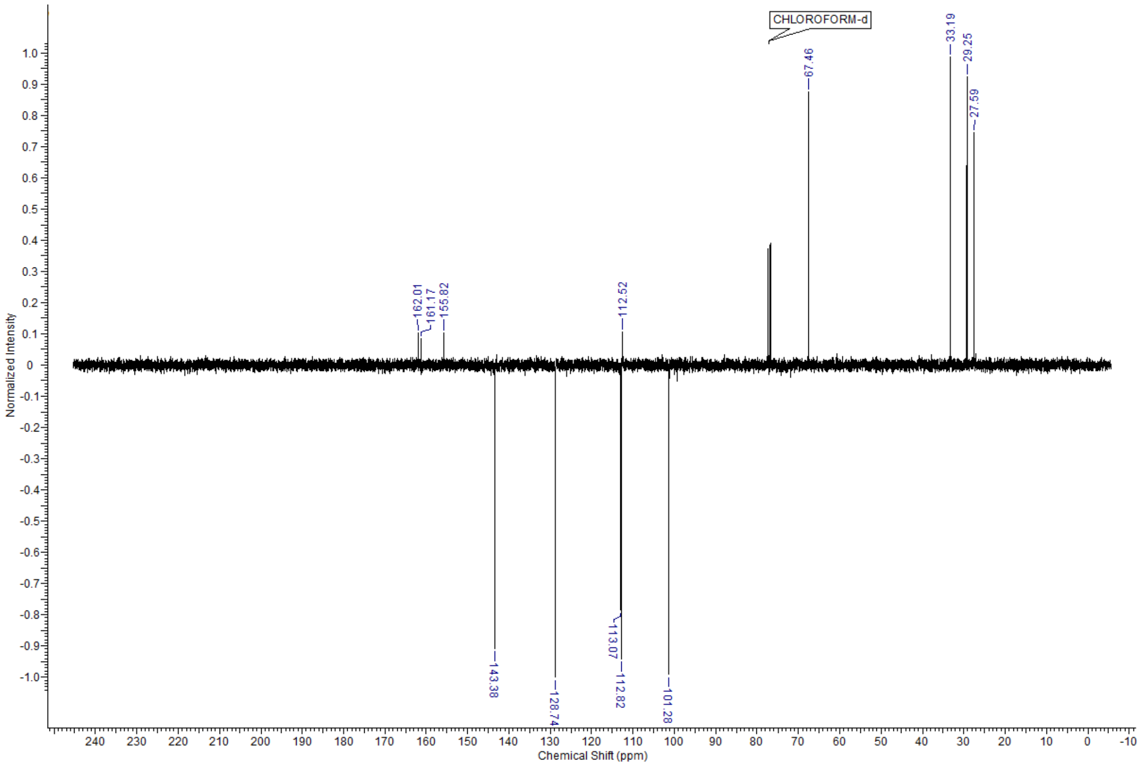

^{1}H NMR spectra of **2d**

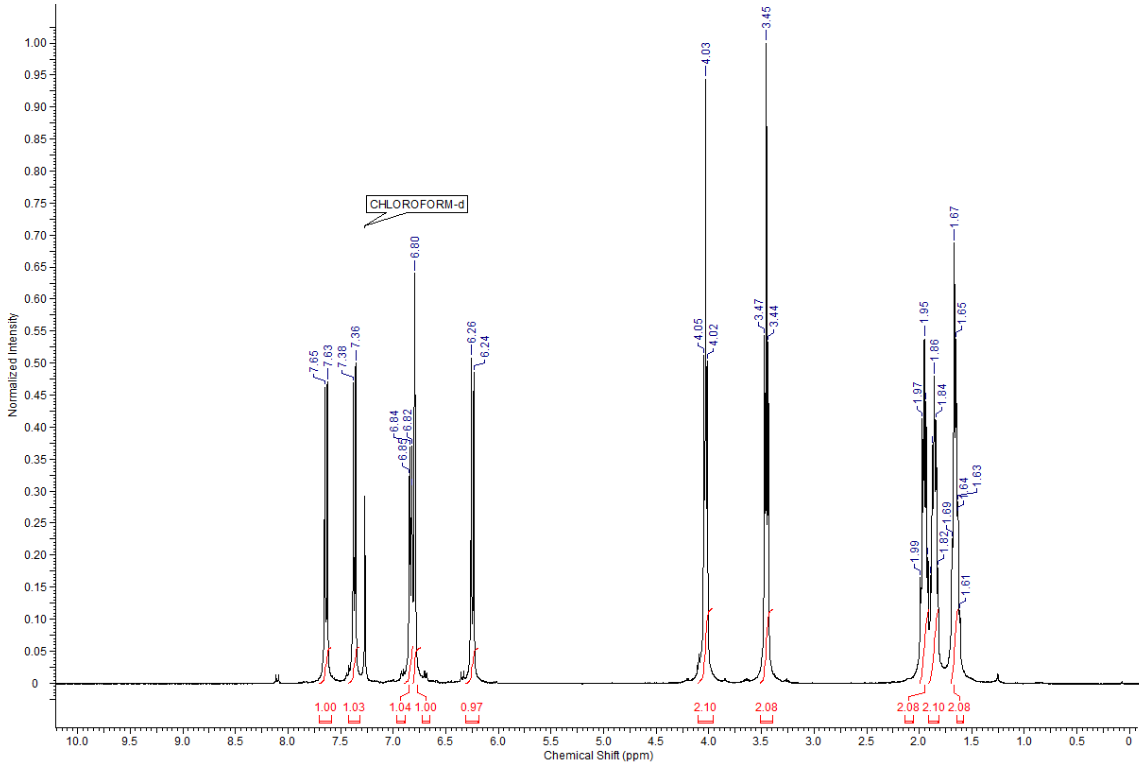

^{13}C NMR espectra of **2d**

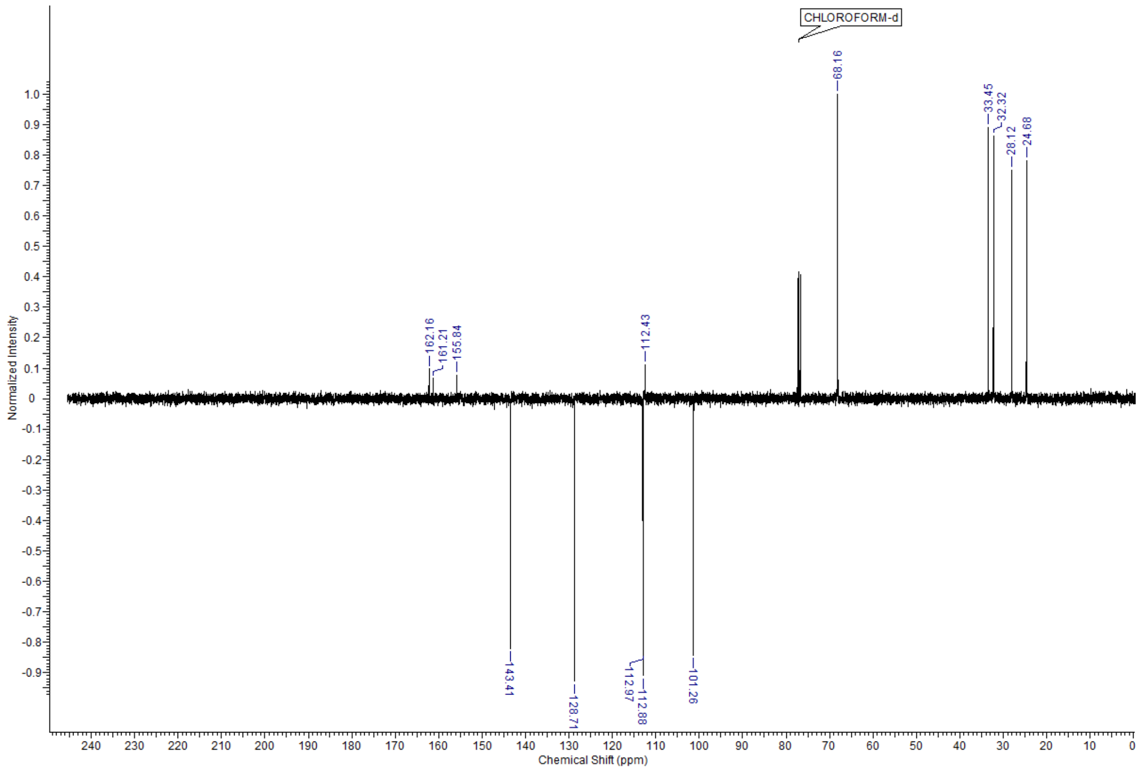

^{1}H NMR spectra of **3a**

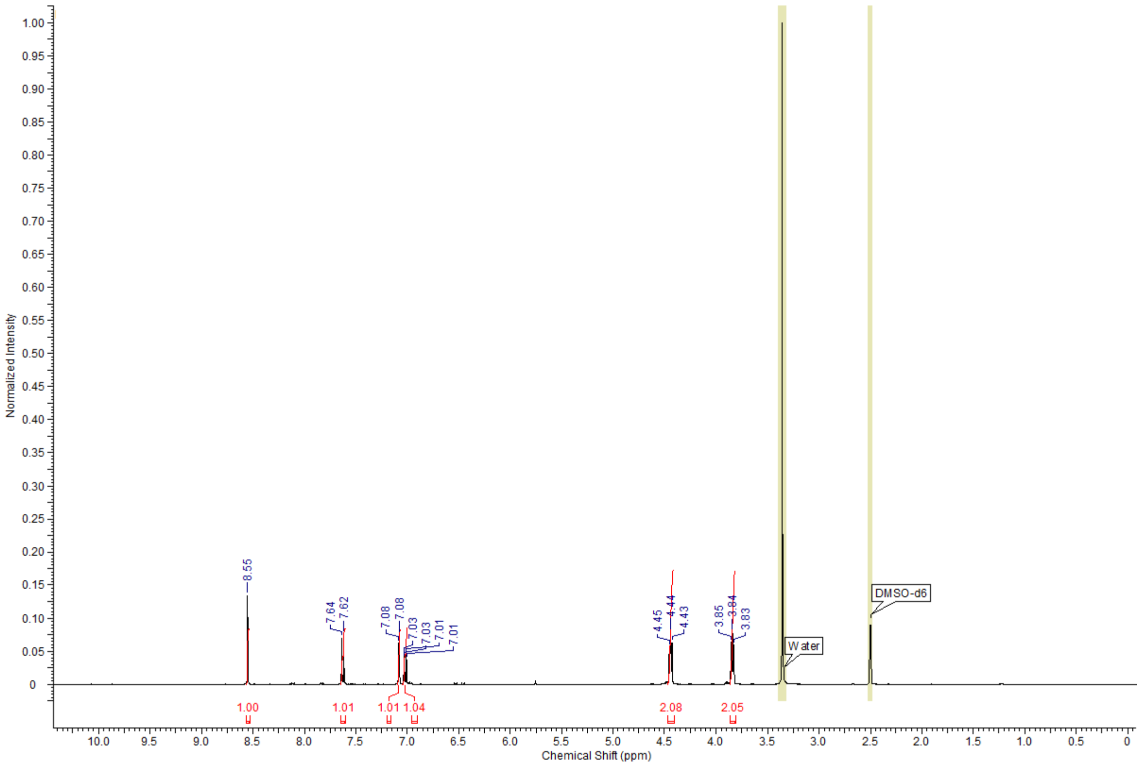

^{13}C NMR espectra of **3a**

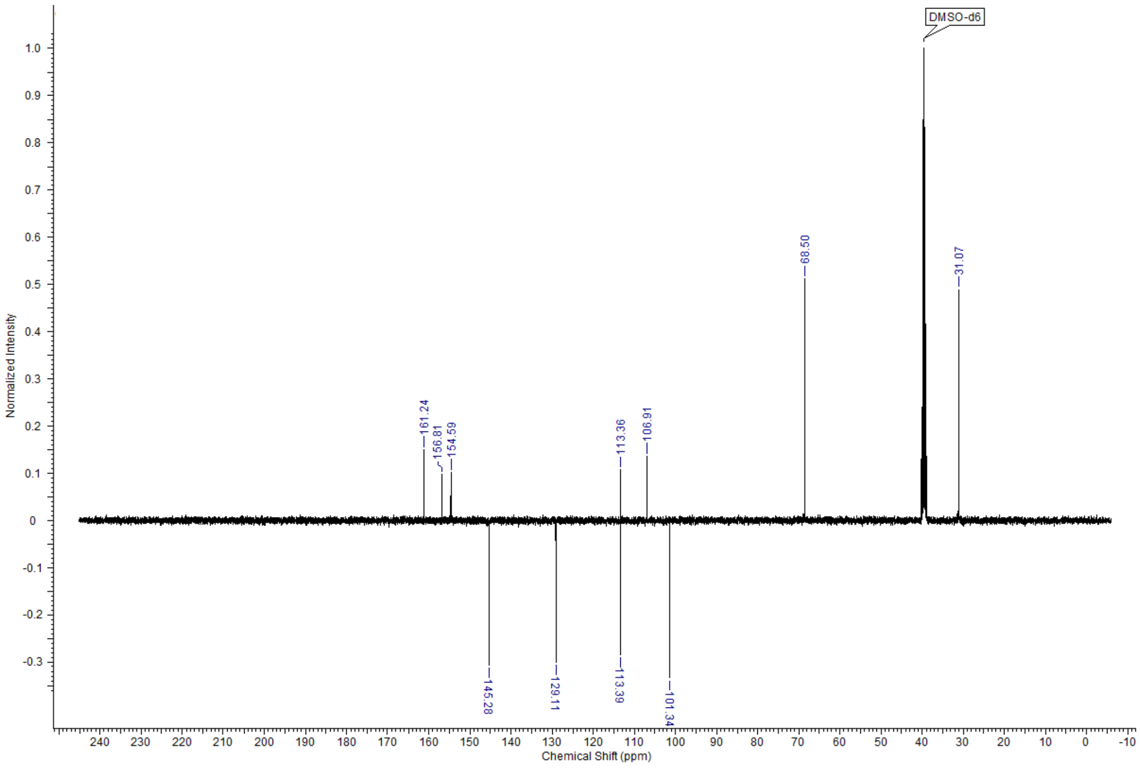

^{1}H NMR spectra of **3b**

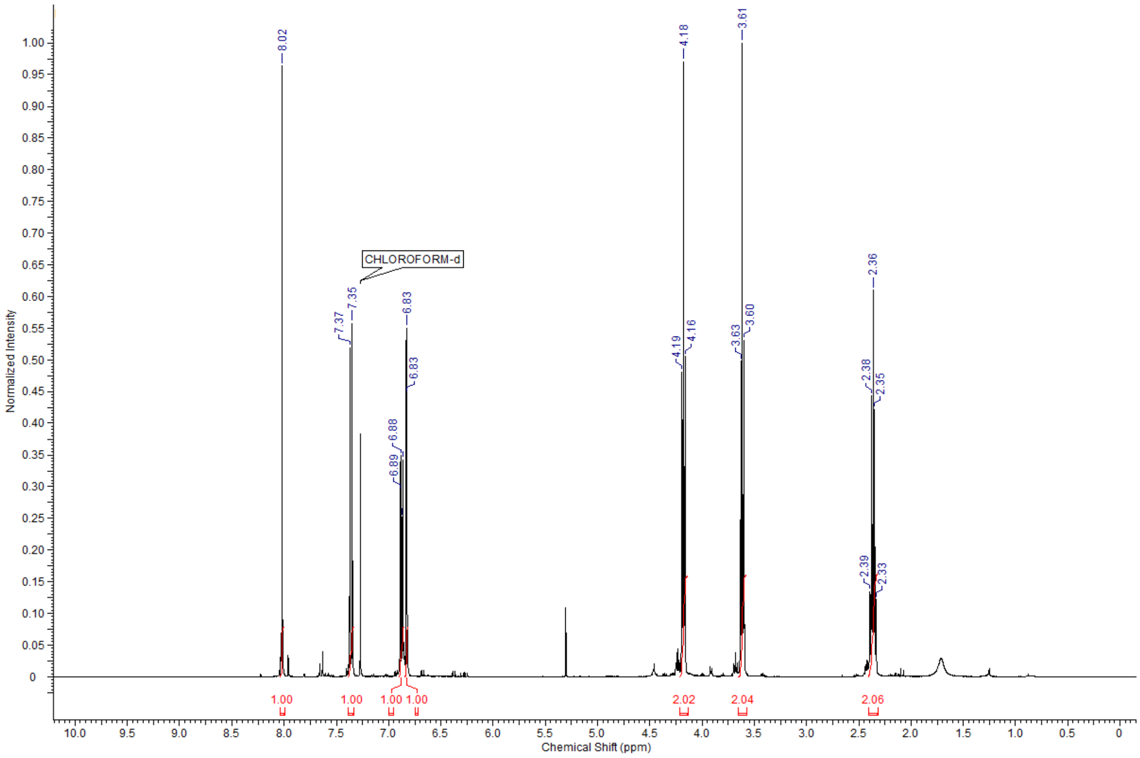

^{13}C NMR espectra of **3b**

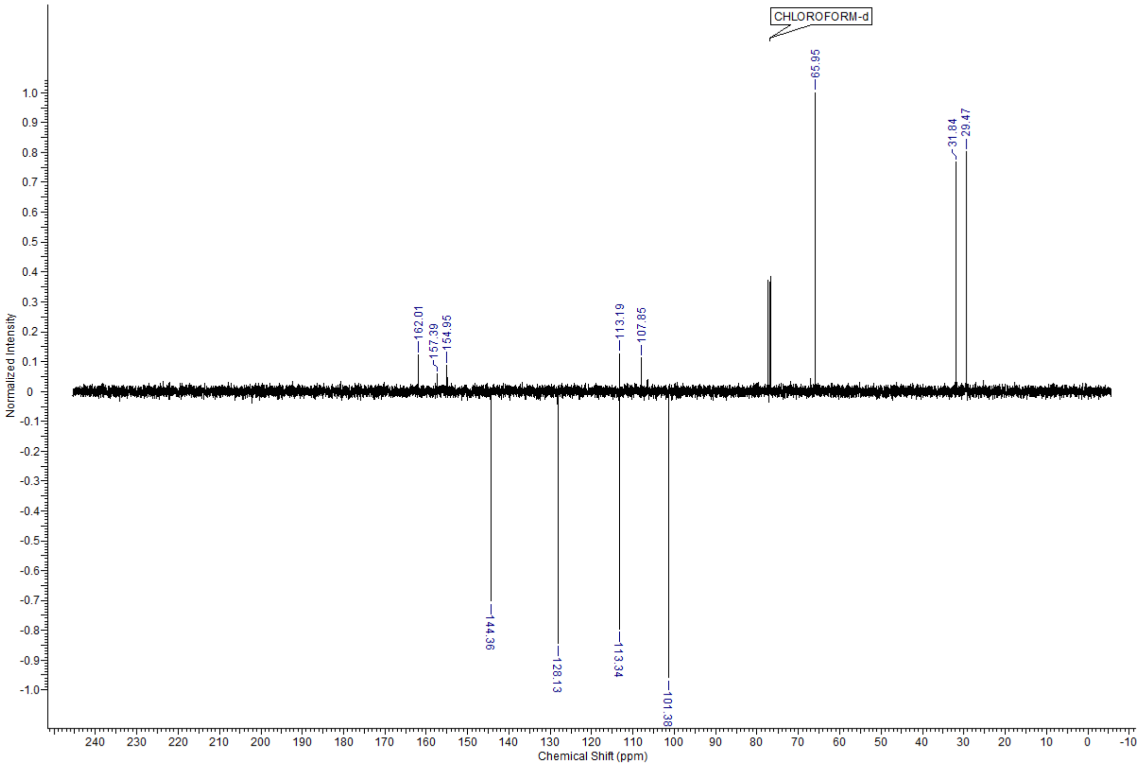

^{1}H NMR spectra of **3c**

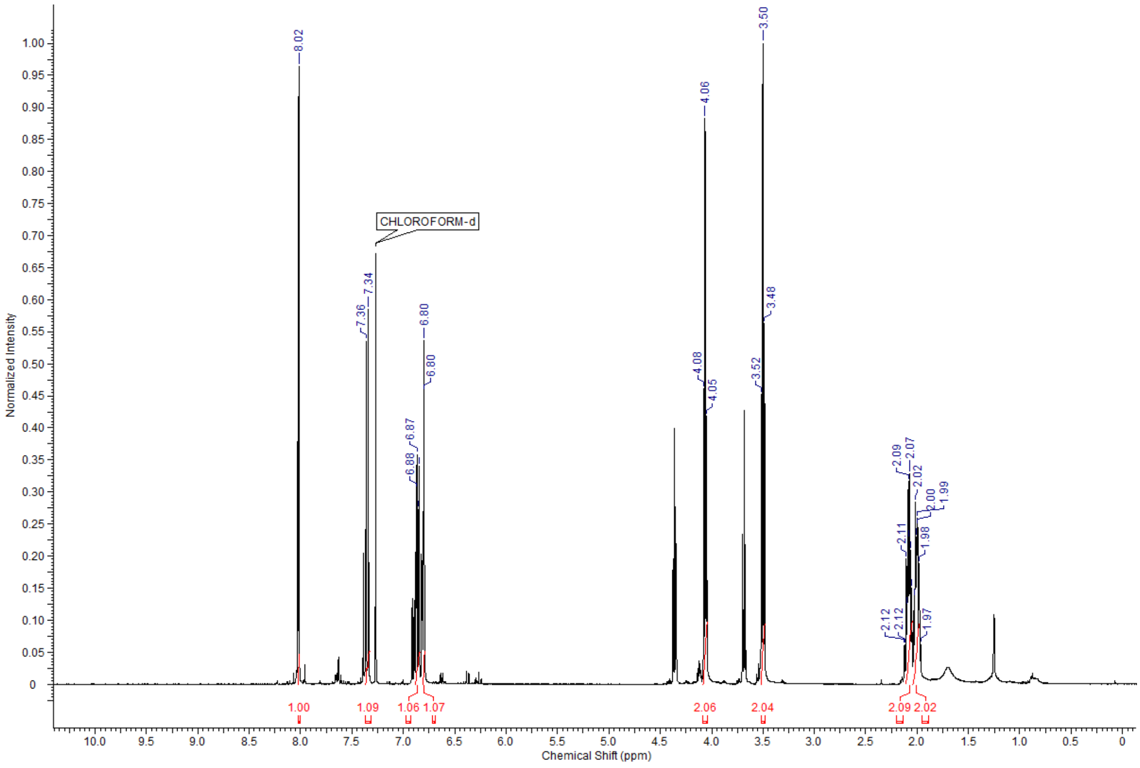

^{13}C NMR espectra of **3c**

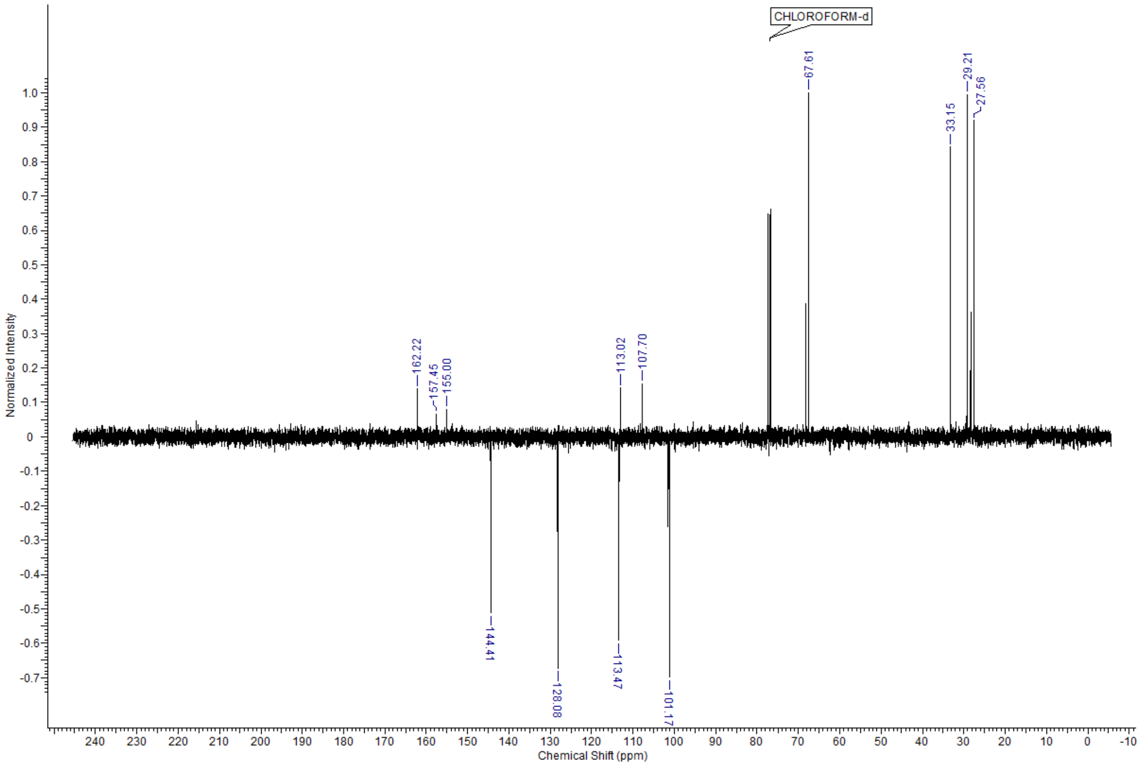

^{1}H NMR spectra of **3d**

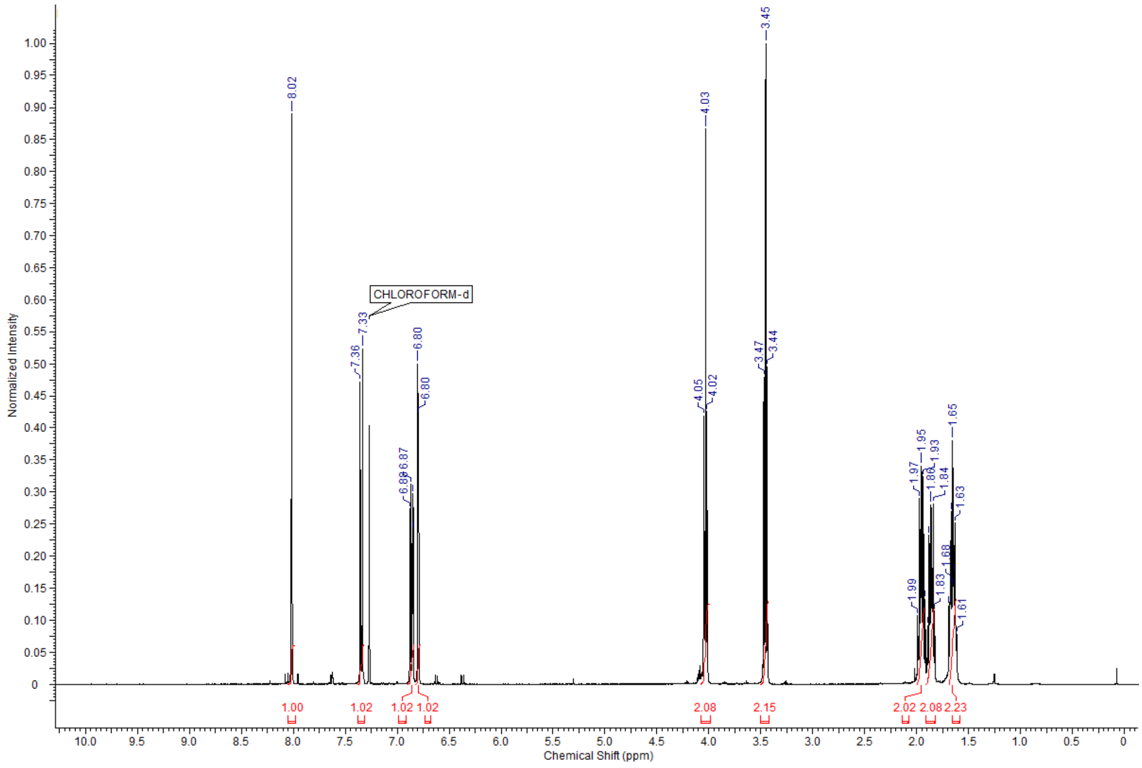

^{13}C NMR espectra of **3d**

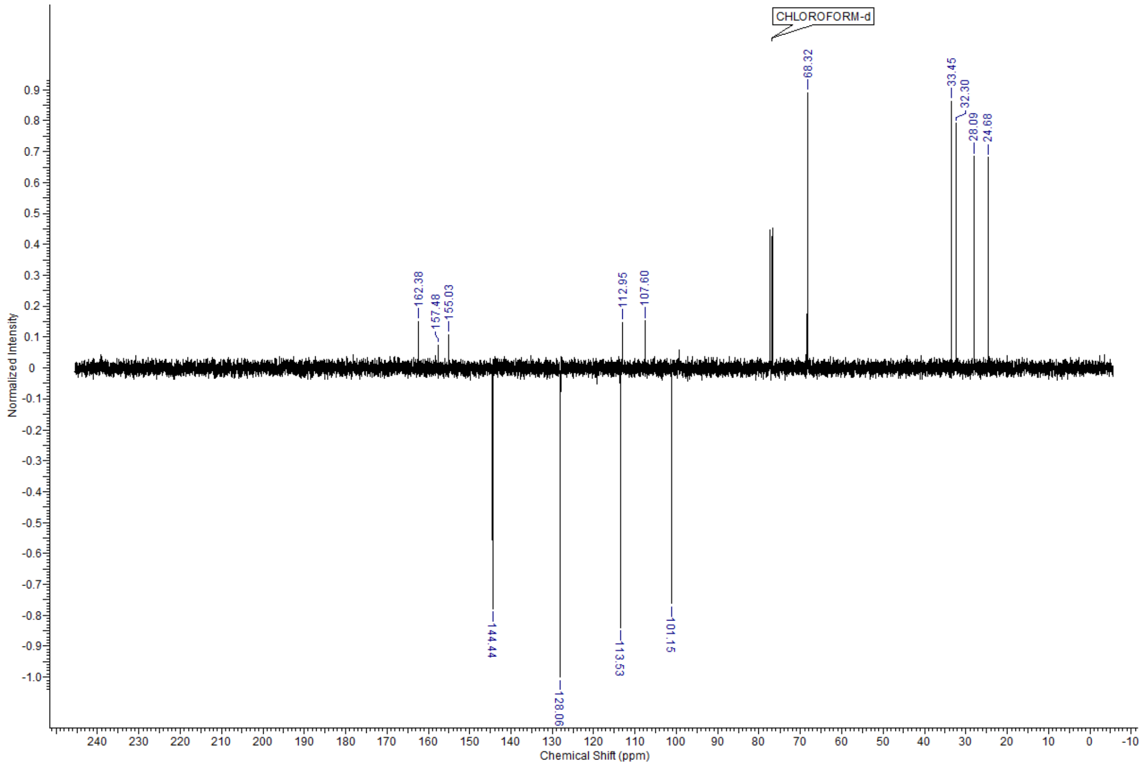

^{1}H NMR spectra of **4a**

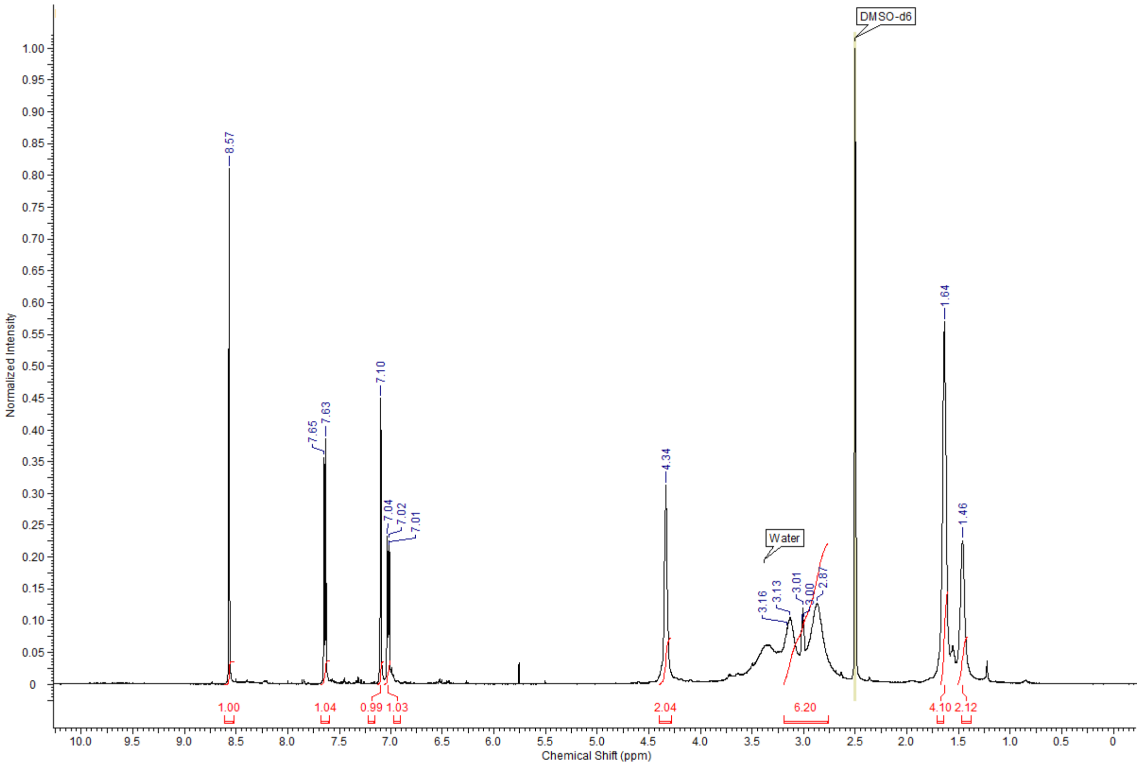

^{13}C NMR espectra of **4a**

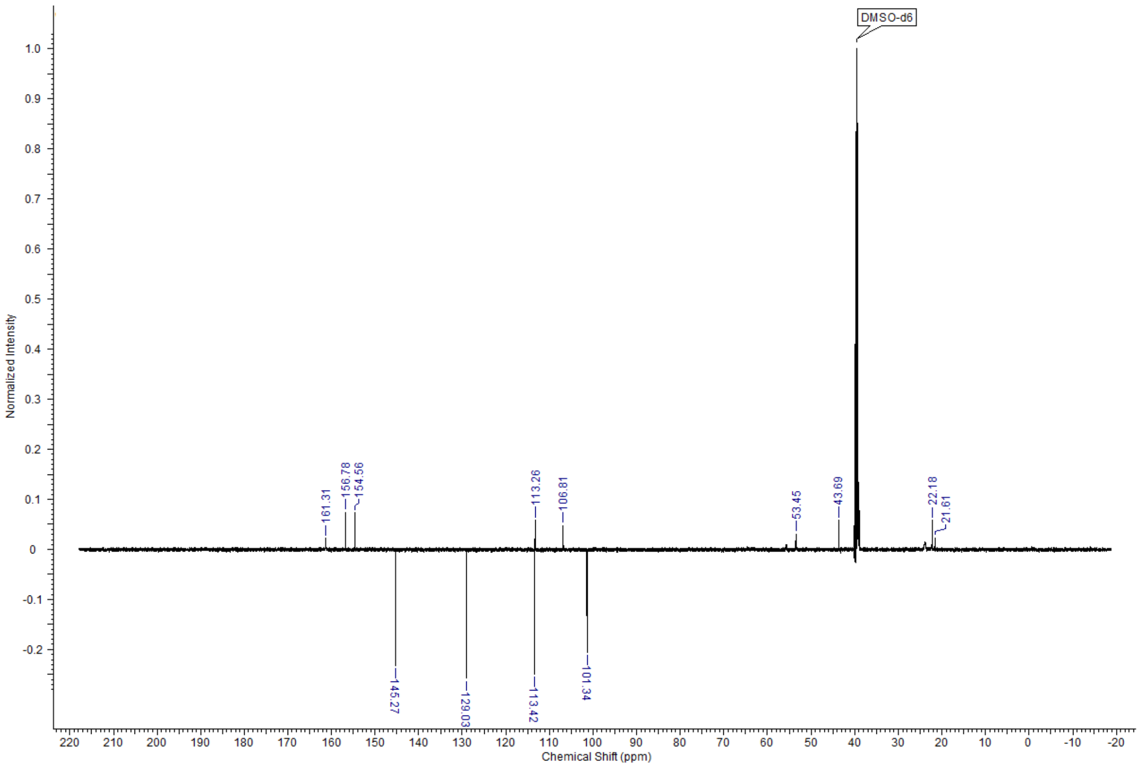

HRMS spectra of **4a**

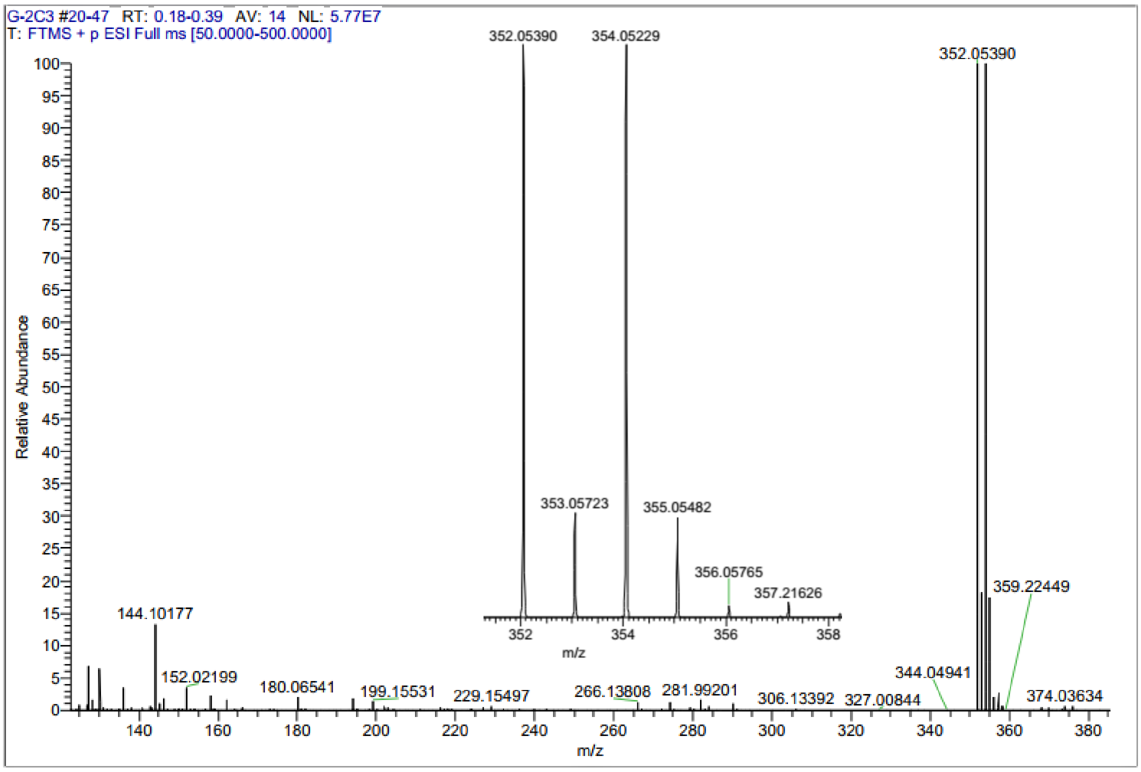

^{1}H NMR spectra of **4b**

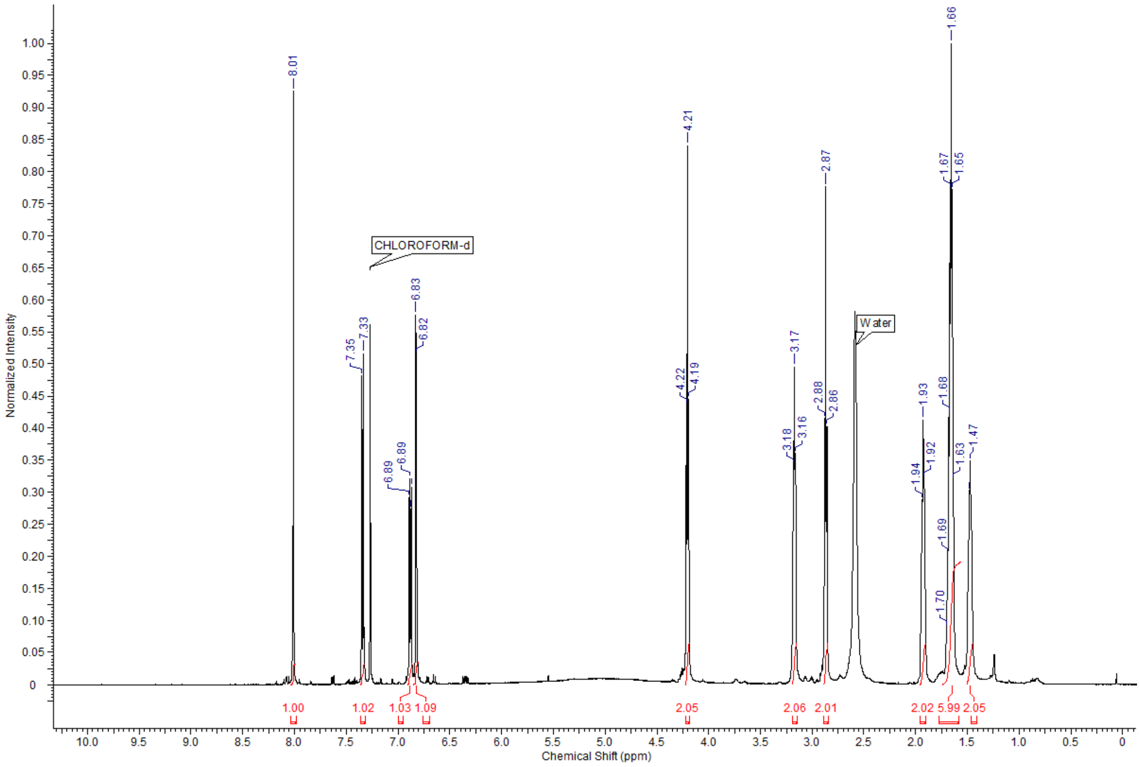

^{13}C NMR espectra of **4b**

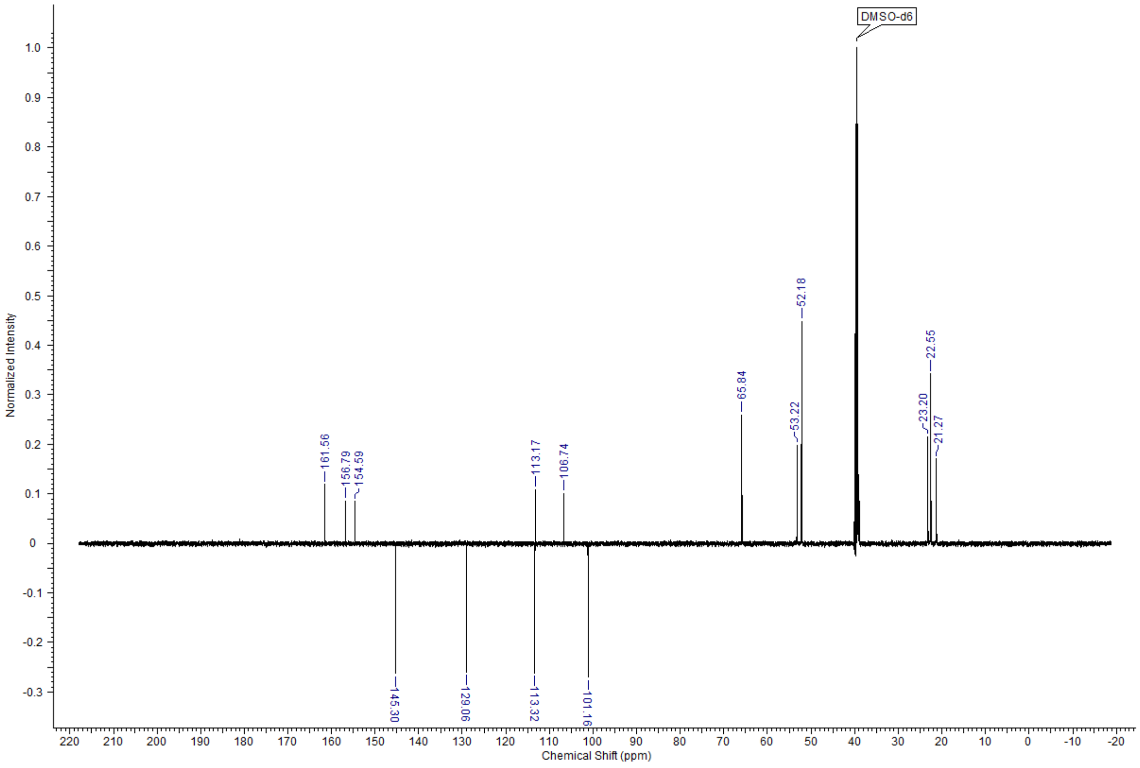

HRMS spectra of **4b**

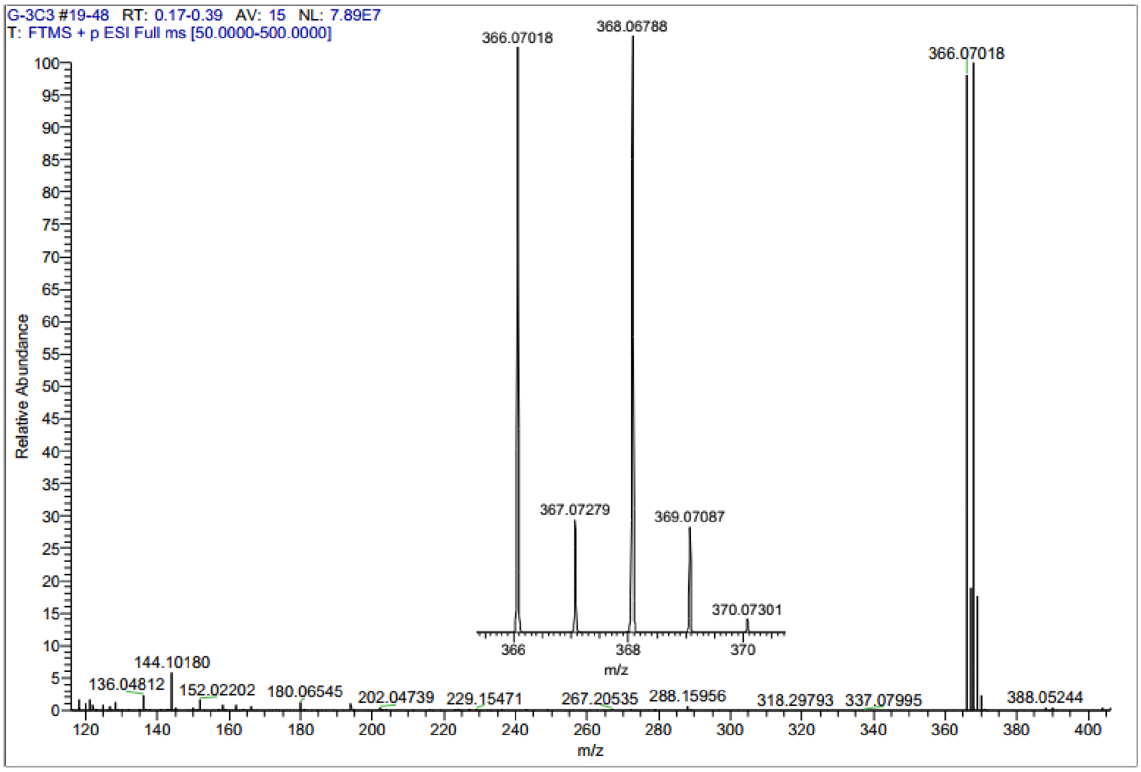

^{1}H NMR spectra of **4c**

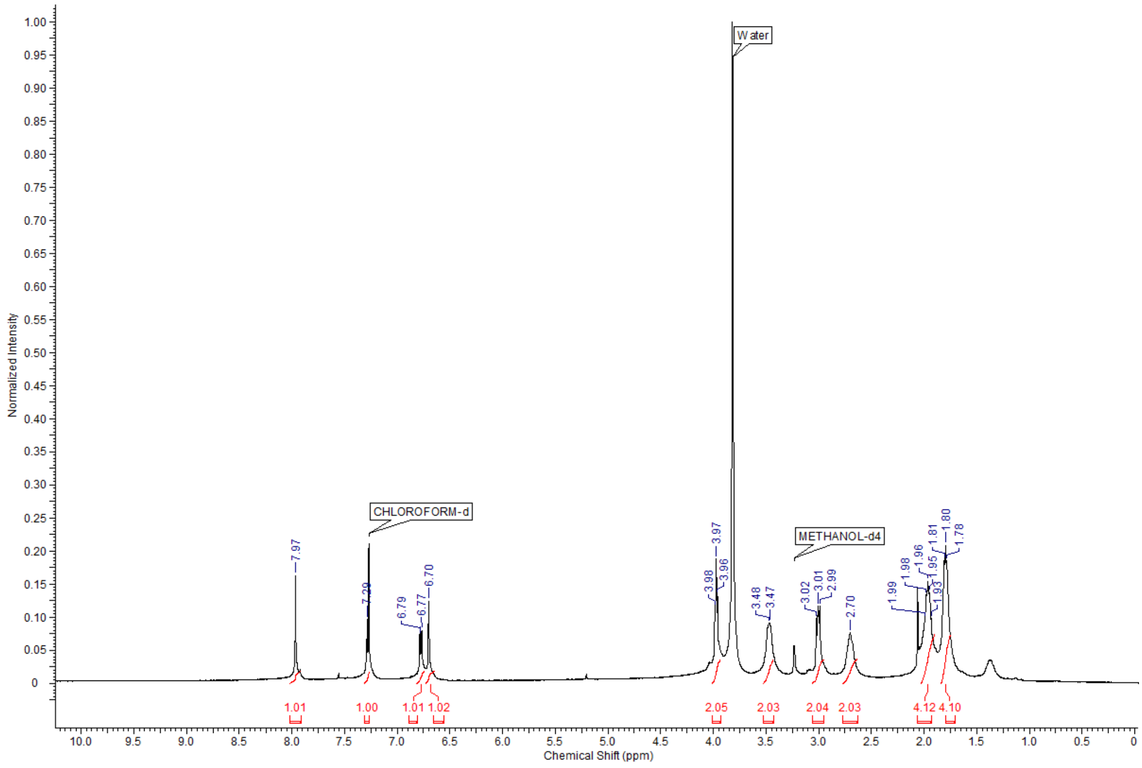

^{13}C NMR espectra of **4c**

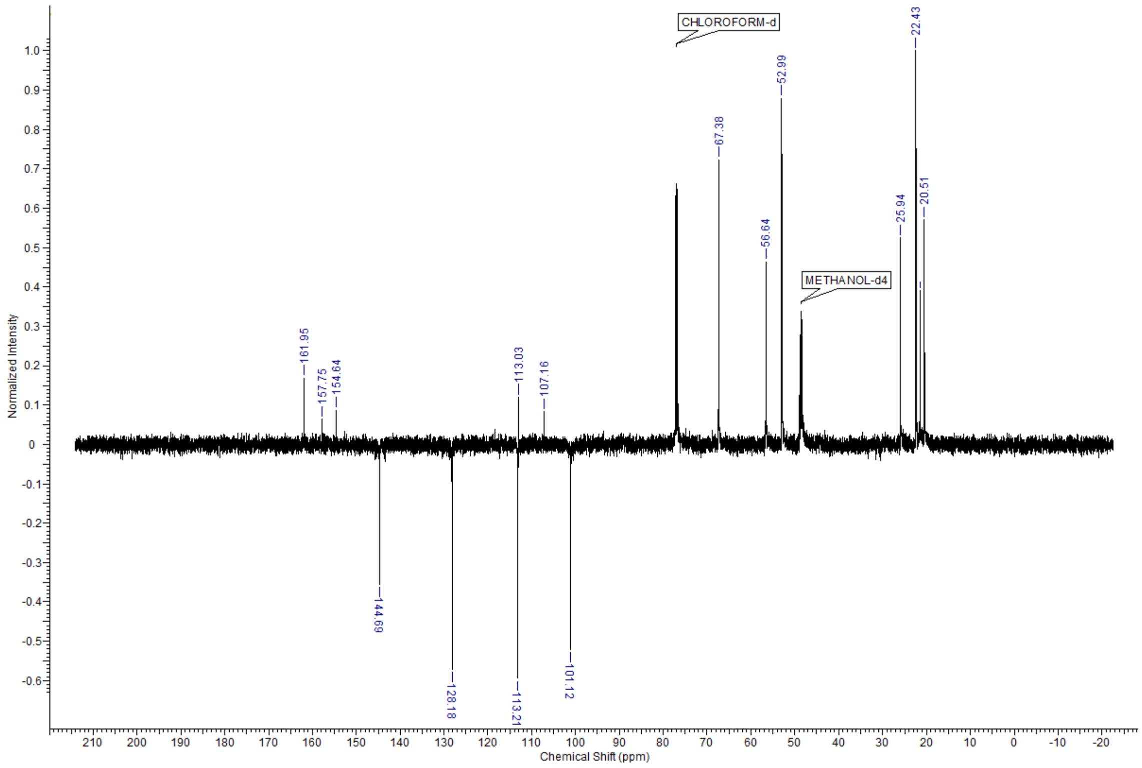

HRMS spectra of **4c**

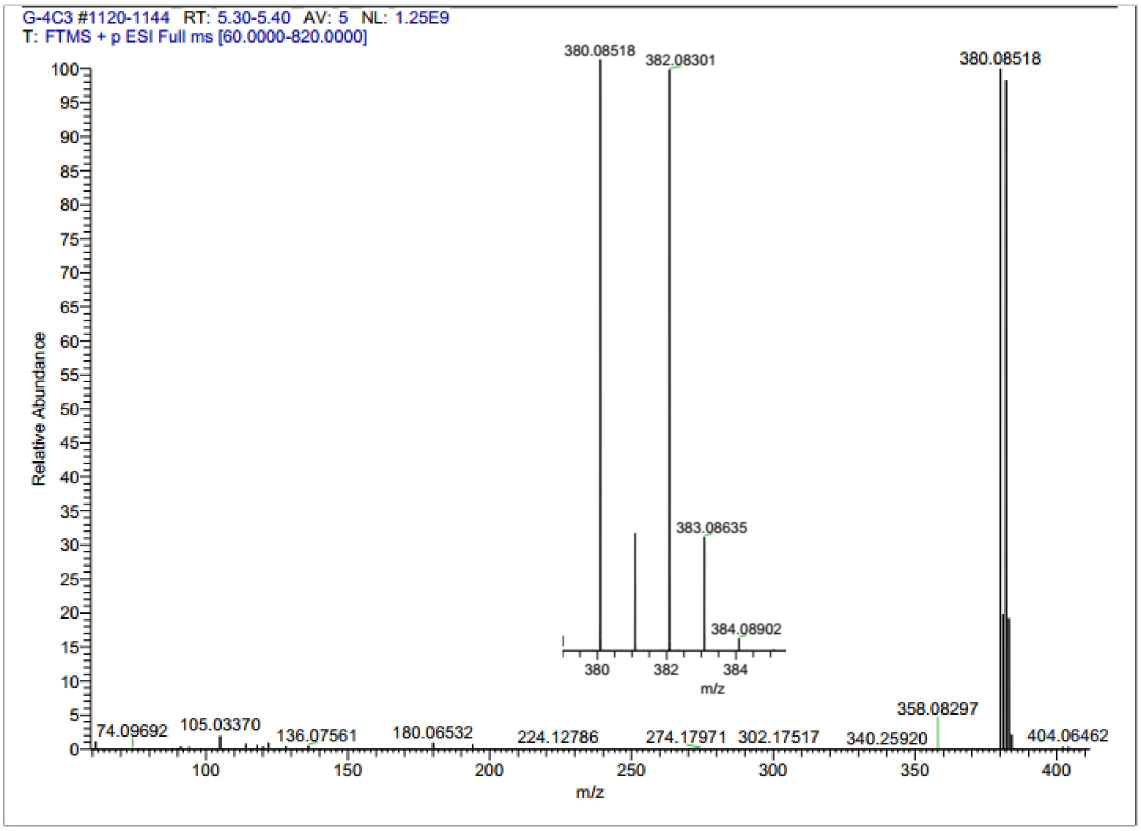

^{1}H NMR spectra of **4d**

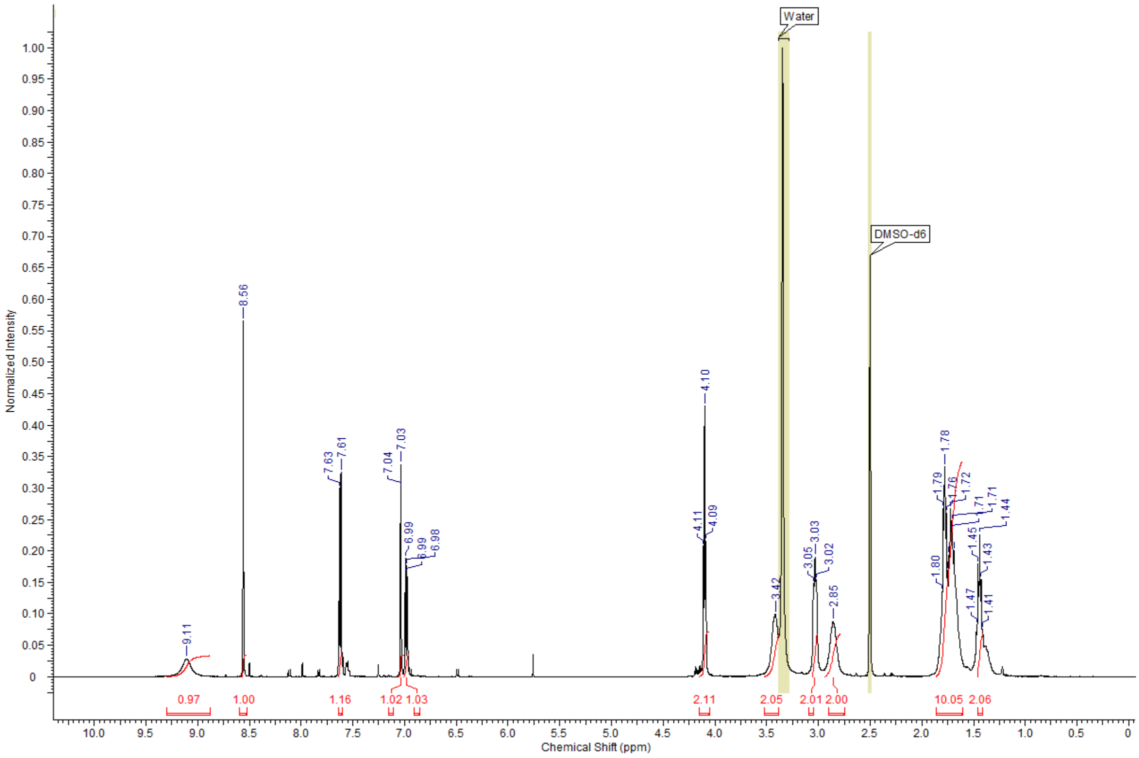

^{13}C NMR espectra of **4d**

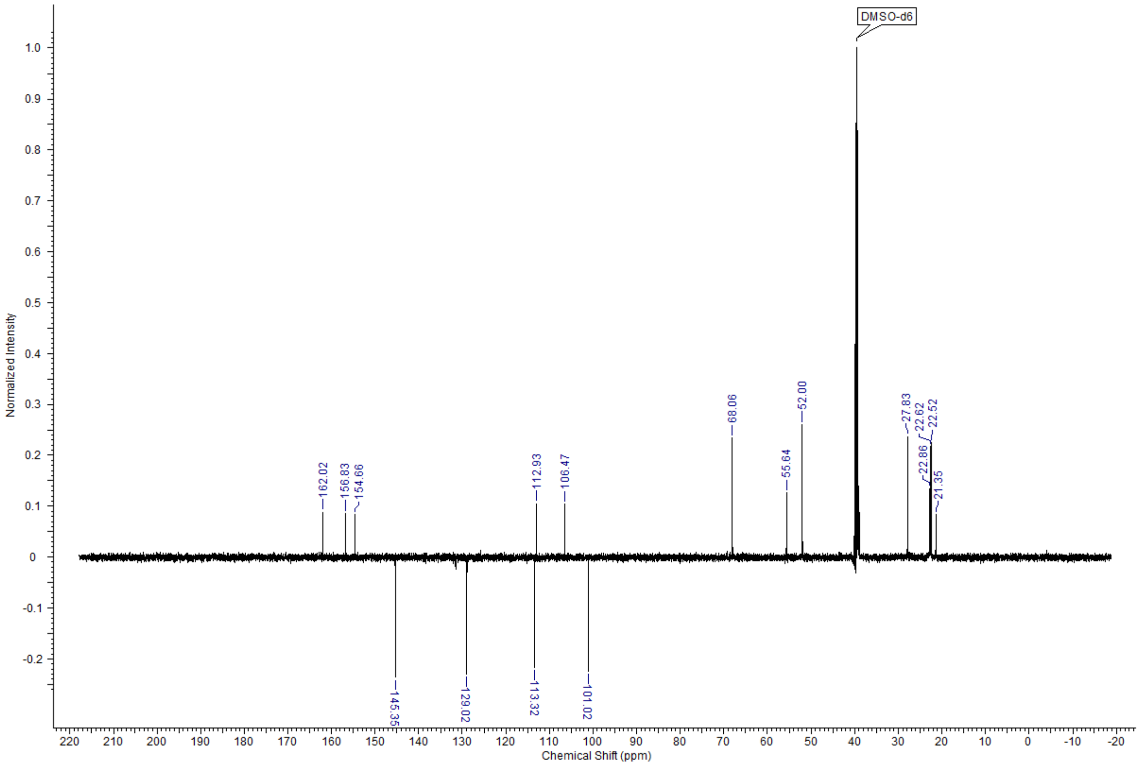

HRMS spectra of **4d**

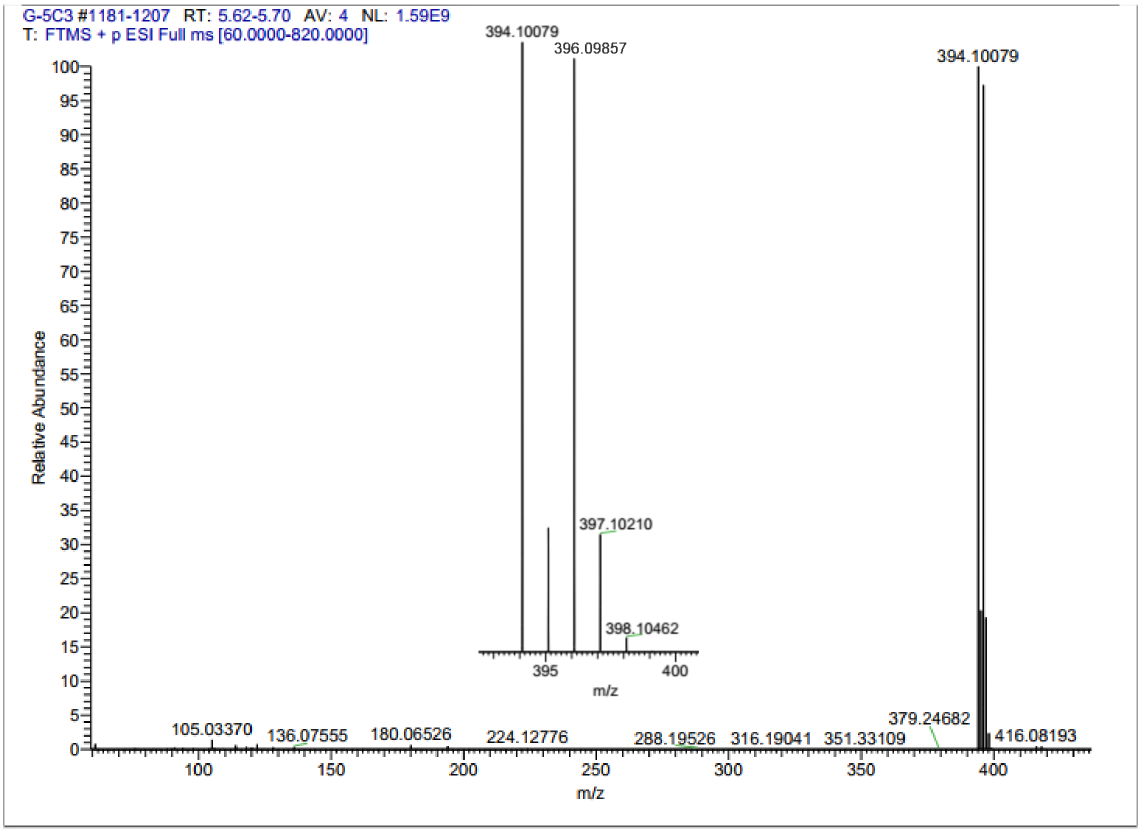

^{1}H NMR spectra of **5a**

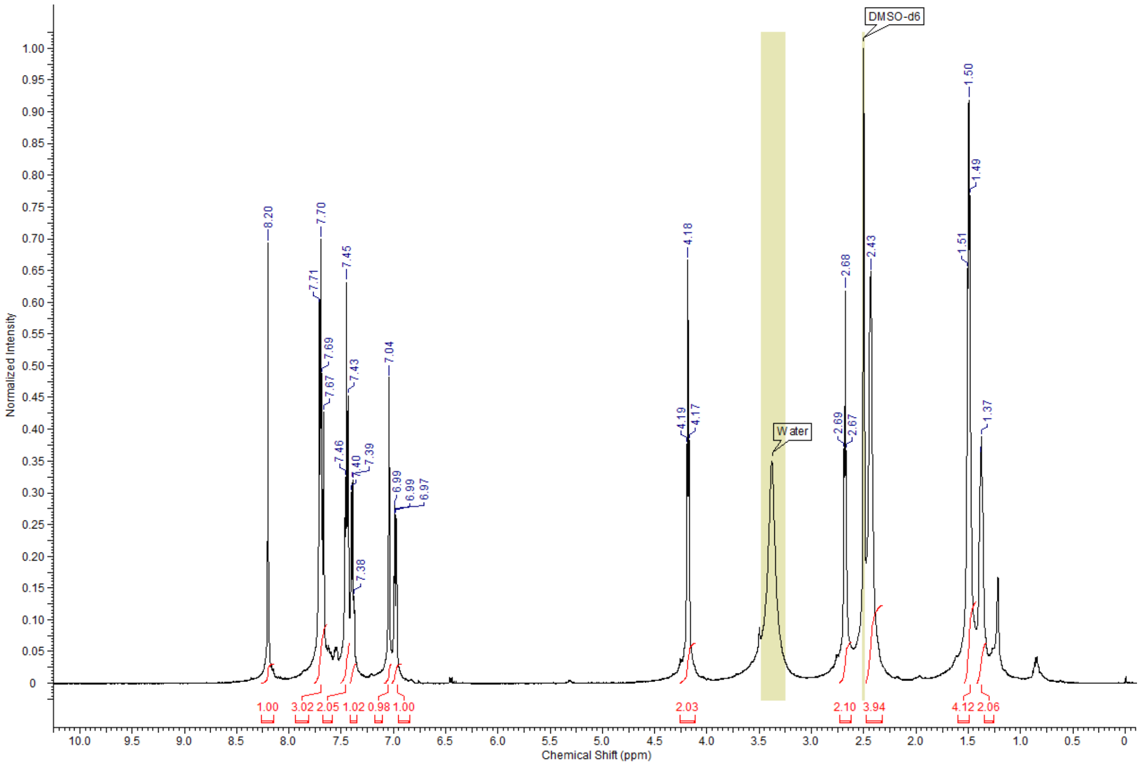

^{13}C NMR espectra of **5a**

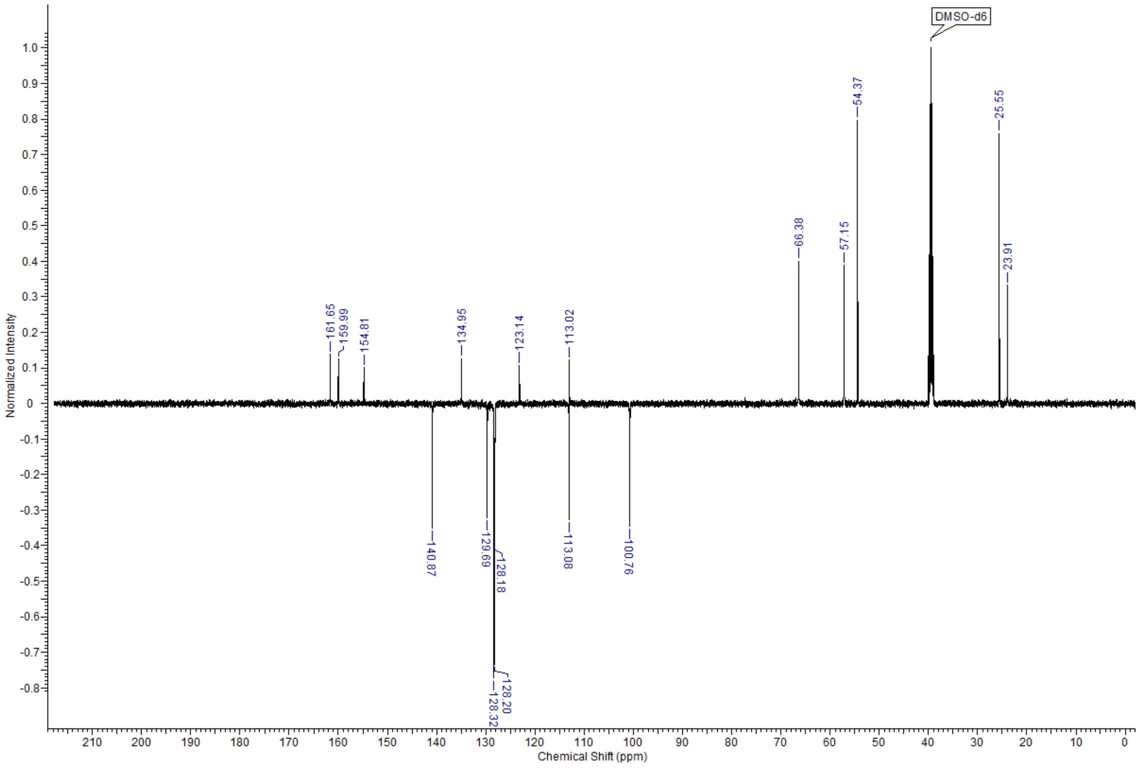

HRMS spectra of **5a**

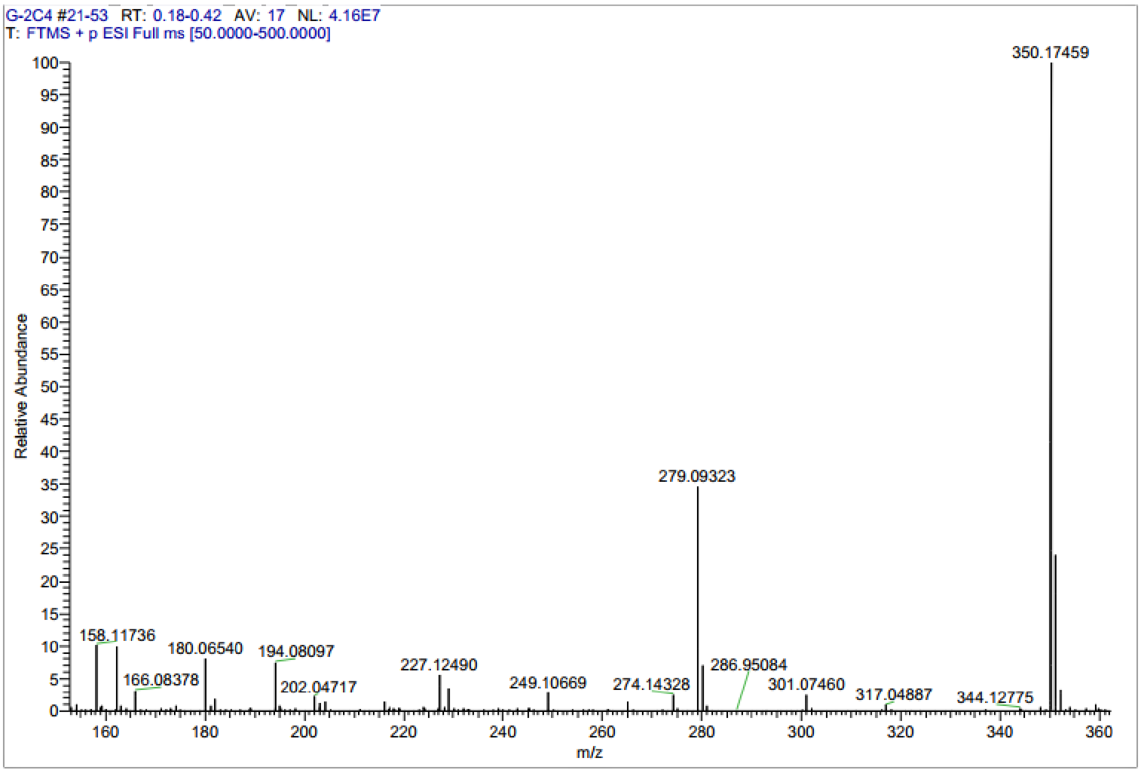

^{1}H NMR spectra of **5b**

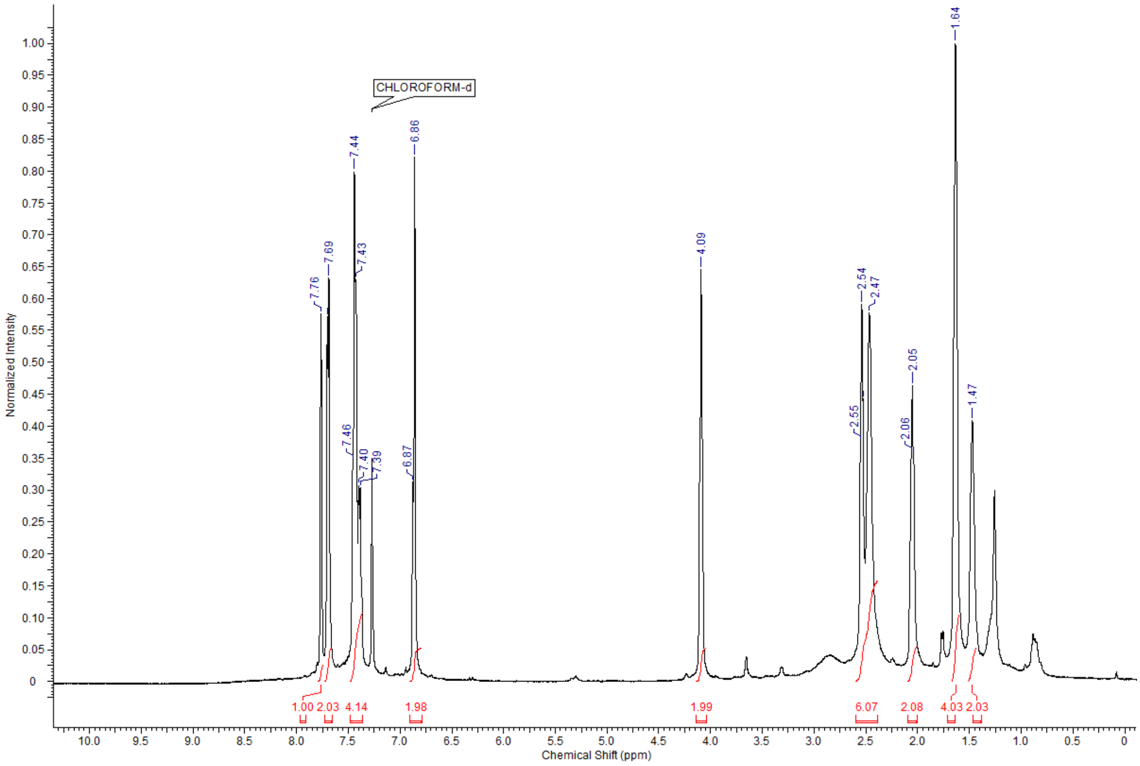

^{13}C NMR espectra of **5b**

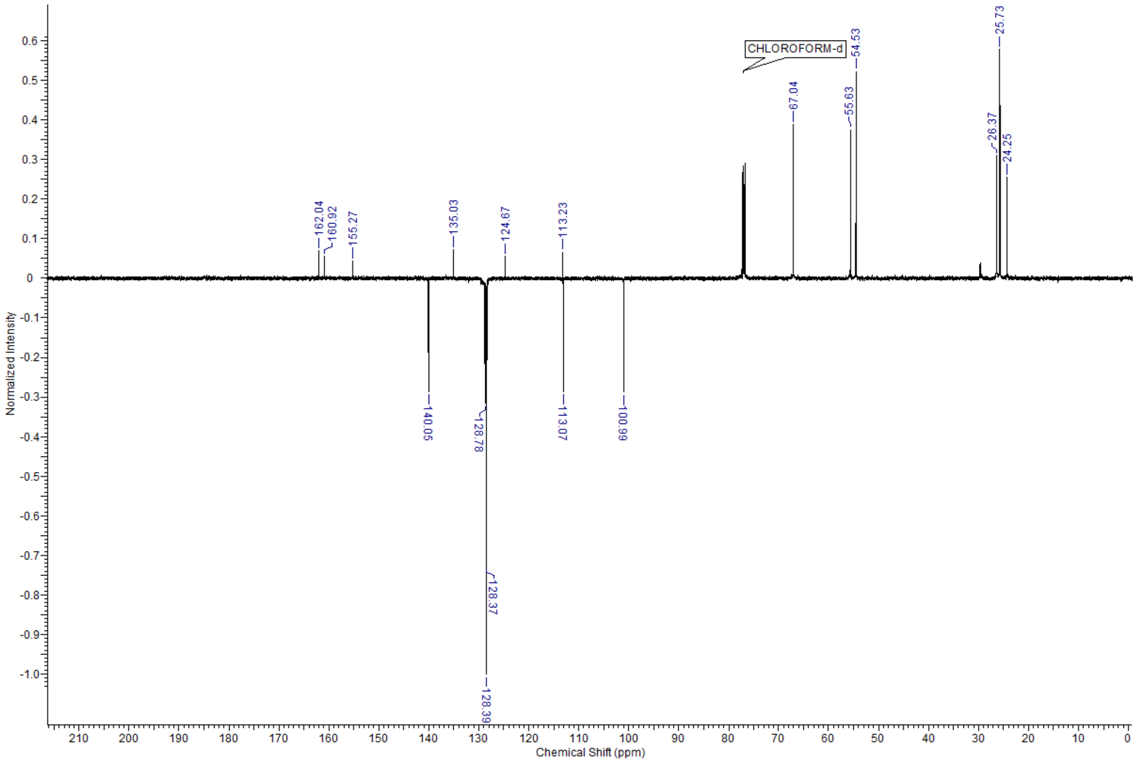

HRMS spectra of **5b**

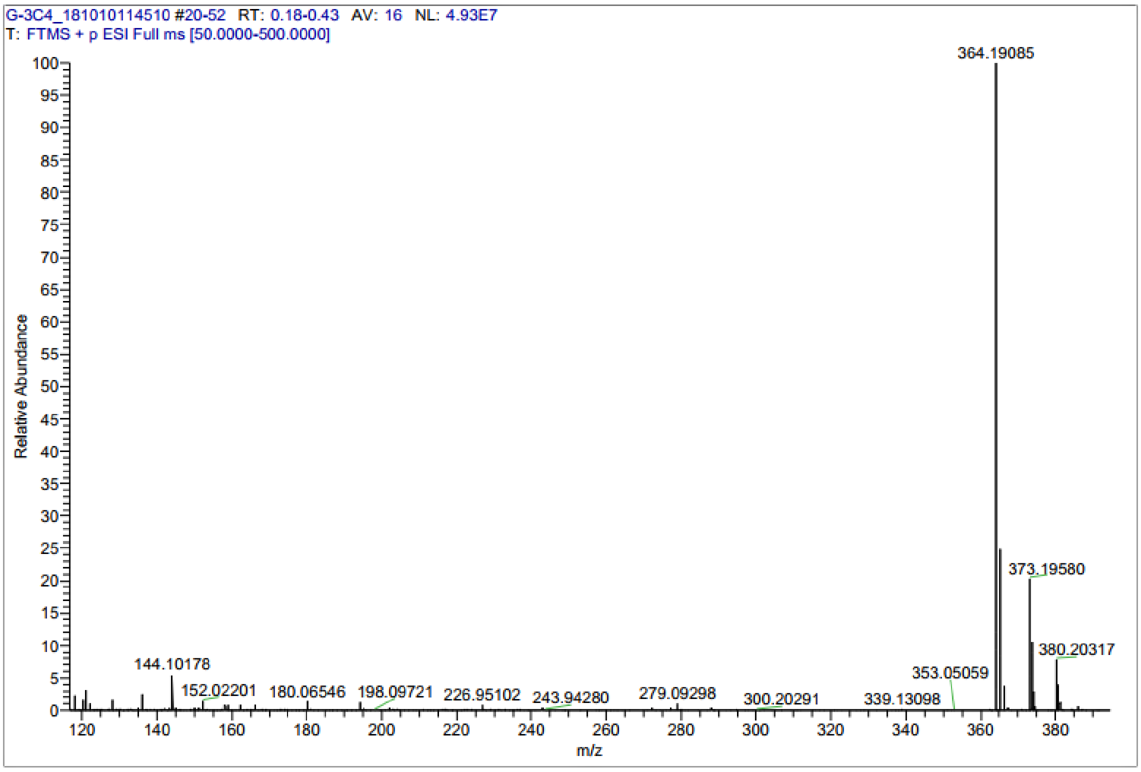

^{1}H NMR spectra of **5c**

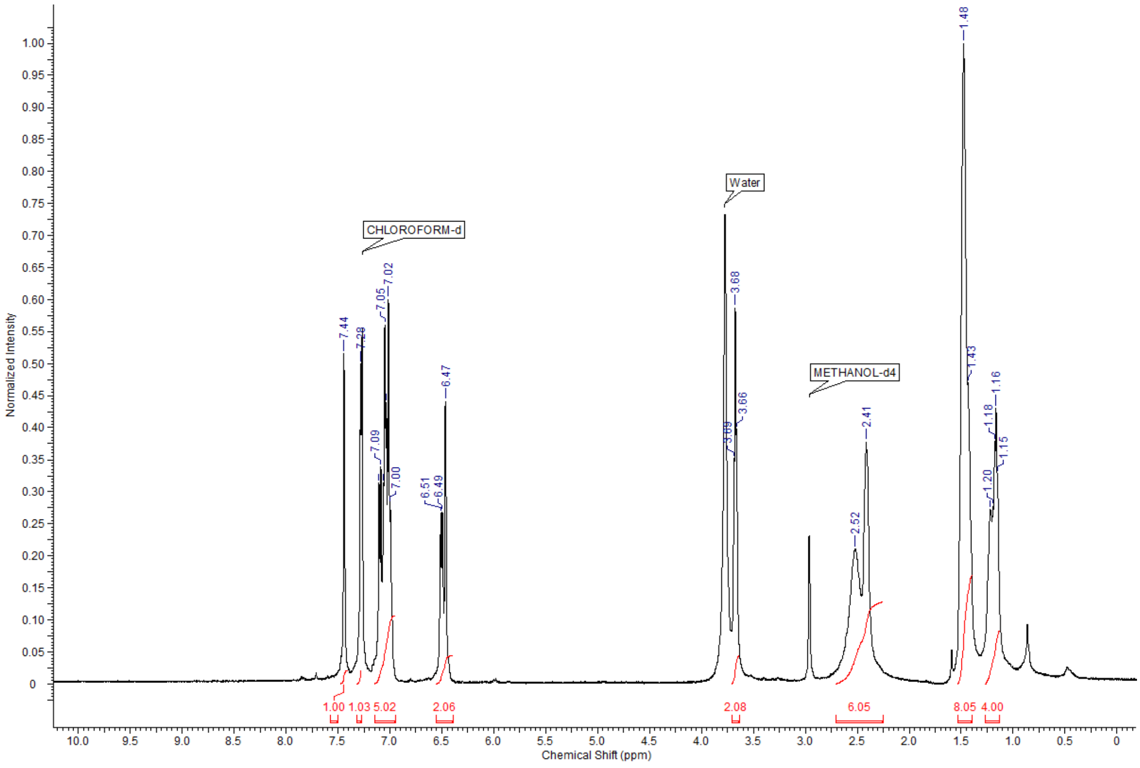

^{13}C NMR espectra of **5c**

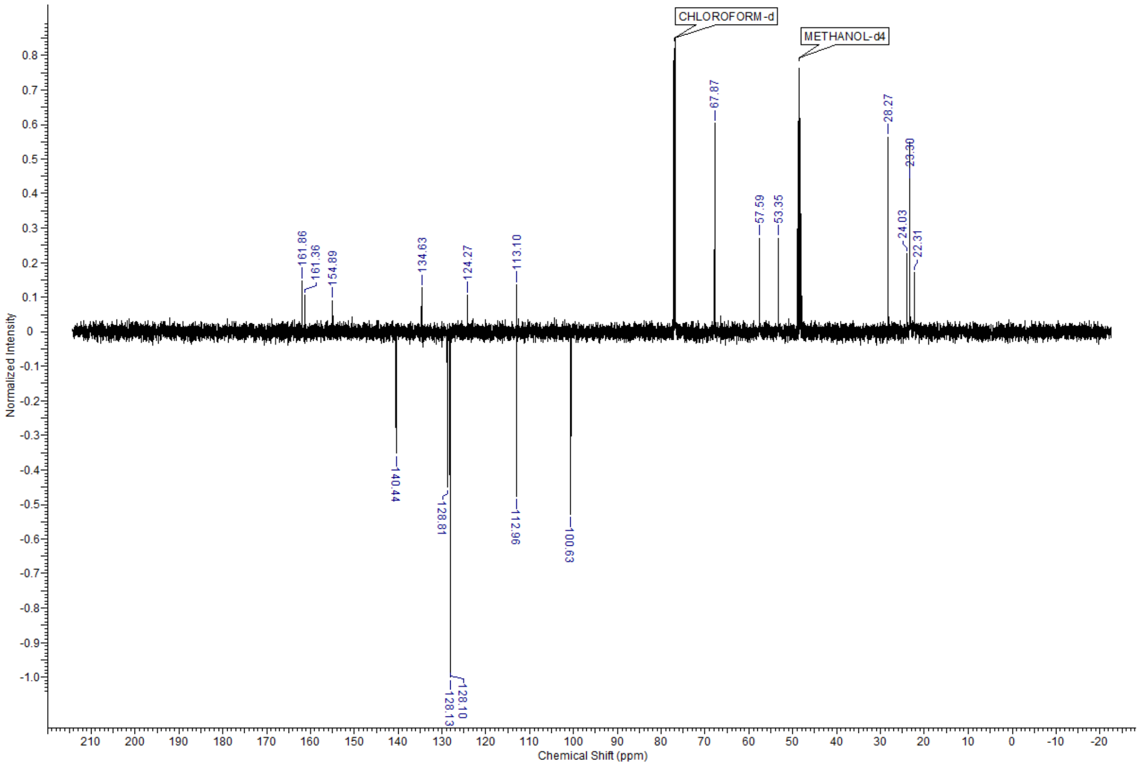

HRMS spectra of **5c**

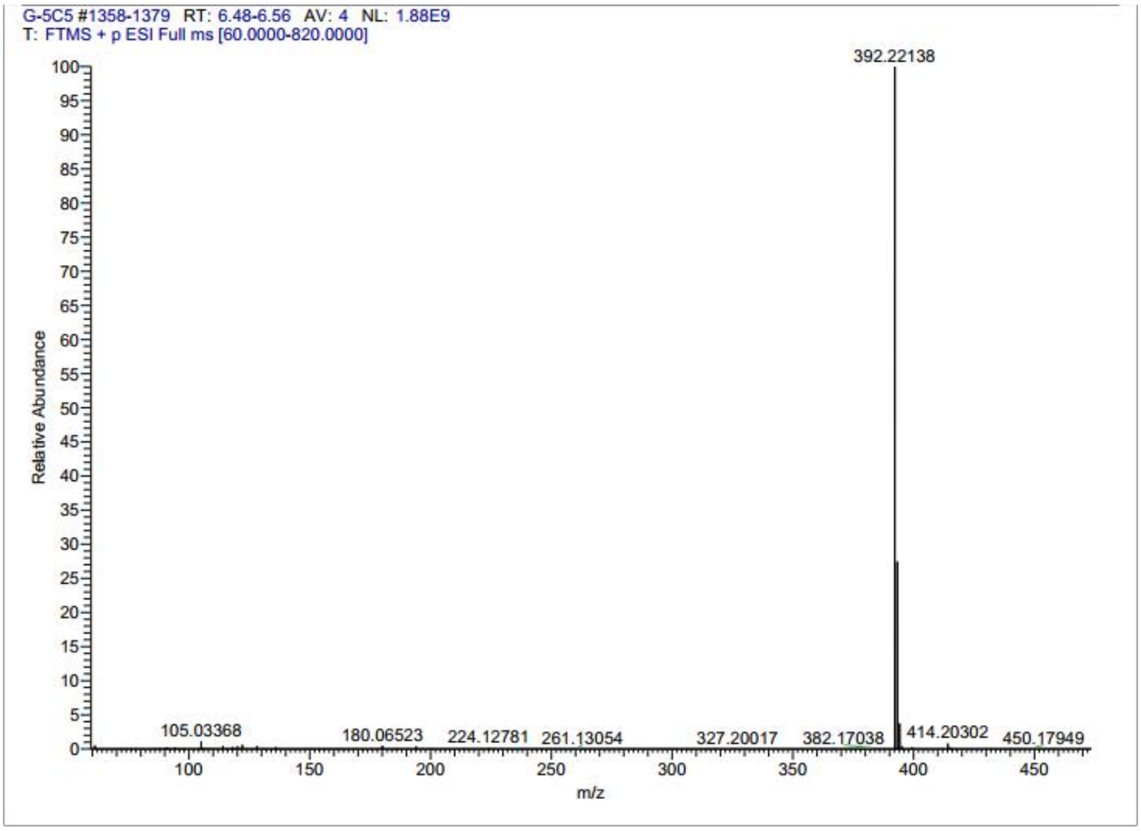

^{1}H NMR spectra of **6a**

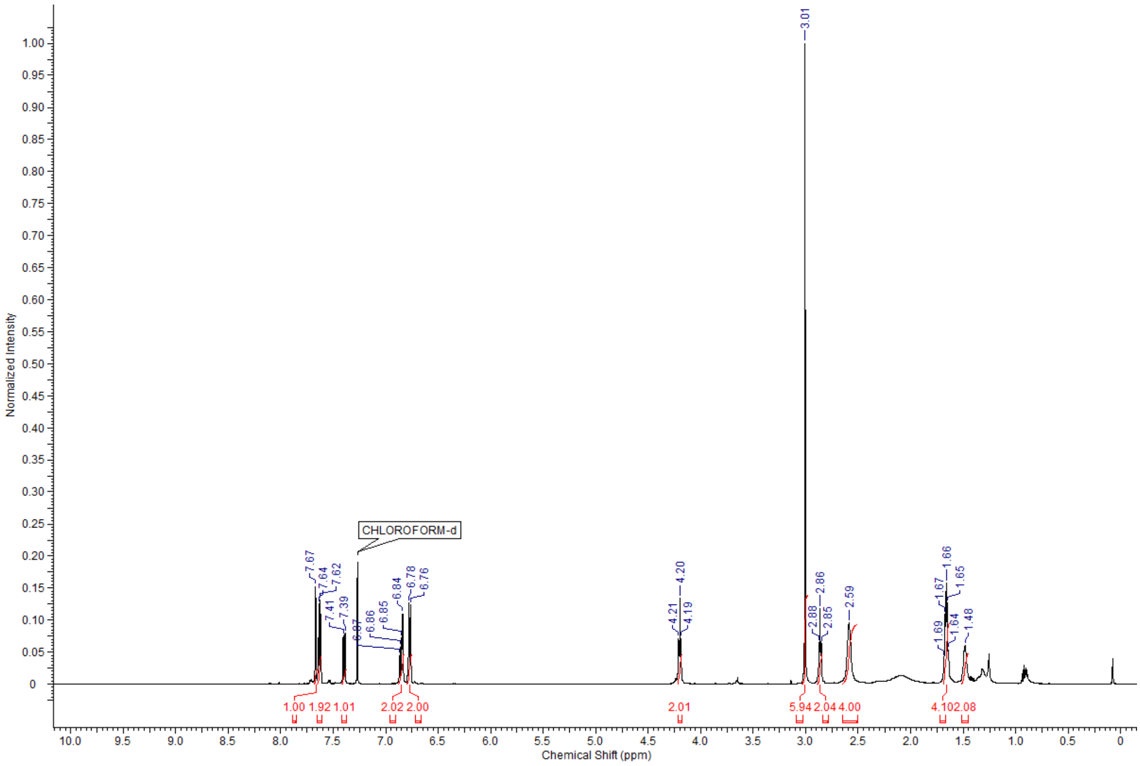

^{13}C NMR espectra of **6a**

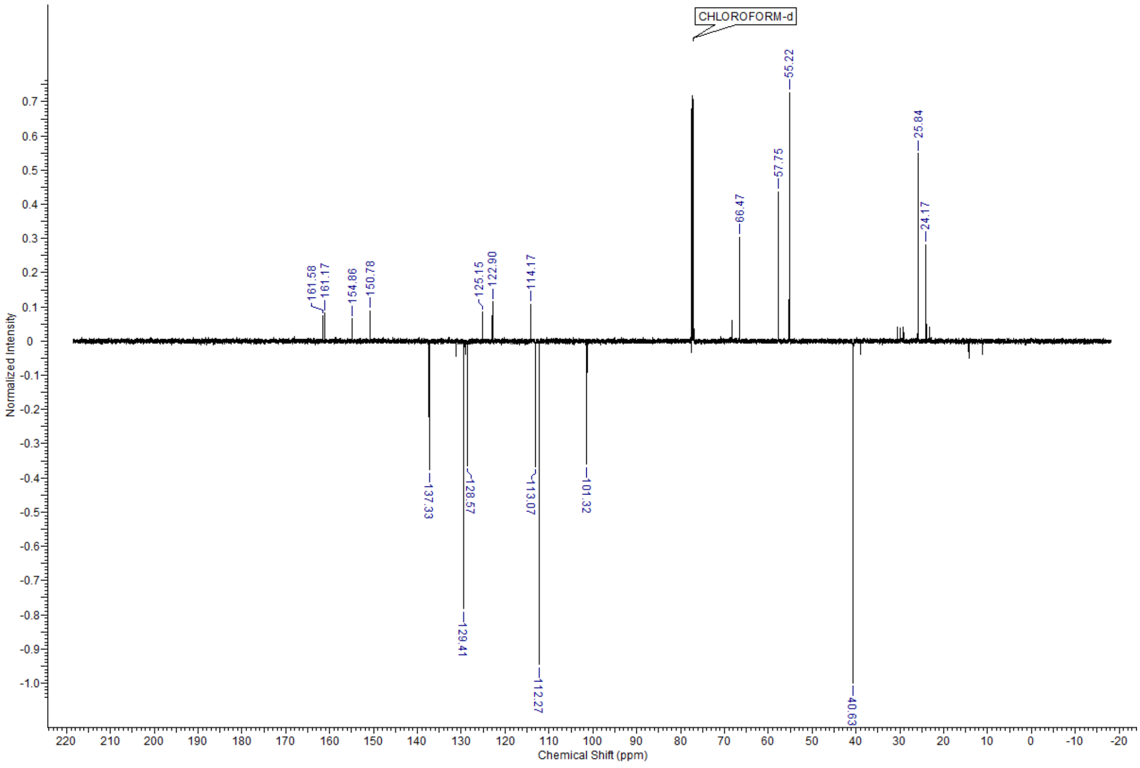

HRMS spectra of **6a**

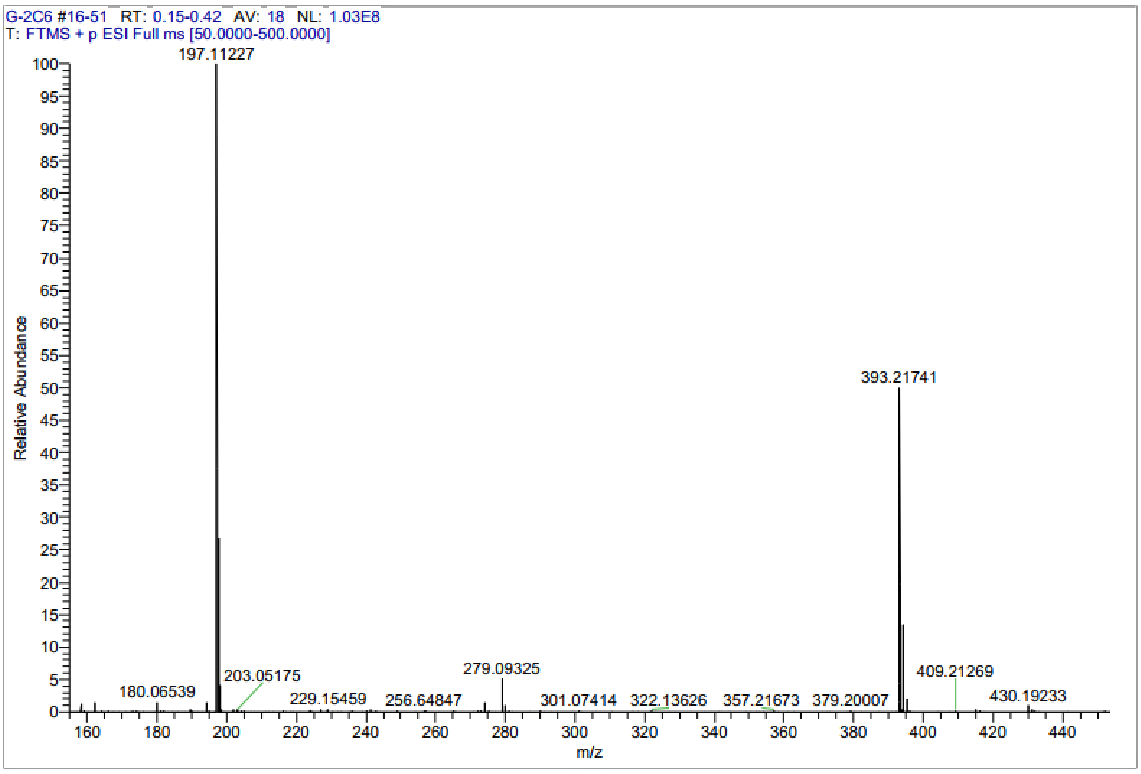

^{1}H NMR spectra of **6b**

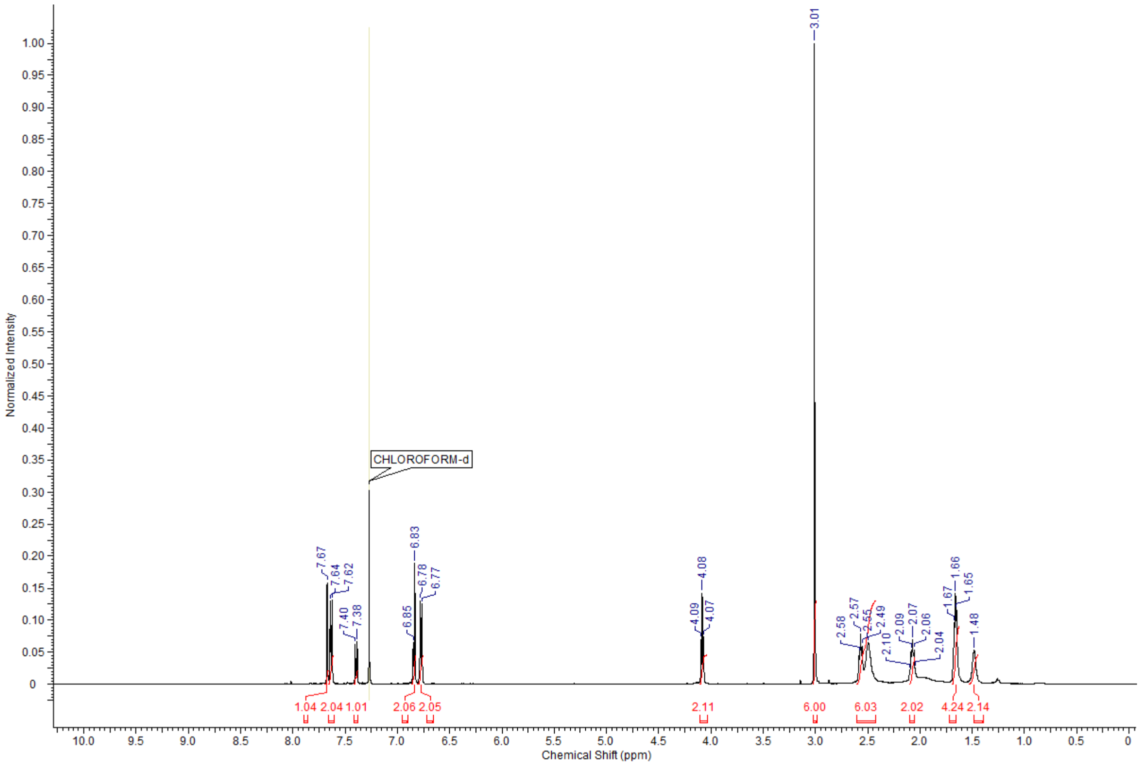

^{13}C NMR espectra of **6b**

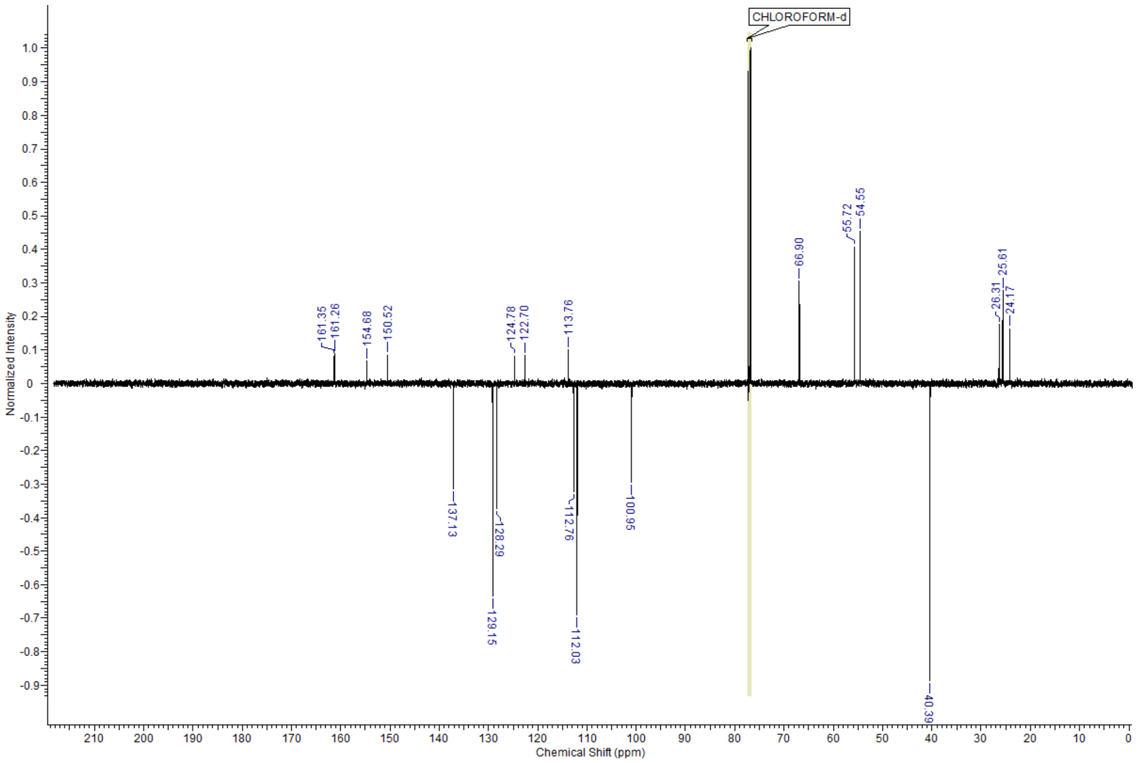

HRMS spectra of **6b**

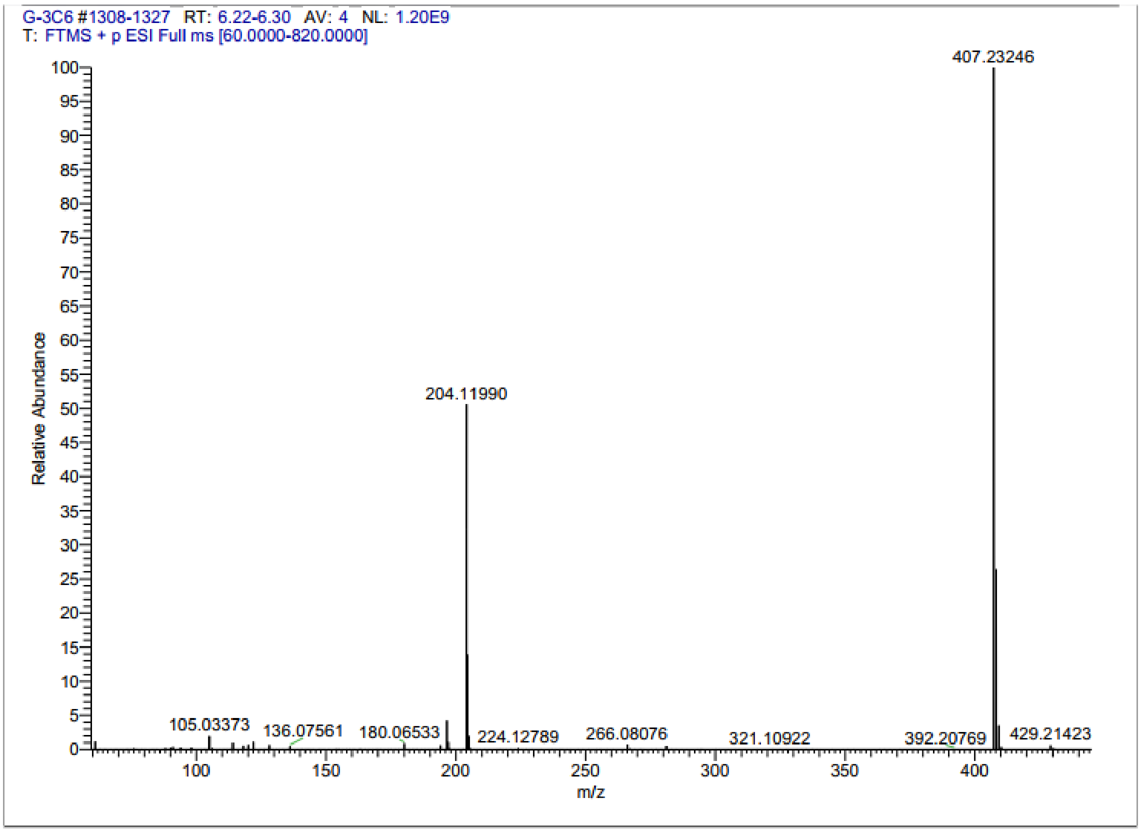

^{1}H NMR spectra of **6c**

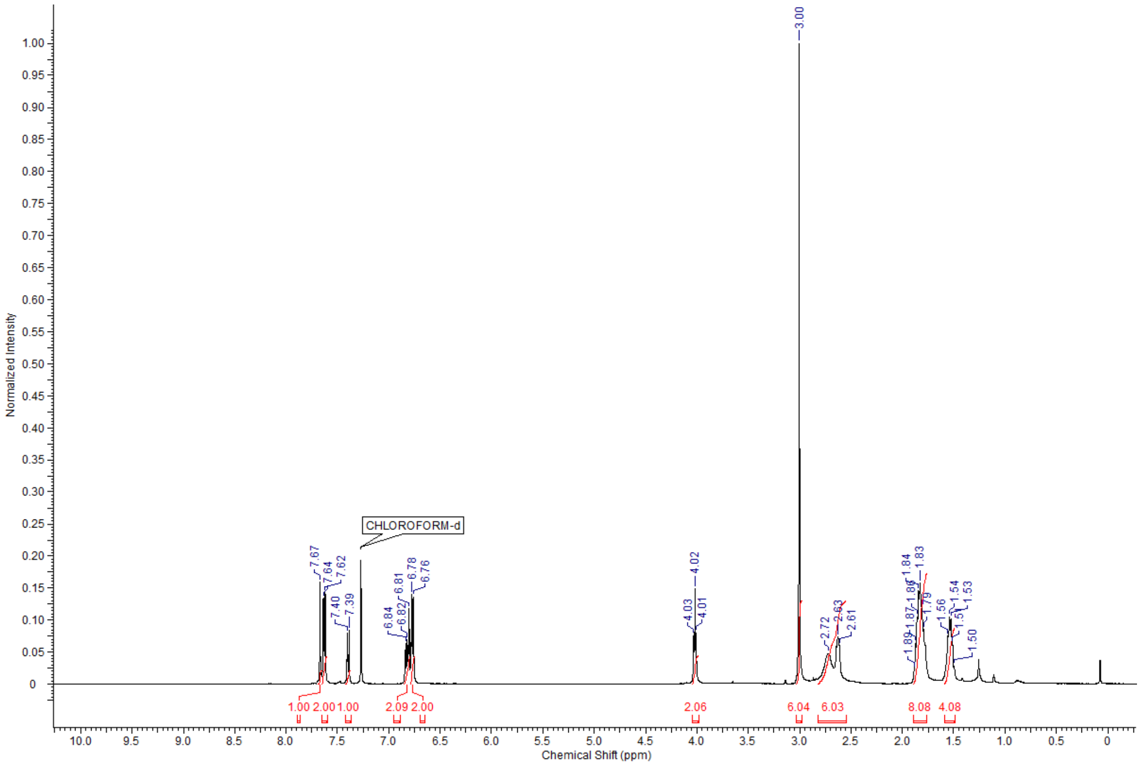

^{13}C NMR espectra of **6c**

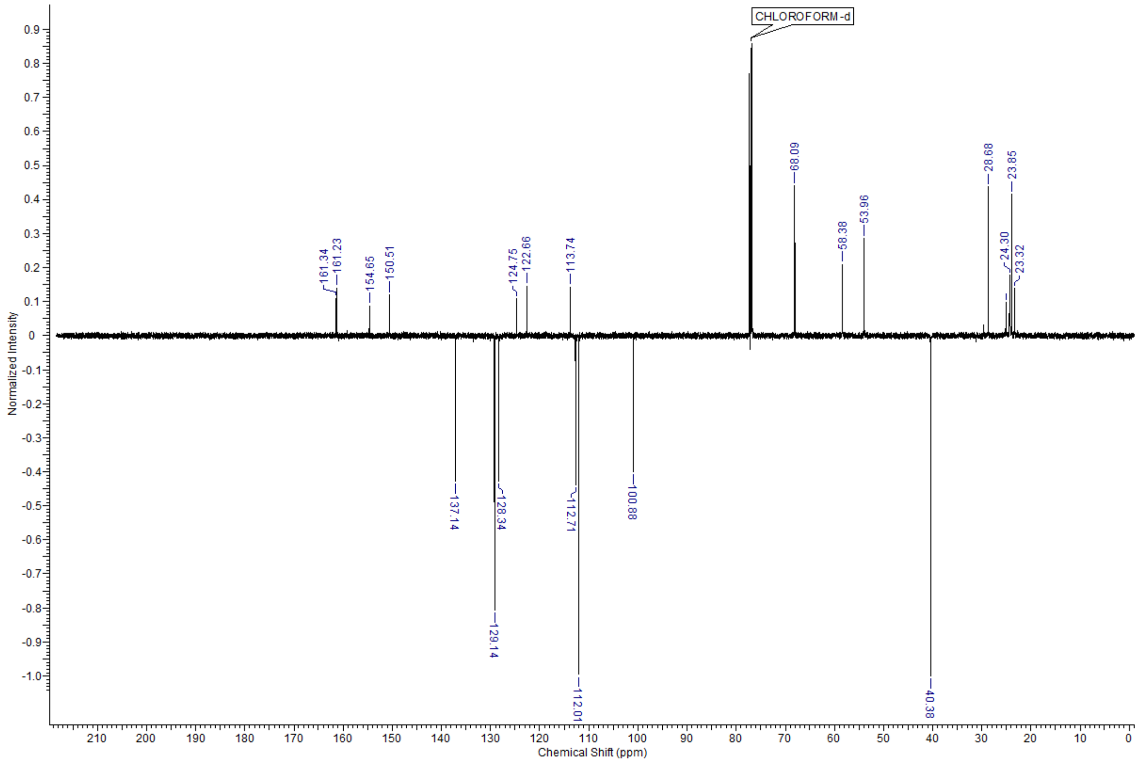

HRMS spectra of **6c**

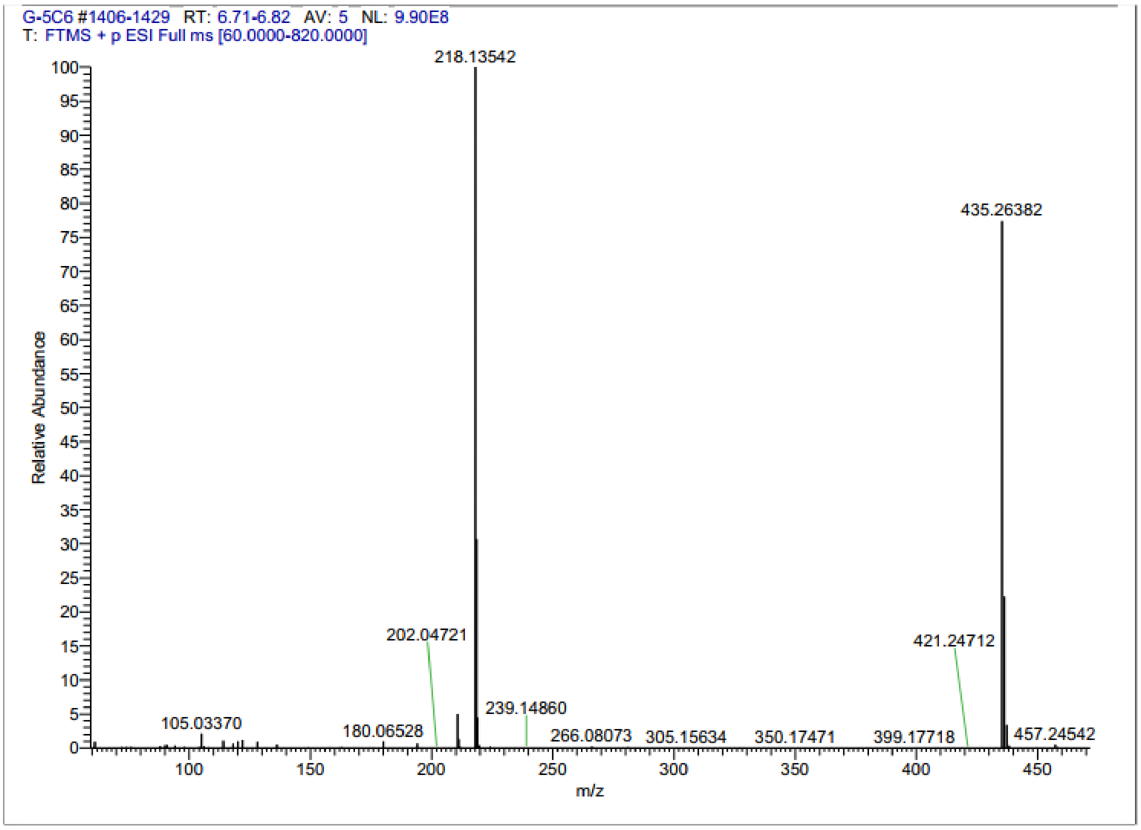

Supplement: Supplemental Material [file IENZ_A_1571270_SM0037.pdf]
